# Recent Advances and Perspectives on Supported Catalysts for Heterogeneous Hydrogen Production from Ammonia Borane

**DOI:** 10.1002/advs.202300726

**Published:** 2023-04-28

**Authors:** Shuyan Guan, Yanyan Liu, Huanhuan Zhang, Ruofan Shen, Hao Wen, Naixin Kang, Jingjing Zhou, Baozhong Liu, Yanping Fan, Jianchun Jiang, Baojun Li

**Affiliations:** ^1^ College of Science Henan Agricultural University 95 Wenhua Road Zhengzhou 450002 P. R. China; ^2^ Research Center of Green Catalysis College of Chemistry School of Physics and Microelectronics Zhengzhou University 100 Science Road Zhengzhou 450001 P. R. China; ^3^ College of Chemistry and Chemical Engineering Henan Key Laboratory of Coal Green Conversion Henan Polytechnic University 2001 Century Avenue Jiaozuo 454000 P. R. China; ^4^ Institute of Chemical Industry of Forest Products CAF National Engineering Lab for Biomass Chemical Utilization Key and Open Lab on Forest Chemical Engineering SFA 16 Suojinwucun Nanjing 210042 P. R. China; ^5^ ISM UMR CNRS N° 5255 Univ. Bordeaux Talence Cedex 33405 France

**Keywords:** ammonia borane, catalyst mechanism, regeneration, standardization, supported catalysts

## Abstract

Ammonia borane (AB), a liquid hydrogen storage material, has attracted increasing attention for hydrogen utilization because of its high hydrogen content. However, the slow kinetics of AB hydrolysis and the indefinite catalytic mechanism remain significant problems for its large‐scale practical application. Thus, the development of efficient AB hydrolysis catalysts and the determination of their catalytic mechanisms are significant and urgent. A summary of the preparation process and structural characteristics of various supported catalysts is presented in this paper, including graphite, metal‐organic frameworks (MOFs), metal oxides, carbon nitride (CN), molybdenum carbide (MoC), carbon nanotubes (CNTs), boron nitride (h‐BN), zeolites, carbon dots (CDs), and metal carbide and nitride (MXene). In addition, the relationship between the electronic structure and catalytic performance is discussed to ascertain the actual active sites in the catalytic process. The mechanism of AB hydrolysis catalysis is systematically discussed, and possible catalytic paths are summarized to provide theoretical considerations for the designing of efficient AB hydrolysis catalysts. Furthermore, three methods for stimulating AB from dehydrogenation by‐products and the design of possible hydrogen product‐regeneration systems are summarized. Finally, the remaining challenges and future research directions for the effective development of AB catalysts are discussed.

## Graphical abstract

A summary of the preparation process and structural characteristics of supported catalysts is presented. The mechanism and catalytic paths of ammonia borane (AB) hydrolysis are discussed for the designing of AB hydrolysis catalysts. Methods for stimulating AB from dehydrogenation by‐products and hydrogen product‐regeneration systems are summarized. The remaining challenges and future directions for the effective development of AB catalysts are discussed.

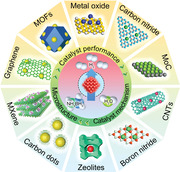

## 1 Introduction

### 1.1 Hydrogen Energy–Liquid Hydrogen Storage

Environmental pollution and high carbon emissions from fossil fuels are unsustainable for future development.^[^ ^1^ ^]^ Therefore, it is imperative to examine high‐energy, renewable, clean, and environmentally friendly energy sources.^[^ ^2^ ^]^ Hydrogen energy is both a clean and low‐carbon new energy source and an energy storage medium, thus it will play an irreplaceable role in the future energy structure.^[^ ^3^ ^]^ Compared to common energy storage supports such as supercapacitor battery compressed air, hydrogen has evident advantages regarding storage capacity and charging time.^[^ ^4^ ^]^ In terms of specific energy, hydrogen has almost three times the specific energy of gasoline. Moreover, hydrogen energy is suitable for medium to large‐scale energy storage applications.^[^ ^5^ ^]^ The hydrogen energy industry chains include hydrogen production, storage, transportation, and utilization, with storage being a key problem toresolve.^[^ ^6^ ^]^ Therefore, the development of new materials that can provide high‐quality and volume‐density hydrogen with appropriate thermodynamic and kinetic properties is of great concern. The most efficient and safest way to store hydrogen is to use a solid medium such as an adsorbent material or hydride. Liquid hydrogen storage technology provides a higher energy density for hydrogen storage than for gas or liquid hydrogen storage tank systems.^[^ ^7^ ^]^ Additionally, liquid hydrogen storage has the following advantages: First, no special hydrogen production facilities or devices need to be built, nor are corresponding sites needed. Second, large‐scale hydrogen compression and storage facilities are not required. Third, there are no constraints on high‐pressure transportation. Hydrogen can be produced on‐site at hydrogenation stations and does not require high‐pressure transport. Finally, on‐site independent hydrogen production is possible, each hydrogenation station can produce hydrogen according to the needs. Fuel cell vehicles use liquid hydrogen safe, efficient, and low‐cost.

### 1.2 Physical and Chemical Properties of AB

**Figure 1:**
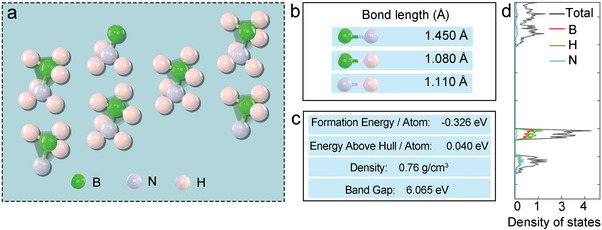
a) Structure of AB, b) bond length of B—N, B—H, B—H, c) physicochemical properties of AB, d) density of states.

Ammonia borane (NH_3_BH_3_ or AB) is a simple molecular hydride, whose structure is depicted in **Figure** **1**. The bond lengths of the B—N, B—H, and N—H bonds are 1.450, 1.080, and 1.110 Å, respectively (Figure 1b). The atomic formation energy for AB is ‐0.326 eV, its energy above hull is 0.040 eV, and its band gap is 6.065 eV (Figure 1c). The densities of the AB states are shown in Figure 1d. As a representative material for liquid hydrogen storage, AB displays several advantages: high weight and volume density, nontoxicity, moderate decomposition temperature, and a white crystalline appearance with a density of 0.76 g cm^−3^ at room temperature.^[^ ^8^ ^]^ AB is highly stable in air, soluble in water and other polar solvents, and is a suitable solid hydrogen storage material. Moreover, AB is not only lightweight and has a high hydrogen storage capacity (19.6 wt%), but also comprises only non‐toxic elements (B, N, H) that are inexpensive and widely accessible. Compared to other hydrogen storage materials, AB does not require a conventional hydrogen production infrastructure, large‐scale hydrogen compression and storage, or high‐pressure transport. Hydrogen stored in AB can be released via pyrolysis (in the solid state) or metal‐catalyzed reactions in protic solvents,^[^ ^9^ ^]^ such as water (catalytic hydrolysis^[^ ^10^ ^]^) or methanol (nonprotic solvent decomposition).^[^ ^11^ ^]^ Under various operating conditions, AB thermolysis can rapidly provide high‐quality hydrogen fuel by heating composite materials.^[^ ^9^ ^]^ However, the practical application of AB thermolysis is significantly impeded by the fact that three primary requirements have not yet been resolved: 1) the dehydrogenation temperature needs to be reduced to the fuel cell operating temperature of 85 °C, 2) refining H_2_ release kinetics at this temperature, and 3) inhibiting the formation of volatile by‐products (borazine and ammonia).^[^ ^12^ ^]^ In aprotic solvents such as methanol, hydrogen release from AB can also be attained using homogeneous or heterogeneous catalysts. However, the amount of hydrogen released per mole of AB is small and the hydrogen release rate is lower than that of other hydrogen production processes.^[^ ^13^ ^]^ Methanol and other hydrogen production processes are also toxic. Hydrolytic dehydrogenation is the most widely studied alternative method, because it can be carried out under mild conditions.^[^ ^14^ ^]^ Furthermore, hydrolytic dehydrogenation can be conducted at room temperature, and the reaction rate can be artificially controlled. In this process, water is not only used as the reaction medium but also as a pure hydrogen fuel source. According to Equation (1), in the presence of a metal catalyst, 3 moles of hydrogen can be released per mole of AB in hydorlysis.^[^ ^15^ ^]^

(1) $${\mathrm{NH}}_{3}{\mathrm{BH}}_{3}+2H_{2}O\left(l\right)\to {\mathrm{NH}}_{4}{\mathrm{BO}}_{2}+3H_{2}\left(g\right)$$

### 1.3 Standardized Description of Catalytic Performance Tests

#### 1.3.1 Hydrolysis of AB in Reactors

**Figure 2:**
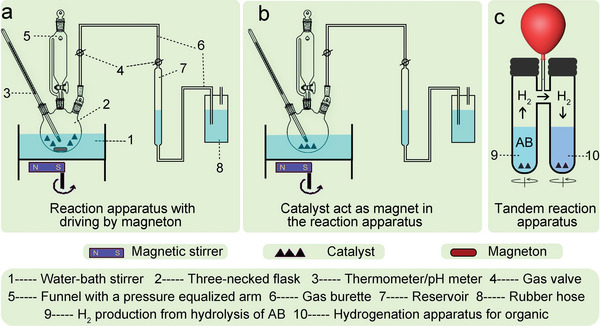
Experimental device diagram. a) Reaction apparatus with driving by magneton. b) Catalyst acts as a magnet in the reaction apparatus. c) Tandem reaction apparatus for hydrogenation with AB.

Reactors can be classified into two main types, as illustrated in **Figure** **2**: those that rely on magnetons to drive the reaction apparatus,^[^ ^16^ ^]^ and those that use the catalyst to act as a magnet within the reaction apparatus.^[^ ^17^ ^]^ Typically, the catalyst is loaded into a round‐bottom flask, and a solution containing NH_3_BH_3_ rapidly injected using a gastight syringe. A gas burette filled with water is connected to the flask to collect the gas, and a constant‐temperature magnetic stirring apparatus is used to maintain a stirring speed of 500–1000 rpm. To ensure external diffusion of the solution, magnetons can be added to nonmagnetic catalysts (such as Ru, Pd, Rh, and Cu) (Figure 2a). In contract, for magnetic catalysts (such as Fe, Co, and Ni), the magnetic force of the catalyst is used to determine the diffusion (Figure 2b). The tandem reaction apparatus uses the H_2_ produced from the hydrolysis of AB directly in hydrogenation reactions, such as the hydrogenation of nitrobenzene, hydrogenation of phenylacetylene, and amination of benzonitrile (Figure 2c),^[^ ^18^ ^]^ thereby increasing the application of liquid hydrogen storage materials.^[^ ^19^ ^]^

#### 1.3.2 Standard Procedure for Dehydrogenation of AB

The standard procedure for AB dehydrogenation involves weighing the catalyst into a round bottom flask, adding water to ultrasonically disperse it consistently, and placing the flask in a water bath at a controlled temperature and stirring speed. The prepared aqueous AB solution is then added to a constant‐pressure separation funnel. Petroleum gel is applied to the relevant interface to ensure airtightness and gas generation quantitatively evolved through drainage gas collection. During the hydrogen production test, the separatory funnel valve is opened, causing the AB aqueous solution to generate gas under the influence of the catalyst. The gas produced is manually recorded over time, and the corresponding turnover frequency (TOF) values are calculated using a formula and plotted on a graph. The same procedure is followed for the intermittent cycle test.

#### 1.3.3 Error Control

For repeatability and reliability of the data, each sample is prepared and tested at least three times, and error bars are added to the data processing. The error margin of error should not exceed 5%. All tests are conducted in a natural environment at room temperature.

#### 1.3.4 Calculation of TON and TOF

The modified gas volume calculates as:

(2) $$\;V_{\mathrm{modified}}=V_{\mathrm{recorded}}\;-V_{\mathrm{blank}}$$

The standard molar gas volume of H_2_ L mol^‐1^, *V*m_(H2)_ = 24.49 L, when the temperature is 298 K.

At room temperature of 298 K, amount of substance for *n*(H_2_) is calculated as

(3) $$n\left(H_{2}\right)\;=\frac{V_{\mathrm{modified}}-V_{\mathrm{blank}}}{Vm_{H2}}\;$$

Turnover number (TON): Amount of product *n*(H_2_)/amount of catalyst *n*_cat_ produced per unit time (or period of time).

(4) $$\mathrm{TON}\;=\frac{n\left(H_{2}\right)}{n_{\mathrm{cat}}}\;$$

Turnover frequency (TOF): Number of reactions per unit time and per unit active site for a given temperature, pressure, reactant ratio, and degree of reaction.

(5) $$\mathrm{TOF}\;=\frac{d\left(n_{H_{2}}\right)}{n_{\mathrm{cat}}\mathrm{dt}}\;$$

The variable “*n*_cat_” represents the quantity of moles of the catalytic active center. In the case of catalysts with metal loads lower than 5%, the precise amount of metal present can be determined by conducting an inductively coupled plasma (ICP) test. However, for catalysts with a theoretical metal load exceeding 5%, the exact metal load can be determined through temperature programmed desorption. Calculation of metal load is not recommended to rely on semiquantitative characterization methods such as X‐ray photoelectron spectroscopy (XPS) or energy disperse spectroscopy (EDS).

#### 1.3.5 Analysis Data of Hydrogen Production

There are three common methods used to determine the reaction order in the catalytic hydrolysis of AB: the differential method, the integral method, and the unit method of velocity constant. When using the differential method, the change in reaction conversion rate should not exceed 5%. The variation in calculation results reported in different literature is mainly due to inconsistent selection of conversion rate. The primary concern is determining the reaction order with respect to the catalyst, rather than with respect to AB (typically first order for the catalyst and zero order for the reactant).

#### 1.3.6 The Effect of Sodium Hydroxide

In previous research, sodium hydroxide and potassium hydroxide were thought to act as cocatalysts for AB dehydrogenation.^[^ ^17^ ^]^ A reasonable explanation is that OH^‐^ ions attack B and promote the cleavage of B–H. However, the corrosion of metaborate BO_2_^−^, Na^+^, K produced rapidly in strong alkali environment on the catalyst surface is not negligible. BO_2_^−^, Na^+^, and K can cover and damage the active site, resulting in a short‐term reduction in catalyst durability.^[^ ^20^ ^]^ Therefore, the concentration of the added strong base must be considered when calculating the catalytic activity of AB dehydrogenation systems using sodium hydroxide or potassium hydroxide.

#### 1.3.7 Theoretical Principles of Catalyst Design

Arrhenius equation:

(6) $$k\;=\;A\cdot \exp\left[\left(-\frac{E_{a}}{RT}\right)\right]$$

*k* is the rate constant, *R* is the molar gas constant, *T* is the thermodynamic temperature, *E*_a_ is the apparent activation energy and *A* is the finger front factor (also known as the frequency factor).

Apparent activation energy:

(7) $$\;E_{a}=\Delta_{r}\;H_{m}^{\#}+\Delta nRT\;=\Delta_{r}\;G_{m}^{\#}+T\Delta_{r}S_{m}^{\#}$$

Introducing the transition state view into the Arrhenius equation leads to the following formula:

(8) $$k\;=\;\left(k_{B}T/h\right)\cdot \exp\left(\Delta_{r}S_{m}\#/R\right)\cdot \exp\left(-\Delta_{r}H_{m}\#/RT\right)$$

*K*_B_ is Boltzmann constant, *h* is Planck constant, *R* is gas constant.

Unique process to increase the catalytic rate constant, *k*, according to the Arrhenius formula (Equation 6), is to raise the thermodynamic temperature, *T*. However, investigating the effect of temperature on activity is not usually the focus of catalytic science research. Instead, the most common approach is to decrease the activation energy, *E*_a_, while maintaining a constant temperature. Catalysts such as RuNi/TiO_2_ (914 min^−1^, 28.1 KJ mol^‐1^),^[^ ^21^ ^]^ Ni_2_Pt@ZIF‐8 (2222 min^−1^, 23.3 KJ mol^‐1^),^[^ ^18^ ^]^ and CuCo_2_O_4_ (104 min^−1^, 22.6 KJ mol^‐1^),^[^ ^22^ ^]^ have all shown improved catalytic activity as a result of reduced activation energy. On the other hand, catalysts such as Pt_0.1%_Co_3%_/TiO_2_ (2250.0 min^−1^, 63.8 KJ mol^‐1^)^[^ ^23^ ^]^ and CoNiP@GO (151 min^−1^, 44.1 KJ mol^‐1^),^[^ ^24^ ^]^ have high activation energies and high catalytic activity. Combining Equations (6) and (7) yields Equation (8). In addition to decreasing the activation energy (∆*E*_a_, or the activation enthalpy, ∆rH_m_), catalytic activity can also be increased by increasing the orientation factor (∆r*S*_m_, or the activation entropy). The value of ∆r*S*_m_ depends on the arrangement and combination of active sites, especially in the case of multisite catalysts. The design of bimolecular active sites can therefore increase the rate constant *k* and improve AB hydrolysis activity by increasing ∆r*S*_m_.

### 1.4 Performance of Catalysts

**Figure 3:**
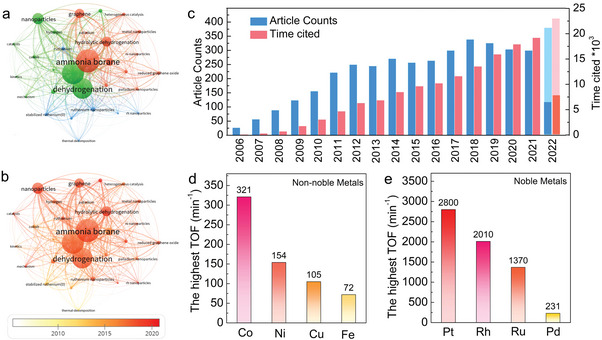
a) Network visualization of AB related keywords, b) and AB visualization hotspot research in the last 20 years c) Number of articles and citations on AB hydrogen production from 2002 to 2022. d) The highest TOF of noble metals Co, Ni, Cu, and Fe. e) The highest TOF of non‐noble metals Pt, Rh, Ru, and Pd.

As a representative of chemical hydrogen storage, AB has been extensively studied for its high‐density hydrogen release properties in liquid systems. With the growing interest in hydrogen energy, research on AB dehydrogenation has gradually gained traction since 2006 (**Figure** **3**a–c). From 2012 to the present day, the annual number of relevant papers has remained steady at around 300, with an upward trend. These papers have been cited approximately 15 000 times per year. A variety of transition metals are employed for AB hydrolysis, with noble metals such as Pt,^[^ ^25^ ^]^ Rh,^[^ ^26^ ^]^ Ru,^[^ ^27^ ^]^ Pd,^[^ ^28^ ^]^ and non‐noble metals such as Co,^[^ ^29^ ^]^ Ni,^[^ ^30^ ^]^ Cu,^[^ ^22^, ^31^ ^]^ and Fe^[^ ^32^ ^]^ being the most commonly used.

**Tables** **1** and **2** summarize the TOF, activation energy (*E*_a_), particle size (nm), durability, and other relevant information on noble and non‐noble metal catalysts over the past decade. As shown in Figure 3d, among the non‐noble metal catalysts, the catalyst 1.5Co1.5Ni/molybdenum carbide (MoC) synthesized by Ma et al.^[^ ^33^ ^]^ has the highest TOF of 321 min^−1^ among the non‐noble metal catalysts. Among all Ni‐based catalysts, the Ni_0.7_Co_1.3_P/graphene oxide (GO) catalyst designed by Chen et al.^[^ ^34^ ^]^ had the highest activity of 154 min^−1^. The highest catalytic activities for the Cu‐based^[^ ^35^ ^]^ and Fe‐based^[^ ^36^ ^]^ catalysts were 105 min^−1^ and 72 min^−1^, respectively. As shown in Figure 3e, the Rh_0_/CeO_2_ catalyst reported by the Özkar group^[^ ^37^ ^]^ exhibited the highest catalytic activity among the Rh catalysts, and the highest activity among all the catalysts, with a TOF value of up to 2010 min^−1^. The Pt_2_/graphene catalyst reported by the Lu group^[^ ^38^ ^]^ has the highest activity (2800 min^−1^) among platinum‐based catalysts. The Ru‐based catalyst supported on TiO_2_ reported by Li et al.^[^ ^39^ ^]^ has the highest Ru activity. At present, the catalytic activity of palladium metal is relatively low, and the highest activity was demonstrated by the Pd/alk‐Ti_3_C_2_ catalyst reported by Liu et al.^[^ ^40^ ^]^ with a TOF of 231 min^−1^.

**Table 1:** Performance indicators for non‐precious metal catalysts from 2009 to 2022

Years	Catalyst	TOF [min^−1^]	*E*_a_ [KJ mol^‐1^]	Size [nm]	Durability	*T* [K]	Refs.
2009	Co–B	8.2	44.0	–	–	298	[44]
2010	Ni NPs	8.8	28.0	3.0	–	298	[45]
2010	Co nanoparticles	2.9	–	5.0	–	298	[46]
2011	Fe_0.3_Co_0.7_	23.6	16.3	20.0	–	293	[47]
2011	PEG stabilized iron(0)	6.4	37.0	6.3	–	298	[48]
2011	Ni/AB = 0.019	8.4	–	4.5	–	298	[49]
2012	Surfactant‐free Ni NPs	30.7	–	5.0	–	298	[50]
2012	Co–P–B	21.0	38.8	–	–	298	[12]
2013	Ni@meso‐SiO_2_	18.5	29.0	–	–	298	[51]
2013	Cu@Co/rGO	8.7	51.3	–	–	298	[52]
2014	MWCNTs	–	50.4	–	–	298	[53]
2014	PEI‐GO/Co	39.9	28.2	2.6	–	298	[54]
2014	50 W Co	7.0	60.0	200.0	–	298	[55]
2014	Cu_0.2_Ni_0.8_/MCM‐41	10.7	38.0	–	–	298	[56]
2014	Ni/C‐3	2.2	31.6	10.0	–	298	[57]
2014	CuCo/MIL‐101	19.6	–	2.0	–	298	[58]
2015	Ag/Ni	–	25.0	2.5	–	298	[59]
2015	PVP‐stabilized nickel (0)	12.1	65.0	3.0	–	298	[60]
2015	Ni‐CNT	23.5	–	–	–	298	[61]
2015	Flower‐like Cu	2.41	34.2	–	–	298	[62]
2016	CNG‐I	9.4	35.4	20.0	–	298	[63]
2016	CCGC	4.5	47.1	50.0	–	298	[64]
2016	Co@CoOx@N‐CG	14.7	36.6	50.0	–	298	[17]
2016	NiCo_2_O_4_/Ti	50.1	17.5	–	–	308	[65]
2016	Ni_2_P NA/NF	42.3	44.0	–	–	298	[66]
2016	Co@N‐C‐700	5.6	31.0	9.0	–	298	[67]
2016	CoBSSR	6.9	22.8	–	–	298	[68]
2016	Ni_0.9_Mo_0.1_/graphene	66.7	21.8	3.4	–	298	[69]
2016	Ni/MIL‐101	54.0	–	–	–	298	[70]
2016	Cu_0.8_Ni_0.2_	–	40.5	–	–	298	[71]
2016	Co/CTF	42.3	–	4.0	–	298	[72]
2017	Co‐Co_3_O_4_@C‐II	14.1	37.1	15.0	100%/6	298	[73]
2017	Ni_2‐_ *_x_* Co*_x_* P	58.4	43.2	6.5	–	298	[34]
2017	CuCo/MIL‐101‐1‐U	51.7	30.5	4.5‐8.5	–	298	[74]
2017	CuCo_2_O_4_	44.0	23.6	–	95%/8	298	[75]
2017	Ni_0_/PDA‐CoFe_2_O_4_	7.6	50.8	12.3	100%/10	298	[76]
2017	NiNPs/ZIF‐8	85.7	42.7	2.7	100%/4	298	[77]
2017	Ni_0.8_W_0.2_	25	47.3	–	–	298	[78]
2017	Co/g‐C_3_N_4_‐1	55.6	–	–	100%/10	298	[79]
2017	Co/C_3_N_4_‐540	83.3	–	–	–	298	[80]
2017	Co/NPCNW	7.3	25.4	4.0	94.6%/10	298	[81]
2017	Cu_2_Ni_1_@MIL‐101	20.9	32.2	1.5‐2.0	75%/5	298	[82]
2018	8.9 Ni NP	154.2	66.6	8.9	90%/4	298	[83]
2018	Co‐W‐B	8.8	32.2	67.3	78.4%/5	298	[84]
2018	Co_0.8_Cu_0.2_MoO_4_	55.0	–	–	–	298	[85]
2018	Cu(OH)_2_/Fe(OH)_3_	135.6	42.6	–	–	298	[86]
2018	CuO‐NiO/C	62.5	59.4	–	–	298	[87]
2018	CoO*_x_*‐PG	3.9	51.3	20.0	90%/5	298	[88]
2018	Ni‐CeO*_x_*/graphene	68.2	28.9	10.0	–	298	[89]
2018	Cu_0.72_Co_0.18_Mo_0.1_	46.0	45.0	5.5–6.0	–	298	[90]
2018	Co@NMC‐800‐0.5	–	41.6	12.5	–	298	[91]
2018	Cu_0.5_Co_0.5_O‐rGO	81.7	45.3	–	88.3%/5	298	[92]
2019	Co‐CoOx@NCS‐II	14.6	46.4	7.2	–	298	[20]
2019	Co@C‐N@SiO_2_‐800	8.4	36.1	–	98%/5	298	[93]
2019	Cu/Co(OH)_2_	61.6	37.6	5.0	–	298	[94]
2019	Co_0.67_Ni_0.33_/Al_2_O_3_	34.5	32.4	–	–	298	[95]
2019	Ni/g‐C_3_N_4_	18.7	36.0	3.2	75%/4	298	[96]
2019	NiCu/47‐SiO_2_	25.3	34.2	47.0–485.0	90%/5	298	[97]
2019	Co(5)@KD	20.1	32.6	3.1–4.0	–	303	[98]
2019	Co*_x_* Cu_1−_ *_x_* Co_2_O_4_@Co*_y_* Cu_1−_ *_y_* Co_2_O_4_	81.8	25.0	3000	–	298	[99]
2019	Co_3_O_4_/CuMoO_4_	129.1	23.2	–	–	298	[100]
2019	Ni_0.66_Co_0.19_P_0.15_/ OPC‐300	95.2	–	1.2	85%/5	298	[101]
2019	Ni_0.5_Co_0.5_O‐NCN	76.1	43.2	5.0	83.2%/6	298	[102]
2019	Co/V_2_O_5_‐300	120.4	–	–	–	298	[103]
2019	PF5‐I	12.3	30.8	–	–	298	[104]
2019	Ni‐Fe‐P/Ni	1.8	42.0	–	–	298	[105]
2019	Cu_0.4_Co_0.6_/BNNFs	8.4	21.8	7.2	55%/5	298	[106]
2019	30% Co/HPC900	2.9	32.8	15.0‐30.0	90%/12	298	[107]
2020	Ni_1.2_Fe_0.8_@CN‐G	23.3	36.8	4.0	–	298	[108]
2020	Co‐CN‐O‐100	14.4	39.4	4.2	–	298	[109]
2020	Co‐CoO*_x_* @GO‐II	15.3	62.3	–	–	298	[110]
2020	COTC‐II	15.6	38.5	20.0	–	298	[111]
2020	10%‐CoNi/HPC‐400	27.2	34.0	7.8	–	298	[112]
2020	Cu_2_O‐CoO	34.1	34.1	–	–	298	[113]
2020	Co_3_ *_x_* Cu_3‐_ *_x_* (PO_4_)_2_	72.6	29.0	–	–	298	[114]
2020	Cu_0.3_@Cu_0.7_CoO*_x_* @GO	44.6	35.4	5.0	–	298	[115]
2020	CuNi‐MOFs	40.9	29.0	–	–	298	[116]
2020	CuO‐NiO/Co_3_O_4_	79.1	23.7	–	–	298	[117]
2020	SCo_0.43_Cu_0.57_	5.7	31.1	–	71.8%/5	298	[118]
2020	Cu_0.36_Ni_0.64_‐T700	21.9	27.4	8.0	49.4%/5	298	[119]
2020	Ni_0.23_Co_0.19_P_0.58_@NHPC900	125.2	–	–	–	298	[120]
2020	NiCoP/TiO_2_	–	52.8	–	–	298	[30]
2020	Co‐Mo‐B/NF	15.9	43.6	60.0‐70.0	–	298	[121]
2020	Co‐Co_3_O_4_/CDs	18.0	40.0	–	–	298	[122]
2020	Co/Cu‐190	164.8	–	190.0	–	298	[123]
2020	CuCo(O)@CN	12.4	33.8	–	64.7%/5	298	[124]
2020	NiCu/CNS	30.6	–	–	–	298	[125]
2020	CuPd_0.01_@ZIF‐67@ZIF‐8	30.2	38.8	2.8	–	298	[126]
2021	Ni/FeNiO*_x_*‐25	72.3	39.2	–	–	303	[36]
2021	Cu_0.5_@Co_0.5_‐MOF/5	130.0	26.5	5.5	–	298	[31]
2021	NiMn‐decorated CNFs	58.2	38.9	60.0	100%/5	298	[127]
2021	Co_40_Cu_60_@ S16LC‐20	16.4	38.1	8.0	–	298	[128]
2021	cZIF‐67_µm	13.5	–	–	–	298	[15]
2021	Co_3_O_4_‐CuCoO_2_	65.0	20.5	–	–	298	[129]
2021	CuCo_2_O_4_	104.0	22.6	–	–	298	[22]
2021	Ni_0.25_Co_0.75_O/Cu@CuO	11.5	–	–	–	298	[130]
2021	CuMoO_4_‐CoMoO_4_	104.7	38.4	–	–	298	[35]
2021	1.5Co1.5Ni/*α*‐MoC	321.1	–	–	–	298	[33]
2021	Co@Co_2_Mo_3_O_8_	17.3	51.8	–	–	298	[29b]
2021	Ni/NiO@MoO*_x_*‐50H	86.3	27.1	–	–	298	[131]
2021	W_18_O_49_SU	53.1	–	–	–	298	[132]
2021	hcp‐CuNi/C	22.6	29.9	–	–	298	[133]
2021	rGO/CoNi‐N	126	32.8	8.0	–	298	[134]
2021	CuO‐Co_3_O_4_	33.4	39.6	–	–	298	[135]
2021	Zr‐Ni‐B	0.9	79.7	–	–	298	[136]
2021	10Ni_3_0Mo*_x_* C/*γ*‐Al_2_O_3_	75.1	33.1	–	–	298	[137]
2021	Co/CeVO_4_@PDA37	115.4	–	–	–	298	[138]
2021	Co_0.7_Ni_0.3_	35.3	23.6	–	–	298	[139]
2021	GR	0.1	–	100.0	–	298	[32]
2021	CuFe LDOs	–	35.9	–	–	298	[140]
2021	ZIF‐67@Co	112.3	–	–	92%/5	298	[141]
2021	CoFe_2_O_4_	4.1	47.9	–	–	303	[142]
2021	CoP‐CoO/NCDs	89.6	41	–	–	298	[143]
2021	Ni_0.13_Co_0.8_7P	47.5	41.8	–	–	298	[144]
2021	NiCo‐NC	35.2	43.6	–	–	298	[145]
2021	G‐Cu/_0.5_‐Ni‐NiO*_x_*	17.7	–	10.0	–	298	[146]
2021	CoNiP/GO	134.6	44.1	–	84.6%/5	298	[24]
2022	Co‐NC/NF_600_	3.0	64.0			298	[147]
2022	Co_3_BCoP/h‐BN	37.0	51.8			298	[148]
2022	Co_4_N‐Co_3_O_4_@C	79.0	28.8		93%/5	298	[149]
2023	Co‐CoP‐NC/NF‐2	10.0	30.6	8.8	–	298	[150]
2023	O‐(CoP/Co_2_P)@SC	35.0	57.9	200.0	–	298	[151]
2023	10Ni30Mo_2_C/CNTs	71.0	41.7	–	–	298	[152]
2023	CoCu‐BCs/GO	72.4	47.8	–	60%/5	298	[153]

**Table 2:** Performance indicators for precious metal catalysts from 2009 to 2022

Years	Catalyst	TOF [min^−1^]	*E*_a_ [KJ mol^‐1^]	Size [nm]	Durability	*T* [K]	Refs.
2011	Co_0.32_@Pt_0.68_/C	12.9	41.5	2.5–4.0	–	298	[154]
2012	2 wt% Pt@MIL‐101	26.4	–	–	–	298	[155]
2012	1.0‐Ru@Al_2_O_3_	83.3	58	2.2‐2.9	–	298	[156]
2012	Ni@Ru core@shell	–	39.1	20.0	–	298	[157]
2012	RGO/Pd	6.3	51	1.8	–	298	[158]
2013	Ru(0)@HAp	137.0	59	4.7	92%/5	298	[27c]
2013	NiCo‐Pt	15.0	45.7	–	83%/5	298	[159]
2013	Ag@Co/graphene	102.4	20.0	–	49.7%/5	298	[160]
2013	Ag_0.1_@Co_0.45_Ni_0.45_/graphene	–	36.2	–	51%/5	298	[161]
2013	AuNi@MIL‐101	66.2	–	–	–	298	[162]
2013	Metastable RuNPs	21.8	27.5	2.2	–	298	[163]
2014	Pd(0)/SiO_2_‐CoFe_2_O_4_	254.0	52.0	–	–	298	[164]
2014	Pt/CNTs‐O‐HT	468.0	–	–	–	298	[165]
2014	Pt/CNT	400.0	38.0	1.8	–	298	[166]
2014	AuCo@MIL‐101	23.5	–	–	–	298	[167]
2014	PAN/Ag/Pd	377.2	–	5.0–10	–	298	[168]
2014	Pt:Ni = 4:1	638.0	–	3.0–5.0	–	298	[169]
2014	Ru@SiO_2_	200.0	38.2	25.0	–	298	[170]
2014	Pd‐Pt @PVP NPs	125.0	51.7	4.2	–	298	[171]
2014	Pd‐Rh@PVP NPs	1333.0	46.1	2.5	–	298	[172]
2015	Au‐Co@CN	48.3	–	–	–	298	[173]
2015	Ru@SBA‐15 NCs	316.0	34.8	3.0	–	298	[174]
2015	Ru_1_Co_9_/Ti_3_C_2_X_2_	896.0	31.1	2.0‐3.0	–	298	[175]
2015	Rh/CNTs	706.0	32.0		–	298	[176]
2015	Rh_0_/CeO_2_	2010.0	43.0	2.5‐3.5	67%/5	298	[37]
2016	RuNi/TiO_2_	914.0	28.1	2.3	–	298	[21]
2016	Pd‐Ru core–frame	792.0	–	2.0	–	298	[177]
2017	Pt_0.5_Ru_0.5_/CNT	745	36.0	2.0‐2.5	–	298	[178]
2017	Pd0/PDA‐CoFe_2_O_4_	175.0	65.0	1.4	–	298	[179]
2017	Rh/P(triaz)‐free	260.0	–	1.0	–	298	[180]
2017	Pt1/graphene‐R	2800.0	–	1.8	–	300	[38]
2017	Pt_3_Ni_7_O‐NGO	709.6	–	–	76%/9	298	[181]
2017	SNP‐Pt_65_Ti_35_	51.6	39.4	–	63%/5	298	[182]
2017	1/1000Pt+Ni/CNT	12 000.0	–	SACs	–	298	[43]
2018	Ni_2_Pt@ZiF‐8	2222.0	23.3	–	–	293	[18]
2018	Pd‐Au/IOP	25.0	52.5	1.5	–	298	[28a]
2018	situ‐Rh/C	762.6	40.9	0.7‐0.9	61.2%/8	298	[183]
2018	Rh(0)/nanoCeO_2_	144.0	–	3.9	–	298	[184]
2018	Ru0/ZrO_2_	173.0	58.0	1.4	67%/5	298	[185]
2018	Pt_1_Co_1_/1	303.0	28.8	2.0	–	293	[186]
2018	Ru/C‐300	643.0	38.7	2.0	50%/8	298	[187]
2018	Pd_74_Ni_26_/MCN	246.8	54.1	2.4	–	298	[188]
2018	NiPd‐NG‐Si	81.5	18.8	–	–	298	[189]
2018	PtCo_20_/CNTs	675.1	44.7	2.0–3.0	–	298	[190]
2018	HPRhS	296.1	–	2.3–2.8	–	298	[191]
2018	Rh/AC	188.0	39.9	2.9	100%/5	298	[192]
2019	Pt1/Co_3_O_4_	1220.0	37.4	SACs	–	298	[193]
2019	Rh/WO_3‐_ *_x_*	805.0	50.5	1.7	29.7%/5	298	[194]
2019	PtCoNi‐BOFs	1490.0	15.8	–	–	298	[25b]
2019	Ru/FAU (Si/Al = 30)	627.0	60.7	1.8–2.1	–	298	[195]
2019	Rh@S‐1‐H	699.0	75.5	SACs	–	298	[196]
2019	Rh0/CoFe_2_O_4_	720.0	66	2.0	100%/5	298	[197]
2019	RuCo@HCSs	784.0	27.4	10.0	–	298	[27b]
2019	CoRu_0.25_@N‐C	457.8	32.5	–	–	298	[198]
2020	mpg‐CN/Pt	274.2	–	4.0–6.0	78%/10	298	[25c]
2020	Pt/CNT‐5W	710.1	–	1.8–2.1	68%/5	298	[199]
2020	Rh/o‐Ti_3_C_2_T*_x_*	2021.0	18.7	2.6	53%/5	298	[200]
2020	Ru/PC	744.0	39.1	5.0–7.0	53.9%/5	298	[201]
2020	AB@Pd/HNTs	–	46.0	2.0	–	333	[202]
2020	Pt_76_Au_12_Co_12_	450.0	18.5	–	56%/5	298	[203]
2020	STA‐Pt/CNT	517.0	–	1.8	–	298	[204]
2020	Ru/g‐C_3_N_4_	122.2	35.6	–	–	298	[205]
2020	Ru_1‐_ *_x_* Pd*_x_*/g‐C_3_N_4_	984.0	24.2	2.3–2.8	–	298	[206]
2020	Ru_0.5_Ni_0.5_/p‐g‐C_3_N_4_	840.0	14.1	–	–	298	[27a]
2020	Fe_3_O_4_@SiO_2_:g‐C_3_N_4_	33.7	31.4	7.6–9.8	–	298	[207]
2020	C‐AuPd	160.0	22.4	–	–	298	[208]
2020	CoRh@PVP	154.0	42.7	3.4–3.8	75%/5	298	[209]
2020	Ru_0.5_0Ni_0.5_0‐SiO_2_	33.6	–	–	–	323	[210]
2020	Al_2_O_3_‐PtNi	426.8	45.7	3.0–5.0	–	298	[211]
2020	Ag@Pd Core–shell	103.7	31.8	–	92%/5	298	[212]
2020	porous Pt_25_Pd_75_	69.8	29.1	–	–	298	[213]
2020	Ru_2_Fe_1_/N‐C	424.0	33.7	2.0–4.0	–	298	[214]
2020	CuPd_0.01_@ZIF‐67@ZIF‐8	30.2	38.8	3.0	–	298	[126]
2021	Ru_0.6_Cu_0.4_/TiO_2_@C‐N	626.0	26.3	5.5	–	298	[215]
2021	Ni_1_Ru1/TCN	1046.2	24.3	–	–	298	[216]
2021	Pd/alk‐Ti_3_C_2_	231.0	21.2	4.9	–	298	[40]
2021	Pt_0_/Co_3_O_4_	4366.0	71.0	3.5–4.0	100%/10	298	[25a]
2021	Ru_0_/CeFe	93.0	–	–	100%/5	298	[217]
2021	Rh_0_/WO_3_	749.0	39.0	4.8–6.0	–	298	[218]
2021	Rh/Al_2_O_3_‐CTAB‐400	246.8	47.9	–	–	298	[219]
2021	Rh/UiO‐66‐NH_2_	876.7	22.3	3.0–4.0	62.5%/5	298	[220]
2021	Pt‐PVP/SiO_2_(M)	371.0	46.2	2.0–3.0	–	298	[221]
2021	Rh_2_P@HMC	939.0	37.9	–	–	298	[222]
2021	Ru_1_Ni_1.90_/NCS	1017.0	26.6	2.0–3.0	64%/5	298	[223]
2021	Ru_0.1_Co_0.9_/g‐C_3_N_4_	1260.0	22.5	1.6	–	298	[224]
2021	Ru/p‐C	744.7	28.8	4.1	–	303	[225]
2021	CoRu_0.5_/CQDs	814.7	39.3	–	–	298	[226]
2021	Ru/h‐BN	1177.5	24.1	–	–	303	[227]
2020	Pt^0^/CoFe_2_O_4_	3628.0	65.0	2.3	–	298	[42]
2021	Rh_1_Cu_2_/CoOx	670.9	28.9	4.4–7.6	–	298	[228]
2021	Rh/Ni@NiN‐C	782.2	25.9	4.0	–	298	[26b]
2021	Rh/OPNC	433.0	–	2.0–3.0	–	298	[229]
2021	Pd_1_@Co_7.5_@P25/rGO	127.6	39.1	–	63.6%/5	298	[230]
2021	PtNiO*_x_* TVO	618.0	52.5	–	–	298	[231]
2021	1.5‐PdTVO	240.0	34.6	–	–	298	[232]
2021	Rh/Ni@CN	351.0	33.5	–	50%/5	298	[233]
2021	Rh/SP‐S‐1	430.0	–	–	–	298	[234]
2021	Ru_1_Ni_7.5_/g‐C_3_N_4_	901.0	28.5	3.7	46%/5	298	[235]
2021	RP/CN	134.0	67.7	–	–	298	[236]
2021	RuP@NHMCs	1774.0	36.3	–	–	298	[41]
2021	RhNi@NHMCs	1294.0	18.6	–	–		[237]
2021	Rh@TiO_2_	334.1	–	3.0–5.0	–	298	[238]
2021	Cu*_x_* Co_1−_ *_x_* Pt*_y_* O/RGO	854.0	–	2.0–4.0	80%/5	298	[239]
2021	Ru@f‐CNTs	764.0	36.7	1.5–3.0	–	298	[240]
2022	Ru@POF‐NC600	727.0	39.4	3.2	–		[241]
2022	1.5‐RTVO‐4	1370.0	37.0	1.0–2.0	–	298	[39]
2022	PtNi@TiO_2_	1055.0	47.2	1.7–2.1	–	298	[242]
2023	PdCoO*_x_* P25	210.0	54.4	2.0	–	298	[243]
2023	Pt_0.1%_Co_3%/_TiO_2_	2250.0	63.8	1.3	–	298	[23]
2023	Ru/Ti3C_2−_ *_x_* N*_x_*	1334.0	26.0	1.6	–	298	[244]

Furthermore, some ultrahigh activity catalysts have been reported. Liu et al. loaded RuP on NHMC and obtained the RuP@NHMC catalyst with a catalytic activity of 1774 min^−1^.^[^ ^41^ ^]^ The Saim Özkar group designed the Pt^0^/CoFe_2_O_4_ catalyst with a catalytic activity of 3628 min^−1^.^[^ ^42^ ^]^ Subsequently, Pt^0^/Co_3_O_4_ catalysts were designed with even higher activity at 4366 min^−1^.^[^ ^25^ ^a]^ The PtNi‐CNT catalyst synthesized by Chen et al. has a catalytic activity of 12 000 min^−1^, much higher than that of the current catalyst.^[^ ^43^ ^]^ This ultrahigh activity of PtNi‐CNT catalyst is mainly due to the large amount of non‐noble metals in the catalyst. The catalytic activity of non‐noble metals is not accounted for when the activity of noble metals is calculated.

## 2 In‐Depth Discussions on Supported Catalysts

**Figure 4:**
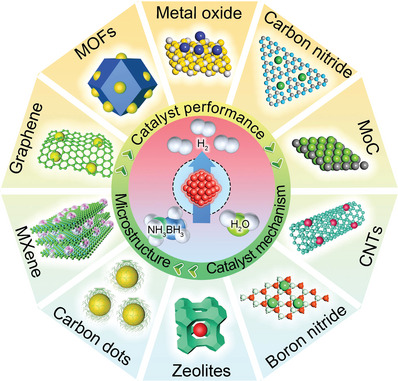
Schematic diagram of various supported catalysts.

In this chapter, the preparation process and structural characteristics of various supported catalysts are briefly introduced, including graphite, metal‐organic frameworks (MOF), metal oxide, carbon nitride (CN), MoC, carbon nanotubes (CNTs), boron nitrides (h‐BN), zeolites, carbon dots (CDs), metal carbide and nitride (MXene) (**Figure** **4**). The role of the support in influencing the active center and enhancing the catalytic activity during AB hydrolysis is analyzed accordingly.

### 2.1 Graphene‐Based Materials

Graphene, owing to its large specific surface area, act ideal support materials for the growth and dispersion of metal nanoparticles (NPs).^[^ ^115^, ^245^ ^]^ Several strategies, such as NaBH_4_ impregnation reduction,^[^ ^223^ ^]^ hydrogen impregnation reduction,^[^ ^230^ ^]^ atomic layer deposition (ALD),^[^ ^38^ ^]^ MOF precursor pyrolysis^[^ ^24^ ^]^ and metal hydroxide pyrolysis^[^ ^134^ ^]^ have been used to precisely and controllably load transition metal NPs on GO for AB hydrolysis. Various of AB hydrolysis catalysts supported on GO have been designed, and the active center and AB hydrolysis mechanism of GO‐based catalytic materials have been systematically analyzed.^[^ ^63^, ^223^, ^246^ ^]^ At present, GO is only used as a support to improve metal dispersion and anchor active sites. Whether the support GO is involved in the AB hydrolysis reaction as an active site has not been discussed in depth. The feasibility of Pt_2_/graphene as a commercial catalyst for AB hydrolysis can be evaluated as it currently has the highest catalytic performance.^[^ ^38^ ^]^

**Figure 5:**
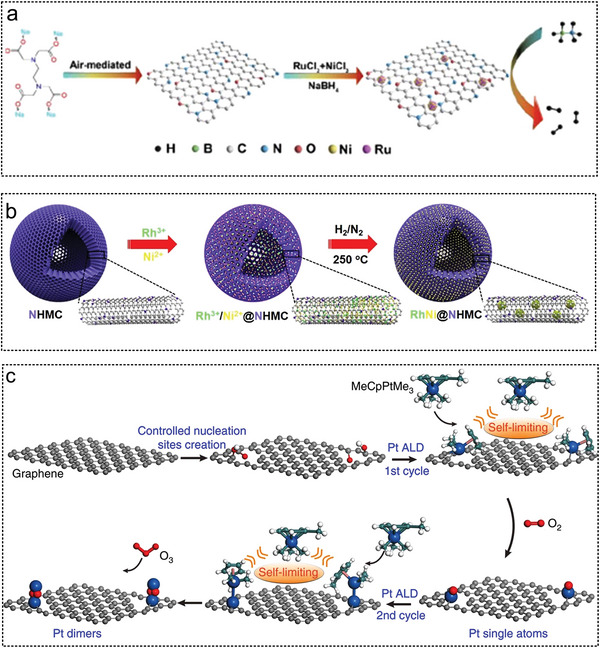
The GO as noble metal catalyst substrates for AB hydrolysis. a) Ru and Ni were reduced to graphene by NaBH_4_ to form RuNi‐rGO catalyst. Reproduced with permission.^[^ ^223^ ^]^ Copyright 2021, Elsevier. b) Illustration for structure evolution of RuNi@NHMC catalyst. Reproduced with permission.^[^ ^230^ ^]^ Copyright 2021, American Chemical Society. c) Single‐atom Pt and Pt dimers were synthesized on graphene by ALD. Reproduced with permission.^[^ ^38^ ^]^ Copyright 2017, Springer Nature.

Li et al. successfully prepared Ru particles with a diameter of approximately 1.3 nm on reduced graphene oxide (rGO) sheets via strong electrostatic interactions.^[^ ^247^ ^]^ The X‐ray diffraction (XRD) results showed a new diffraction peak, Ru (101), at 43.6°, proving the successful loading of Ru nanoclusters on the rGO sheet. Ru/rGO nanocomposites (NCs) have a low‐temperature gradient *E*_a_ 38.12 KJ mol^−1^. Fan et al. first synthesized graphite‐like NCS by direct carbonization of Na_4_EDTA in air. Then RuNi NPs (2–2.5 nm) were deposited on the NCS by an in situ impregnation‐reduction method (**Figure** **5**a). The catalytic performances of different RuNi ratios for AB hydrolysis were investigated. Ru_1_Ni_1.90_ /NCS had a TOF value of 824 min^−1^ and the lowest *E*_a_ of 26.5 KJ mol^−1^. On the surface of nitrogen‐doped hollow and mesoporous carbon (NHMCs), Liu et al. successfully supported two Ru‐based catalysts RuP@NHMCs^[^ ^41^ ^]^ and RhNi@NHMCs,^[^ ^237^ ^]^ with TOF of 1774 and 1294 min^−1^, respectively (Figure 5b). Further isotope tracing experiments confirmed that O—H dissociation was the rate‐determining step. Using methylamine borane as a reducing agent, Liu et al. synthesized Pd@Co@P transition metal phosphide NPs with a core–shell structure on GO by a one‐pot co‐reduction method at room temperature.^[^ ^230^ ^]^ Lu et al. deposited Pt_2_ dimers on GO via two consecutive atomic layer deposition ALD processes.^[^ ^38^ ^]^ Pt Single atom was formed on the GO surface after the first deposition of ALD at 150 °C (Figure 5c); after the second Pt ALD, single atom Pt adsorbed a certain number of Pt atoms to form Pt_2_ dimer. In the AB hydrolysis reaction, the adsorption energies of AB and H_2_ on the Pt_2_ dimer were lower than those on the Pt_1_ single atom or Pt NPs. The dimer Pt_2_/graphene catalyst showed significantly higher activity, approximately 17 times and 45 times higher than graphene‐supported Pt_1_ monatomic and Pt NPs, respectively.

**Figure 6:**
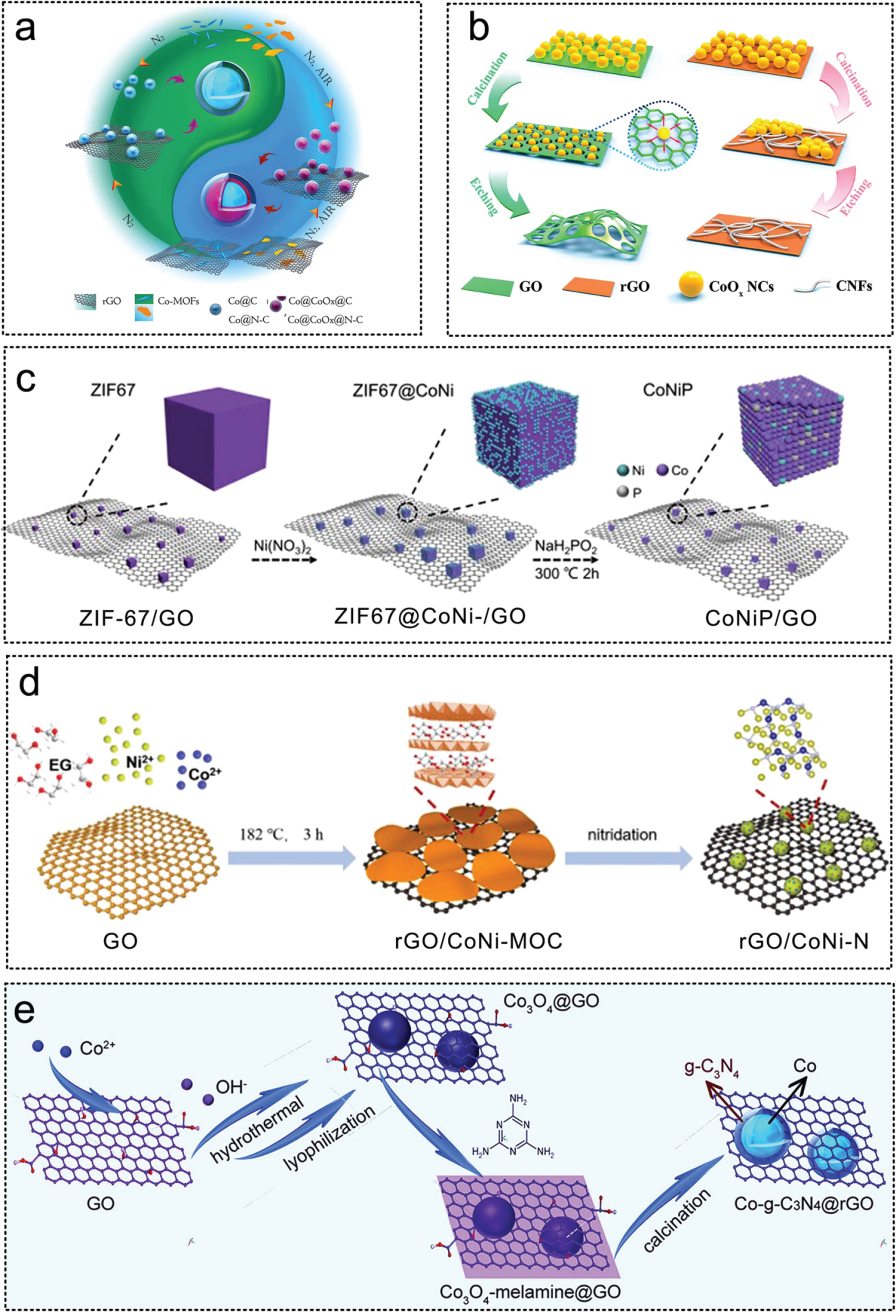
The GO as non‐noble metal catalyst substrates for AB hydrolysis. a) The schematic diagram of the formation of catalysts Co@CoO*_x_* @CG and Co@CoO*_x_* @N‐CG. Reproduced with permission.^[^ ^17^ ^]^ Copyright 2016, American Chemical Society. b) Illustration of the structure evolution CoO*_x_*‐PG, CoO*_x_*‐GCNFs, PG, and GCNFs. Reproduced with permission.^[^ ^88^ ^]^ Copyright 2018, American Chemical Society. c) ZIF‐67 /GO doping Ni to form ZIF‐67@CoNi‐/GO, and further phosphating to form CoNiP/GO. Reproduced with permission.^[^ ^24^ ^]^ Copyright 2022, Elsevier. d) Illustration for synthesis of metal nitrides catalysts supported on GO. Reproduced with permission.^[^ ^134^ ^]^ Copyright 2021, Elsevier. e) Core–shell catalyst with Co as core and g‐C_3_N_4_ as shell was supported on rGO. Reproduced with permission.^[^ ^63^ ^]^ Copyright 2016, American Chemical Society.

Li et al. synthesized multilayer Co‐based core–shell composites on N‐doped GO surface by adding polyvinylpyrrolidone and a Co‐based MOF precursor thermal decomposition method (**Figure** **6**a). The synergistic effect of the Co‐CoO*_x_* structure increased the hydrolysis activity of AB, with a hydrogen generation rate of 5560 mL min^−1^ g_Co_^−1^.^[^ ^17^ ^]^ Furthermore, Co_3_O_4_ nanocrystals (NCs) have been used as oxidants to modify and etch graphene sheets to improve the hydrogen production activity of porous graphene (Figure 6b).^[^ ^88^ ^]^ Chen et al. obtained a nitrogen‐doped cobalt‐based catalyst Co@N‐C, through the controlled pyrolysis of Co(salen), and systematically studied the effect of pyrolysis temperature on the catalyst structure and hydrolysis activity of AB. The optimum mesopores and micropores were obtained at 700 °C. The prepared catalyst has a catalytic activity of 5.6 min^‐1^ and *E*_a_ of 31.0 KJ mol^−1^, and can be used at least ten times.^[^ ^67^ ^]^ Zhong et al. successfully prepared a CoNiP/GO catalyst on a GO surface through the second metal Ni and phosphating experiment (Figure 6c). The X‐ray absorption spectroscopy (XAS) experimental results confirmed that the addition of a small amount of Ni (1.7 wt%) could significantly regulate the electronic structure of Co in the catalyst. The synergy between Co, Ni and P weakens the B—N bond energy in the AB molecule and improves catalytic performance.^[^ ^24^ ^]^ Li et al. synthesized defect‐rich Co—CoO*_x_* on graphene through controlled carbonization and oxidation with a hydrogen generation rate of 5813 mL min^−1^ g_Co_^−1^.^[^ ^110^ ^]^ A Co‐C‐rGO composite material (CCGC) with catalytic activity and superparamagnetic properties was synthesized using the inherent magnetism of ferromagnetic catalysts. CCGC acts as a nanodriver and simultaneously realizes the momentum transfer and hydrolysis of NaBH_4_ or H_3_NBH_3_ to produce hydrogen.^[^ ^64^ ^]^ Transition metal nitride catalysts supported on rGO were synthesized carbonizing of bimetallic CoNi organic complex precursors (Figure 6d).^[^ ^134^ ^]^ The unique morphology of rGO/CoNi‐N enables full exposure of the active catalytic sites and easy mass transfer, with a TOF of 126 min^−1^ and an *E*_a_ of 32.8 KJ mol^−1^. Li et al. designed a composite magnetic force driving catalyst with Co as the core, g‐C_3_N_4_ as the shell and rGO as the support, with a catalytic activity of 1253 mL min^−1^ g_Co_^−1^ (Figure 6e). Lu et al. synthesized molybdenum‐doped modified Ni NPs were synthesized on graphene sheets via a simple chemical reduction pathway, where the highest catalytic activity for hydrogen production from AB hydrolysis by a non‐precious metal catalyst was achieved with a turnover frequency of up to 66.7 min^−1^. Molybdenum doping induces a reduction in the size of the metal NPs, with the size of the Ni NPs being reduced from 10.4 nm to 3.4 nm.

### 2.2 Confinement Effect of MOFs

MOFs, consisting of metal ions and organic ligands, have advantages over traditional inorganic supports.^[^ ^248^ ^]^ The high porosity, large surface area, and chemical stability of MOF can limit and stabilize catalytically active metal NPs and fully exposed active sites.^[^ ^147^, ^249^ ^]^ At the same time, the interaction between MOF and the active metal can promote the adsorption of borohydrides such as AB on the catalyst surface.^[^ ^250^ ^]^ A variety of MOF materials and MOF derivatives, such as MIL‐101,^[^ ^251^ ^]^ ZIF‐67,^[^ ^252^ ^]^ and ZIF‐8,^[^ ^253^ ^]^ are commonly used as supports for AB water interpretation of hydrogen, including noble and non‐noble metals. To further improve the catalytic activity, the next step is to study multi‐metal‐based MOF materials and analyze the structure‐activity relationship between the multi‐metal and MOF site.

**Figure 7:**
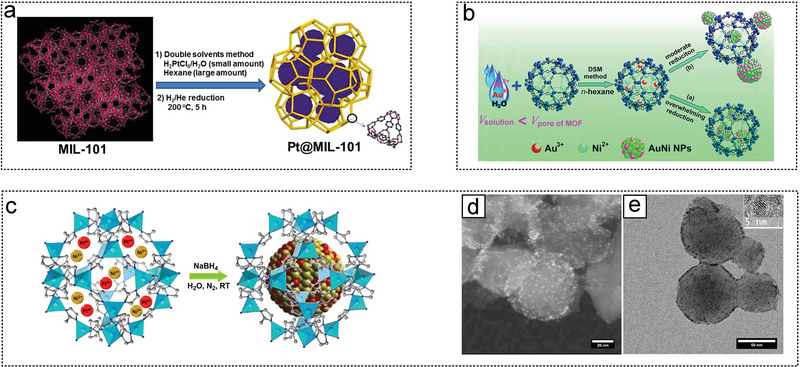
The MOF as noble metal catalyst substrates for AB hydrolysis. a) Pd NPs were immobilized in MIL‐101 synthesis strategy by a reduction strategy using DSM method. Reproduced with permission.^[^ ^155^ ^]^ Copyright 2012, American Chemical Society. b) Au–Ni alloy NPs were immobilized in MIL‐101 synthesis strategy by a reduction strategy using the double solvents method (DSM). Reproduced with permission.^[^ ^162^ ^]^ Copyright 2013, American Chemical Society. c) Synthesis of the NiPt@ZIF‐8 NPs, and d,e) corresponding morphology characterization. Reproduced with permission.^[^ ^18^ ^]^ Copyright 2018, American Chemical Society.

The earliest MOF used for hydrogen production from AB was MIL‐101 in the research work of Xu et al. in 2012 (**Figure** **7**a,b). Ultrafine Pt NPs anchored on MIL‐101 dehydrogenated AB in 3 min.^[^ ^155^ ^]^ The following year, Xu et al. reported the first AuNi alloy anchored in MIL‐101 using the dual‐solvent method. AuNi NPs have a size of 2.0–5.0 nm and catalyze the complete dehydrogenation of AB within 5 min.^[^ ^162^ ^]^ Didier et al. used ZIF‐8 as a support to study the optimal combination of Pt and Ni (Figure 7c). When the Ni:Pt ratio is 2:1, Ni_2_Pt@ZIF‐8 has the optimal synergistic effect and promotes the oxidation of the addition of O—H bond. The hydrogen produced from AB using Ni_2_Pt@ZIF‐8 follow the tandem reaction for hydrogenation.^[^ ^18^ ^]^

**Figure 8:**
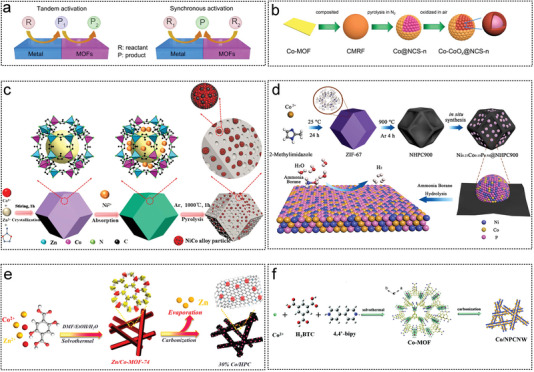
The MOF as non‐noble metal catalyst substrates for AB hydrolysis 1. a) The formation mechanism of the Cu*_x_* @Co_1‐_ *_x_*‐MOF/5 nanocatalysts and the corresponding XRD pattern. Reproduced with permission.^[^ ^31^ ^]^ Copyright 2021, Elsevier. b) Illustration of synthesis from Co‐based MOF precursor to Co‐CoO*_x_* @NCS‐n catalyst and corresponding morphology characterization. Reproduced with permission.^[^ ^20^ ^]^ Copyright 2019, American Chemical Society. c) Synthesis diagram of Nico‐NC catalyst obtained by in situ pyrolysis of ZIF‐67 with Ni^2+^ on the surface. Reproduced with permission.^[^ ^145^ ^]^ Copyright 2021, Elsevier. d) Illustration of the formation process of the carbonized ZIF‐67 MOF precursor as a supports‐supported bimetallic phosphating Ni_0.23_Co_0.19_P. Reproduced with permission.^[^ ^120^ ^]^ Copyright 2020, American Chemical Society. e) The synthesis route of optimum catalyst Co/Zn‐MOF‐74 and Co/HPC. Reproduced with permission.^[^ ^107^ ^]^ Copyright 2019, American Chemical Society. f) The synthesis route of optimum catalyst Co/NPCNW. Reproduced with permission.^[^ ^81^ ^]^ Copyright 2017, Royal Society of Chemistry.

Li et al. reported an interfacial ensemble of Cu NPs and Co‐MOFs, with Cu NPs and Co^2+^ in the MOF as the catalytically active sites at the interface (**Figure** **8**a). A synchronous activation model for metal particles/MOFs is proposed, which differs from the previous tandem activation.^[^ ^31^ ^]^ ZIF‐67 is most commonly used as a precursor for synthesizing of AB hydrolysis catalysts owing to its simple of synthesis and confinement to metal ions. The birdcage‐type catalyst designed based on ZIF‐67 can effectively prevent the active component from leaching leached out of the solution, thus ensuring a high activity of 5562 mL min^−1^ g_Co_ and durability at 298 K (Figure 8b).^[^ ^20^ ^]^ Zhang et al. formed a NiCo alloy with a uniform particle size by ultrahigh temperature (1000) pyrolysis. Owing to the abundance of nitrogen atoms and the positive synergy between Co and Ni, NiCo‐NC showed higher activity (35.2 min^−1^) than Co‐NC (Figure 8c).^[^ ^145^ ^]^ Peng et al. introduced P to form metal phosphates in NiCo alloys in a similar way to further improve the AB water‐interpreted hydrogen activity, with a TOF up to 125.2 min^−1^ (Figure 8d).^[^ ^120^ ^]^ Yang et al. used Co/Zn‐MOF as a precursor to form highly dispersed Co NPs on the HPC surface. Zn is a sacrificial agent that limits the agglomeration of NPs during pyrolysis. The 30% Co/HPC‐900 catalyst showed intentional durability in AB hydrolysis, with at least 12 repetitions (Figure 8e).^[^ ^107^ ^]^ Chen used a MOF‐driven method to construct Co‐based nanoscale particles and nitrogen‐doped support structures. The synergistic effect between structure and morphology resulted in improved durability, and the catalytic activity of the catalyst remained at 94.6% after ten uses (Figure 8f).^[^ ^81^ ^]^

**Figure 9:**
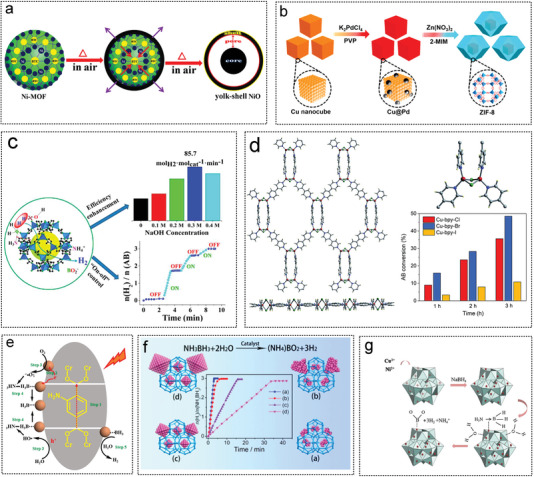
The MOF as non‐noble metal catalyst substrates for AB hydrolysis 2. a) Formation process from Ni MOF to yolk–shell NiO microspheres. Reproduced with permission.^[^ ^254^ ^]^ Copyright 2015, American Chemical Society. b) MOF‐derived CuPd@ZIF‐8 catalyst form schematic illustrations. Reproduced with permission.^[^ ^253^ ^a]^ Copyright 2020, American Chemical Society. c) Preparations of the nanocatalysts M/ZIF‐8, M = (Fe, Co, Ni, Cu). Reproduced with permission.^[^ ^77^ ^]^ Copyright 2017, American Chemical Society. d) Cu‐bpy‐Y (Y = Cl, Br and I) MOFs: the relationship between morphology and catalytic activity toward AB hydrolysis. Reproduced with permission.^[^ ^255^ ^]^ Copyright 2019, Royal Society of Chemistry. e) Possible AB hydrolysis mechanisms under light with Cu/MIL‐101 catalysts. Reproduced with permission.^[70]^ Copyright 2016, American Chemical Society. f) Schematic illustration for synthesis of MIL‐101 supported Co NPs and hydrogen generation from AB. Reproduced with permission.^[^ ^74^ ^]^ Copyright 2017, American Chemical Society. g) Synthesis of MIL‐101 loaded CuNi NPs by sodium borohydride reduction method. Reproduced with permission.^[^ ^82^ ^]^ Copyright 2017, Elsevier.

Using Ni‐MOF as a precursor, Wang designed and synthesized a yolk–shell NiO microstructure catalyst by exploiting the temperature difference during the calcination process (**Figure** **9**a). In the process of heat treatment at 500 °C, the external forces between the NiO shell and Ni‐MOF and the internal forces caused by Ni‐MOF shrinkage are key factors for the formation of the micro‐nano structure of the yolk–shell NiO microstructure.^[^ ^254^ ^]^ Jiang prepared a CuPd@ZIF‐8 catalyst via the synergistic action of CuMOF and ZIF‐8, and the catalyst had a large specific surface area (1077 m^2^ g^‐1^) conducive to the hydrolysis of AB (Figure 9b).^[^ ^253^ ^a]^ Astruc and his group synthesized Fe, Co, Ni, and Cu NPs on a ZIF‐8 surface using the sodium borohydride reduction method in situ (Figure 9c).^[^ ^77^ ^]^ The corresponding TOF values of FeNPs/ZIF‐8, CuNPs/ZIF‐8, CoNPs/ZIF‐8 and NiNPs/ZIF‐8 were 2.5, 5.6, 19.4, and 85. 7min^−1^, respectively. Stylianou et al. designed the effect of a halogen atom (Cl, Br, and I) doped Cu‐bpy catalyst on the hydrolysis activity of AB (Figure 9c). The decrease electronegativity from Cl^−^ to I^−^ leads to a decrease in the positive charge density of Cu^+^ and an increase in catalytic activity (Figure 9d).^[^ ^255^ ^]^ Yamashita et al. studied the catalytic activity of a non‐noble metal supported on a Chromium‐based MOF for AB hydrolysis under light conditions. Hydroxyl radicals, superoxide anions, and electron‐rich non‐noble metals formed under light are responsible for this increased activity (Figure 9e).^[^ ^70^ ^]^ Su et al. studied the relationship between the crystallinity, particle size of metal NPs, and AB hydrolysis activity. Four Co‐based NPs with different particle sizes were successfully constructed on MIL‐101. The experimental results confirmed that amorphous Co showed higher catalytic activity than crystalline NPs, with a TOF of up to 51.4 min^−1^ (Figure 9f).^[^ ^74^ ^]^ Chen et al. obtained amorphous Ni/C composite materials by in‐situ reduction of potassium borohydride and calcination of Ni‐MOF at high temperature, and the size of Ni NPs was about 10 nm. The hydrogen release rate at room temperature was 834 mL min^−1^ g^−1^.^[^ ^57^ ^]^ Zhou et al. discussed in detail the structure, morphology, size, composition and specific area of the catalysts loaded with different CuNi NPs, and examined the activity, influencing factors and stability of the catalysts by comparing them with AB hydrolysis catalysts (Figure 9g). The catalytic activity of CuNi bimetallic catalyst Cu_2_Ni_1_@MIL‐101 for AB hydrolysis was 20.9 min^−1^, and the *E*_a_ was 32.2 KJ mol^−1^.^[^ ^82^ ^]^

### 2.3 M–O Stabilized Interaction of Metal Oxide

Metal oxides such as TiO_2_,^[^ ^215^ ^]^ CeO_2_,^[^ ^89^ ^]^ Al_2_O_3_,^[^ ^256^ ^]^ etc. are used as chemical promoters or supports to catalyze the efficient hydrogen release of AB. Metal oxides have the advantages of an adjustable metal valence state, controllable surface oxygen concentration and low price.^[^ ^39^, ^257^ ^]^ TiO_2_ has nontoxic properties, low cost, strong oxidation capacity, high corrosion resistance, and a variety of isomers, and it is the most commonly used catalyst support for AB.^[^ ^231^ ^]^ Oxygen vacancies (V_O_) reshape the active site configuration of catalysts by changing the local charge distribution and electron energy of metal oxides.^[^ ^39^, ^258^ ^]^ Metal–support interactions^[^ ^21^, ^259^ ^]^ and hydrogen spillover effects^[^ ^256^ ^]^ also controllable means to regulate the catalyst structure and promote hydrolysis. Among the various supported catalysts, metal oxides are the most widely studied catalysts for AB hydrolysis. The role of metal oxides in catalytic hydrolysis is most fully analyzed, especially the exact effect of oxygen in the support on the metal.

**Figure 10:**
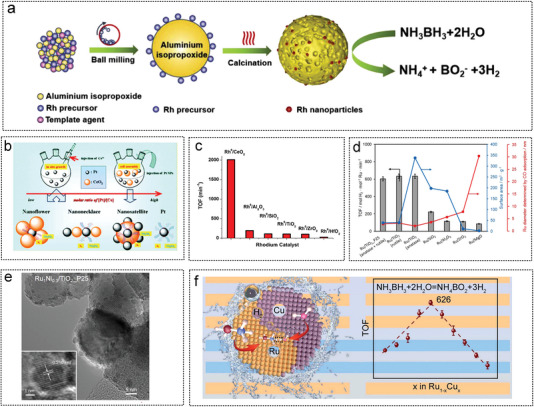
The metal oxide as noble metal catalyst substrates for AB hydrolysis. a) Illustration of mechanical synthesis of Rh/ meso‐Al_2_O_3_. Reproduced with permission.^[^ ^219^ ^]^ Copyright 2022, Elsevier. b) Illustration of Pd‐CeO_2_ formation process. Reproduced with permission.^[^ ^260^ ^]^ Copyright 2012, Royal Society of Chemistry. c) Comparison of TOF value of rhodium with high and low load for AB hydrolysis. Reproduced with permission.^[^ ^37^ ^]^ Copyright 2016, Elsevier. d) The influence of different oxide supports on the particle size of Ru NPs and the hydrogen production performance of AB. Reproduced with permission.^[21]^ Copyright 2016, American Chemical Society. e) Morphology analysis of Ru_1_Ni_0.3_/TiO_2_‐P25 catalyst. Reproduced with permission.^[^ ^21^ ^]^ Copyright 2016, American Chemical Society. f) Mechanism analysis of AB hydrolysis catalyzed by RuCu supported on TiO_2_ and TOF value of Different Cu doping ratio. Reproduced with permission.^[^ ^215^ ^]^ Copyright 2021, Elsevier.

An Rh/meso‐Al_2_O_3_ catalyst with a mesoporous structure and high specific surface area prepared by the mechanical method was used for hydrogen production from AB hydrolysis (**Figure** **10**a).^[^ ^219^ ^]^ Zhang et al. designed nanoflowers, nanosatellites and nano‐necklaces according to different exposed active surface areas. The nano‐necklaces showed higher catalytic activity for AB hydrolysis (Figure 10b).^[^ ^260^ ^]^ Saim Ozkar explored the effects of different supports (CeO_2_, Al_2_O_3_, SiO_2_, TiO_2_, ZrO_2_, and HfO_2_) on AB hydrolysis (Figure 10c). The valence state of cerium in the supports CeO_2_ changes from +5 to +3. The negative charge generated on the surface is favorable for Rh binding and improves the catalytic activity. At the same time, loading also affected the catalytic activity. When the loading decreased from 4.0 to 0.1% wt, the catalytic activity increased from 160 to 2010 min^−1^.^[^ ^37^ ^]^ Yamashita et al. systematically studied the relationship between particle size, specific surface area, and TOF with different supports (P25, rutile, anatase, SiO_2_, Al_2_O_3_, ZrO_2_, and MgO_2_). Catalysts using supported TiO_2_ generally have higher catalytic activity, with TOFs of Ru/TiO_2_ P25, Ru/TiO_2_ (rutile), and Ru/TiO_2_ (anatase) were 604, 620, and 610 min^−1^, respectively (Figure 10d). RuNi NPs were highly dispersed on TiO_2_ with a particle size of 2.3 nm, and the particle size of the catalyst was positively correlated with its catalytic activity. (Figure 10e). Simultaneously, the introduction of the second metal Ni further improved the catalytic activity to 914 min^−1^. The Pt_0_/Co_3_O_4_ catalyst designed by Özkar has excellent durability, with no significant decline after repeated use for ten times. Li et al. formed a RuCu alloy catalyst by replacing part of the precious metal Ru with Cu and systematically studied the influence of Cu doping amount from 10 to ‐100% on the catalytic activity. The *r*_b_ values of different RuCu ratios show a volcanic structure, and the best *r*_b_ value of 1.1 × 10^5^ mL min^−1^ g_Ru_ was found when the Cu content was 40% (Figure 10f). The subsequent increase in the Cu content led to the covering of Ru atoms and reduced the catalytic activity.^[^ ^215^ ^]^

**Figure 11:**
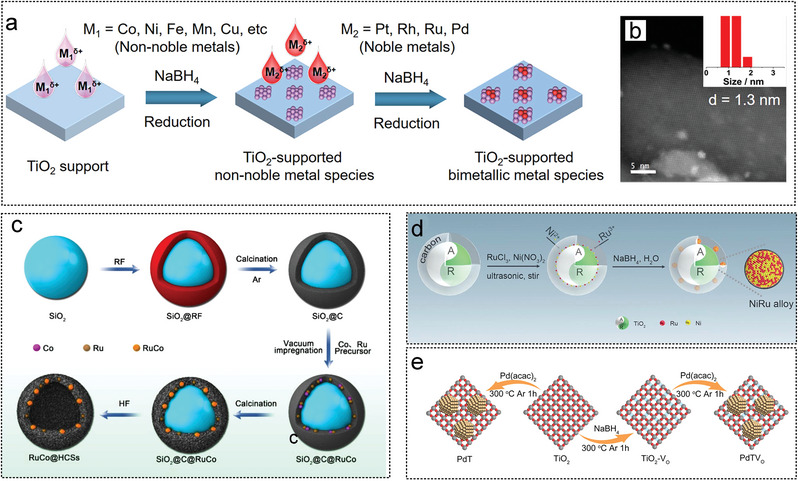
The metal oxide as noble metal catalyst substrates for AB hydrolysis. a) Schematic illustration of the preparation of TiO_2_‐supported bimetallic catalysts via step‐by‐step reduction method. b) HAADF‐STEM image of Pt_0.25%_Co_3%_/TiO_2_. Reproduced with permission.^[^ ^23^ ^]^ Copyright 2023, American Chemical Society. c) Illustration of the synthesis process of RuCo@ porous carbon by SiO_2_ template and high temperature carbonization method. Reproduced with permission.^[^ ^27^ ^b]^ Copyright 2019, American Chemical Society. d) Illustration of RuNi alloy synthesis supported on TiO_2_. Reproduced with permission.^[^ ^216^ ^]^ Copyright 2021, Elsevier e) Schematic diagram of Pd NPs supported on an oxygen‐rich vacancy TiO_2_. Reproduced with permission.^[^ ^232^ ^]^ Copyright 2021, Wiley‐VCH.

Sun et al. developed a stepwise reduction strategy to synthesize a range of small and highly dispersed bimetallic species on various metal oxide carriers (**Figure** **11**a). The introduction of Co species can significantly improve the catalytic activity of various noble metals (e.g., Pt, Rh, Ru, and Pd) in AB hydrolysis reactions compared to other non‐precious metals. The particle size of Pt_0.25%_–TiO_2_ was 1.8 nm and that of Pt_0.25%_Co_3%_/TiO_2_ was 1.3 nm (Figure 11b). The optimized Pt_0.25%_Co_3%_/TiO_2_ catalyst exhibited ultrahigh hydrogen production rates in AB hydrolysis, showing TOF of 2250 mol H_2_ mol_Pt_^−1^ min^−1^ at 298 K.^[^ ^23^ ^]^ Peng et al. reported a Ru–Co alloy (Ru, 1.8 wt%) loaded with hollow carbon ball supports prepared by vacuum impregnation and pyrolysis (Figure 11c). The Ru/Co ratio can be precisely controlled by direct reduction of ruthenium and alloy. The RuCo alloy provides a high TOF of 784 molH_2_ min^−1^ for the hydrolysis of AB at ambient temperature conditions. The excellent catalytic performance of RuCo@HCSs is attributed to its special hollow‐embedded structure and the synergy between the carbon shell and RuCo alloy.^[^ ^27^ ^b]^ Li et al. reported on NiRu alloys anchored to the surface of nitrogen‐doped carbon‐coated titanium dioxide (Figure 11d). The surface charge is regulated by the alloying effect of NiRu and the metal–support interaction between the alloy and the composite supports. The optimized surface charge corresponds to a low *E*_a_ of H_2_O and AB molecules. NiRu/TCN showed the highest catalytic activity (2.51 × 10^5^ mL min^−1^ g_Ru_^−1^) for the hydrolysis of NH_3_BH_3_ at 298 K.^[^ ^216^ ^]^ At the same time, Li also constructed Pd NPs in TiO_2_ rich in oxygen vacancies to form engineering vacancy‐atom ensembles (Figure 11e). The H_2_O molecules tended to dissociate at the V_O_ site, and AB molecules tended to dissociate at the Pd atom. On V_O_, the H atom from water combines with the H atom from AB on Pd atom to form H_2_.^[^ ^232^ ^]^ Yang synthesized a PtNi alloy catalyst on Al_2_O_3_. In AB hydrolysis, electrons are transferred from the less electronegative Ni atom to the more electronegative Pt atom, forming an electric dipole between the adjacent Pt–Ni pairs. The new local electric field generated by the Pt–Ni dipoles affects the electron distribution in the AB molecules, and the electron redistribution of the AB molecules is more likely to interact with the PtNi catalysts to form activated complexes. Therefore, the dehydrogenation of Al_2_O_3_–PtNi to AB shows higher catalytic activity than Al_2_O_3_–Pt and Al_2_O_3_–Ni.^[^ ^211^ ^]^

**Figure 12:**
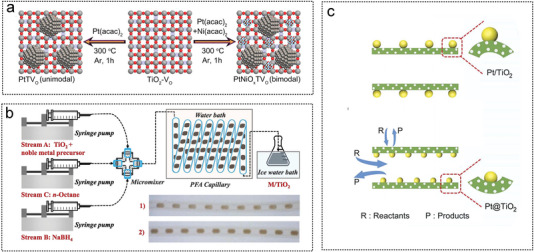
The metal oxide as noble metal catalyst substrates for AB hydrolysis. a) Synthesis of Pt and NiO*_x_* supported on single face exposed TiO_2_. Reproduced with permission.^[^ ^231^ ^]^ Copyright 2021, Wiley‐VCH. b) Schematic diagram of a device for continuous synthesis of TiO_2_‐supported noble metal NPs by fractional flow. Reproduced with permission.^[^ ^261^ ^]^ Copyright 2022, Elsevier. c) Reaction process mechanism for Pt@TiO_2_ and Pt/TiO_2_ catalyst. Reproduced with permission.^[^ ^242^ ^]^ Copyright 2022, Elsevier.

The synthesis of a material consisting of Pt NPs and NiO*_x_* nanoclusters supported by single‐sided TiO_2_ is depicted in **Figure** **12**a. Dual‐mode oxygen vacancies significantly promoted H_2_O dissociation in two ways. First, the ensemble‐inducing effects of Pt and V_O_ in titanium dioxide drive the activation of H_2_O molecules. Second, the electron promotion effect caused by electron transfer from V_O_ to Pt in NiO*_x_* further enhances the ability of Pt to dissociate H_2_O and AB.^[^ ^231^ ^]^ Li et al. further studied and established the V_O_–Ti ensemble project on a Ru catalyst to improve the hydrolysis activity of AB. V_O_ in the V_O_–Ti ensemble acts as an electron accelerator to transfer electrons to Ru atoms on the surface, and electron‐rich Ru enhances water separation activity. The TOF of 1.5‐RTV_O_‐4 for AB hydrolysis was as high as 1370 min^−1^, exceeding the reference value set by previous Ru catalysts.^[^ ^39^ ^]^ Chen proposed a method for continuously synthesizing TiO_2_‐supported noble metal NPs (M/TiO_2_‐Mr, M = Pd, Pt, or Au) in a staged flow without a stabilizer (Figure 12b). Owing to the enhanced mixing performance of the micromixer, the size of the noble metal NPs can be controlled by simply reducing the metal precursor with NaBH_4_ in the presence of TiO_2_ without using stabilizer. Compared with the batch method, the prepared M/TiO_2_‐MR had a smaller noble metal particle size and better dispersion, exposing more active sites.^[^ ^261^ ^]^ Qin reported the synthesis of a porous TiO_2_ nanotube‐constrained Pt catalyst by template‐assisted atomic layer deposition (ALD) for hydrolytic dehydrogenation of NH_3_BH_3_ (Figure 12c). Compared with supported Pt/TiO_2_, ultrafine Pt NPs were modified on the surface of nanotube, and the Pt–TiO_2_ interface position increased. These factors contribute to the excellent catalytic performance of the confined Pt@TiO_2_ catalyst.^[^ ^242^ ^]^

**Figure 13:**
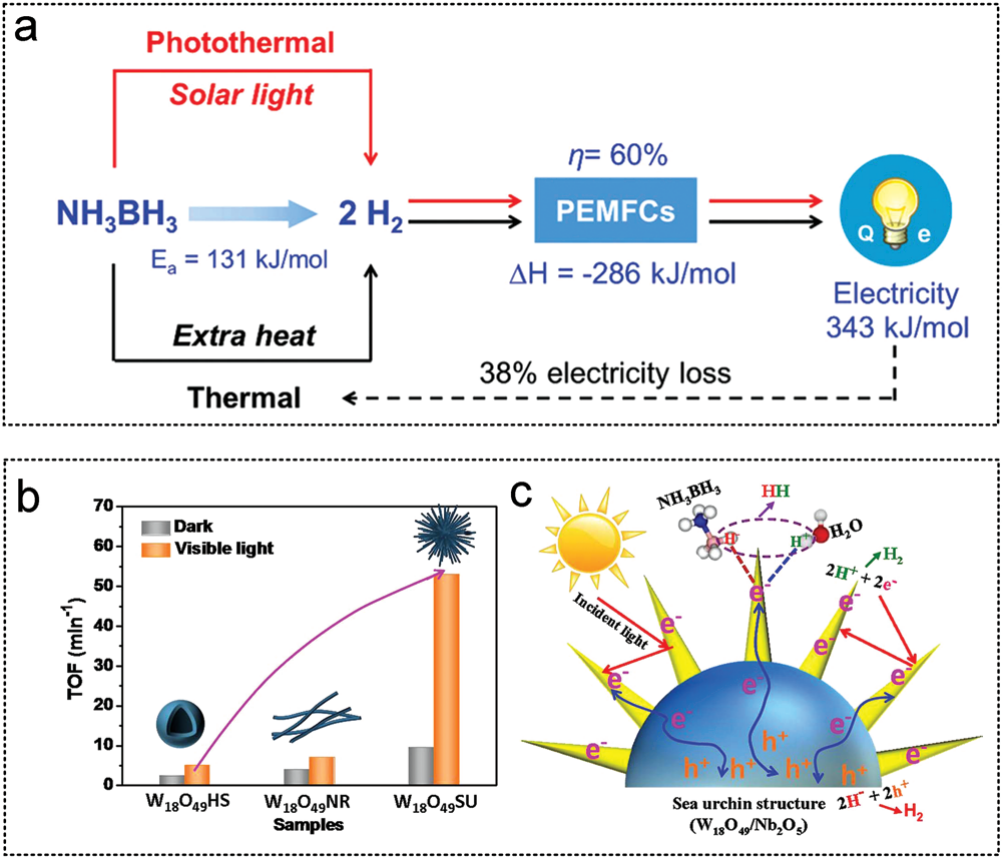
The metal oxide as non‐noble metal catalyst substrates for AB hydrolysis. 01. a) Effects of catalyst particle size on photothermal activation efficiency (PTAE), photothermal strategies applied in simulated environments, and efficiency comparison of different AB hydrogen production methods applied in vehicles. Reproduced with permission.^[^ ^262^ ^]^ Copyright 2020, Wiley‐VCH. b) Study on the photocatalytic performance and mechanism of W_18_O_49_ photocatalysts with different morphology of sea urchin (W_18_O_49_SU), nanorods (W_18_O_49_NR) and hollow spheres (W_18_O_49_HS) for the hydrolysis of AB. c) Mechanism of light harvesting and photogenerated charge transfer in semiconductors. Reproduced with permission.^[^ ^132^ ^]^ Copyright 2020, Wiley‐Elsevier.

Xu et al. proposed a new solar‐powered low‐temperature hydrogen production strategy for AB. Ti_2_O_3_ was used as a photothermal catalyst to activate AB reactants to produce hydrogen (**Figure** **13**a). Ti_2_O_3_ NPs with high chemical stability and a narrow band gap were prepared by the reduction conversion method to generate of 2.0 equivalent hydrogen from AB at room temperature. The photothermal activation efficiency of the nanosized Ti_2_O_3_ particles reached 35%. Assisted by the CuCl_2_ promoter, the equivalent of 2.0 hydrogen was successfully released under 1.0 solar irradiation at 70 °C, revealing its potential application in practical vehicles based on proton‐exchange membrane fuel cells.^[^ ^262^ ^]^ Co‐CoO*_x_*/TiO_2_@N‐C (COTC) catalysts anchored in TiO_2_ were prepared using the sol‐gel method and high‐temperature carbonization methods. The catalyst effectively promoted the release of hydrogen during the hydrolytic dehydrogenation of AB, and a high hydrogen yield of 5905 mL min^−1^ g_Co_^−1^ was obtained at room temperature. When the catalyst was applied to AB hydrolysis for the fifth time, the initial catalytic activity remained at 85%. The synergistic action of Co, Co_3_O_4_, and TiO_2_ promoted the rate‐limiting step of H_2_O dissociation and activation by reducing the *E*_a_ of the H_2_O molecules.^[^ ^111^ ^]^ Wang et al. consider that the morphology of the catalyst was the key factor affecting its photocatalytic performance. W_18_O_49_ photocatalysts with different morphologies of sea urchin (W_18_O_49_SU), nanorods (W_18_O_49_NR) and hollow spheres (W_18_O_49_HS) were prepared. In the process of AB photocatalytic hydrolysis, W_18_O_49_SU showed a TOF value of 53.1 min^−1^, and the TOF of W_18_O_49_HS was 10.4 and 7.5 times that of W_18_O_49_NR and hollow spheres W_18_O_49_HS, respectively (Figure 13b).^[^ ^132^ ^]^ The excellent photocatalytic H_2_ evolution activity of AB hydrolysis is due to the synergistic effect of enhanced light harvesting, charge separation and electron enrichment at the tips of rod. (Figure 13c).

**Figure 14:**
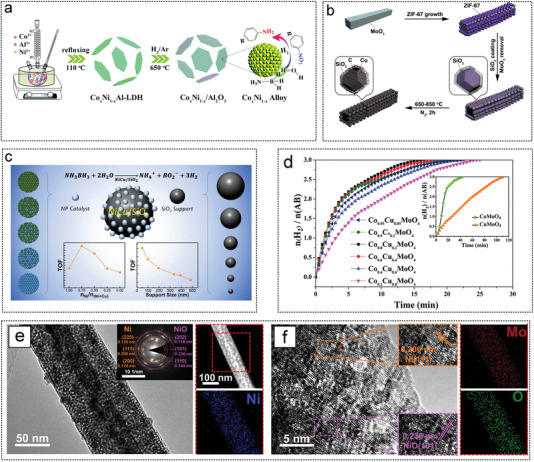
The metal oxide as non‐noble metal catalyst substrates for AB hydrolysis. 02. a) Illustration of preparation of Co*_x_* Ni_1‐_ *_x_*/Al_2_O_3_ nanosheets. Reproduced with permission.^[^ ^95^ ^]^ Copyright 2019, Royal Society of Chemistry. b) Schematic diagram of Co@N‐doped carbon porous nanocages anchored at silica. Reproduced with permission.^[^ ^93^ ^]^ Copyright 2019, American Chemical Society. c) Relationship between NiCu/SiO_2_ particle size and TOF. Reproduced with permission.^[^ ^97^ ^]^ Copyright 2019, American Chemical Society. d) Hydrogen production curve of Co*_x_* Cu*_y_* MoO_4_ catalysts. Reproduced with permission.^[^ ^85^ ^]^ Copyright 2018, American Chemical Society. e,f) morphology analysis of Ni/NiO@MoO*_x_* catalyst. Reproduced with permission.^[^ ^131^ ^]^ Copyright 2021, American Chemical Society.

Wang et al. designed a series of ultrafine CoNi alloy NPs embedded in alumina nanosheet (Co*_x_* Ni_1‐_ *_x_*/Al_2_O_3_, where X represents the Co content) catalysts. By changing the content of Ni in CoNiAl‐layered double hydroxide (LDH) precursor, the composition, size, morphology, and catalytic performance of the catalyst can be easily controlled. Cu bimetallic NPs with different Ni/Cu compositions were synthesized by adjusting the ratio of nickel to copper acetyl pyruvate (**Figure** **14**a).^[^ ^95^ ^]^ Liu et al. reported the synthesis of a Co@N‐doped porous carbon catalyst containing SiO_2_ by calcination of the zeolite imidazole framework ZIF‐67 at high temperatures in an N_2_ atmosphere (Figure 14c). The SiO_2_ surface layer in the precursor provides an additional surface for the dispersion of CoNPs to reduce their size. In addition, the SiO_2_ layer makes the Co@N‐doped porous carbon highly ordered in the catalyst, which may facilitate the mass transfer in the catalyst.^[^ ^93^ ^]^ When bimetallic and mono‐metallic NPs were used as catalysts for the hydrolysis of AB, their catalytic activity was related to their composition. The activity of the NiCu/SiO_2_ catalyst increased with a decrease in the SiO_2_ particle size (Figure 14c). SiO_2_ spheres with higher curvature and longer distances between particles reduce the agglomeration of NiCu NPs and have better stability. NiCu NPs supported on the smallest SiO_2_ showed a higher TOF of 1516 mol_H2_ mol_metal_^−1^ h^−1^ and good reusability in continuous AB hydrolysis compared with unsupported NPs, indicating strong metal‐supported interactions.^[^ ^97^ ^]^ Li prepared a series of Co*_x_* Cu_1−_ *_x_* MoO_4_ microspheres composed of nanosheets using a green method that did not contain surfactants, complexing agents, or organic solvents. The catalytic activity of Co*_x_* Cu_1−_ *_x_* MoO_4_ for in hydrolytic dehydrogenation of AB was studied for the first time. Co_0.8_Cu_0.2_MoO_4_ had a synergistic effect on AB hydrolytic dehydrogenation. The active substance in AB hydrolysis was the CoCu alloy NPs supported by Co_0.8_Cu_0.2_MoO_4_ microspheres (Figure 14d).^[^ ^85^ ^]^ Xiao synthesized highly loaded heterogeneous Ni/NiO NPs (Ni/NiO@MoO*_x_*) with different Ni/NiO weight ratios fixed on 1D porous MoO*_x_* nanorods (Figure 14e,f). The Ni/NiO@MoO*_x_*‐50H catalyst (synthesized in a 50% H_2_/50% Ar reduction atmosphere) had the best Ni/NiO mass ratio (73.96/26.04). NiO bridging stably connects the Ni and MoO*_x_* supports, giving Ni/NiO@MoO*_x_*‐50 great durability.^[^ ^131^ ^]^ Lu et al. synthesized Ni nanocatalysts (Ni‐CeO*_x_*/graphene) doped with CeO*_x_* and supported on graphene through a simple chemical reduction approach. The activity of the synthesized Ni–CeO*_x_*/graphene nanocomposites is 49 times higher than that of NiNPs, showing excellent catalytic activity, and the TOF is up to 68.2 min^−1^. CeO*_x_* plays a key role in the high activity of graphene‐based multicomponent composite catalysts.^[^ ^89^ ^]^ Yao et al. prepared a titanium‐supported NiCo_2_O_4_ nanofilm array (NiCo_2_O_4_/Ti) using a simple method and studied its catalytic performance in AB hydrolysis to produce hydrogen. The apparent *E*_a_ of AB hydrolysis over the NiCo_2_O_4_/Ti catalyst was 17.5 kJ mol^−1^. More importantly, the NiCo_2_O_4_/Ti catalyst retained about 90% of its original catalytic activity after ten cycles.^[^ ^65^ ^]^

### 2.4 Graphite Carbon Nitride (g‐C_3_N_4_)

Graphite carbon nitride (g‐C_3_N_4_), prepared by thermal polymerization of melamine, urea, thiourea, and dicyandiamide, has a layered structure similar to graphene, with weak van der Waals forces between layers.^[^ ^263^ ^]^ g‐C_3_N_4_ has the advantages of simple preparation, good thermal and chemical stability, non‐toxic, low cost, and broad application prospect in the field of catalytic hydrogen generation from AB.^[^ ^235^ ^]^ The modified porous g‐C_3_N_4_ has a different energy band structure, photoluminescence lifetime, and photocurrent density under visible light irradiation, resulting in different separation efficiency of photogenerated supports. These features can enhance visible‐light‐driven hydrogen production activity.^[^ ^80^ ^]^ The semiconducting nature of g‐C_3_N_4_ makes it possible for the supported catalyst to improve its performance rapidly. In the next step, efficient catalysts must be designed based on light exposure, and the mechanism of action of light conditions on catalytic performance must be analyzed.

**Figure 15:**
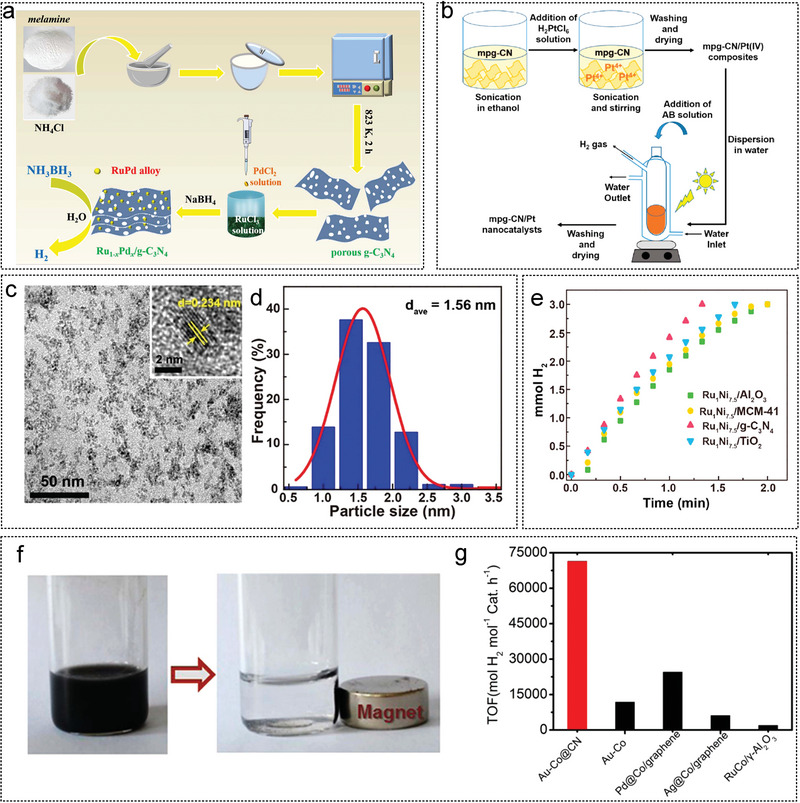
The g‐C_3_N_4_ as noble metal catalyst substrates for AB hydrolysis. a) Illustration of the synthesis process of Ru_1‐_ *_x_* Pd*_x_*/g‐C_3_N_4_ catalyst. Reproduced with permission.^[^ ^206^ ^]^ Copyright 2020, Elsevier. b) Schematic diagram of synthesis of MPG‐CN/Pt catalyst. Reproduced with permission.^[^ ^25^ ^c]^ Copyright 2020, American Chemical Society. c) Morphology, d) particle size of Ru_0.1_Co_0.9_/G‐C_3_N_4_ catalysts. Reproduced with permission.^[224]^ Copyright 2021, Royal Society of Chemistry. e) Analysis of catalytic AB hydrogen production by used RuNi/g‐C_3_N_4_ catalyst. Reproduced with permission.^[^ ^235^ ^]^ Copyright 2021, Elsevier. f) Magnetic separation photographs and g) hydrogen production activity and stability of Au‐Co@CN catalysts. Reproduced with permission.^[^ ^173^ ^]^ Copyright 2015, American Chemical Society.

Zheng et al. stabilized ultrafine bimetallic RuPd NPs on ultra‐thin porous graphite carbonitride using simple adsorption in situ reduction method (**Figure** **15**a). The catalytic activity of the Ru_0.85_Pd_0.15_/g‐C_3_N_4_ bimetal catalyst was higher than that of Ru/g‐C_3_N_4_ and Pd/g‐C_3_N_4_ single metal catalysts in AB hydrolytic dehydrogenation because of the co‐alloying effect. Ru_0.85_Pd_0.15_/g‐C_3_N_4_ has good catalytic activity and durability, and has good potential for hydrolyzing hydrogen production from chemical storage materials.^[^ ^206^ ^]^ Metin et al. prepared mesoporous graphite carbon nitride (MPG‐CN/Pt) composites by in situ reduction (Figure 15b). The in situ synthesis method is advantageous for synthesizing of MPG‐CN/Pt catalyst. Under white light irradiation, the photocatalytic activity for AB hydrolysis increased by 2.25 times when the Pt loading was 5.94 wt%. This is the demonstration of the catalytic activity of in situ synthesized mpG‐CN as a suitable support for stabilizing PtNPs for photocatalytic hydrogen precipitation by AB hydrolysis.^[^ ^224^ ^]^ Fan et al. synthesized an ultrafine RuCo alloy with g‐C_3_N_4_ as a support material using a one‐step in‐situ method, with a particle size 1.56 nm (Figure 15c,d). The optimal Ru_0.1_Co_0.9_/g‐C_3_N_4_ catalyst can achieve complete conversion of AB to hydrogen with a TOF of up to 1260 mol H_2_ min^−1^. Kinetics studies showed that AB hydrolysis is a zero‐order reaction with an apparent *E*_a_ as low as 22.5 kJ mol^−1^. The excellent catalytic activity is due to the small size of the RuCo alloy, rich surface‐active sites, a synergistic effect between matrix and bimetallic alloy NPs, and rich N functional groups in g‐C_3_N_4_. However, the durability needs further improvement.^[^ ^224^ ^]^ In addition, Fan et al. successfully loaded RuNi alloy onto g‐C_3_N_4_ using an in situ reduction method. The activity of the RuNi alloy on the Ru_1_Ni_7.5_/g‐C_3_N_4_ catalyst was 901 min^−1^. Ru_1_Ni_7.5_/g‐C_3_N_4_ has good recyclability and can maintain 46% of its initial catalytic activity after running 5 times. The high performance of Ru_1_Ni_7.5_/g‐C_3_N_4_ was attributed to the synergy between the small alloy NPs with abundant active sites and the composition‐regulated RuNi bimetal (Figure 15e).^[^ ^235^ ^]^ Chen designed magnetically recyclable carbon nitride‐supported Au‐Co NPs (Au‐Co@CN) (Figure 15f). The synergistic effect between the non‐noble metal Co and Au NPs and the Motty–Schottky effect at the metal–semiconductor interface significantly promoted the catalytic performance of the Au‐Co@CN catalyst for the hydrolysis of AB. The TOF value of Au‐Co@CN catalyst was three times higher than that of the Au@Co nanoparticle catalyst (Figure 15f).^[^ ^173^ ^]^

**Figure 16:**
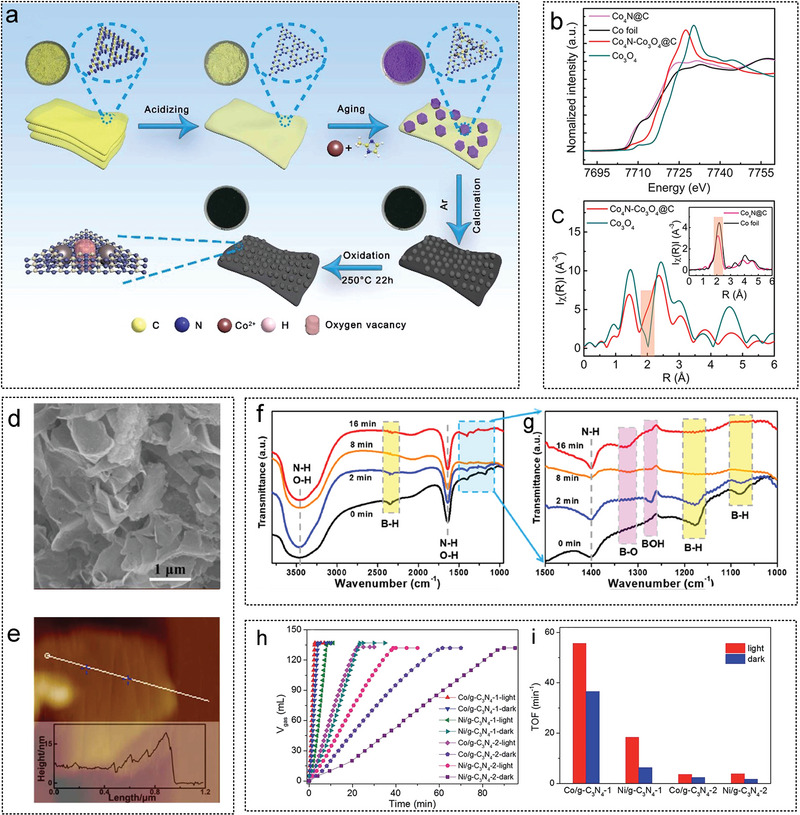
The g‐C_3_N_4_ as non‐noble metal catalyst substrates for AB hydrolysis. a) Schematic diagram of synthesis of Co‐CN‐O‐100 catalyst. Reproduced with permission.^[^ ^109^ ^]^ Copyright 2020, Elsevier. b,c) X‐ray absorption near‐edge characterization of Co_4_N‐Co_3_O_4_@C catalysts. Reproduced with permission.^[^ ^149^ ^]^ Copyright 2022, Wiley‐VCH. d,e) Comprehensive morphology characterization of g‐C_3_N_4_. Reproduced with permission.^[^ ^96^ ^]^ Copyright 2019, Royal Society of Chemistry. f,g) FT‐IR spectra and in situ mass spectrometry analysis of NiCu/CNS catalysts. Reproduced with permission.^[^ ^125^ ^]^ Copyright 2020, American Chemical Society. h) Time hydrogen production diagrams and i) corresponding TOF values for different catalysts under two different conditions. Reproduced with permission.^[^ ^79^ ^]^ Copyright 2017, Royal Society of Chemistry.

Li et al. prepared Co‐CN‐O‐100 nanocomposites using the deposition precipitation method and high‐temperature calcination methods (**Figure** **16**a). The catalytic performance of Co‐CN‐O‐100 catalysts for the hydrogen production of NH_3_BH_3_ was tested in an intermittent reactor. After oxidation treatment, the dehydrogenation capacity of the catalyst was significantly enhanced. The generation time of H_2_ is only 3.5 min, and the hydrogen production rate reached 5540 mL min^−1^ g_Co_^−1^ at 25 °C. Under NaOH free and mol L^−1^ NaOH conditions, the H_2_ generation times were 17 min and 3.5 min, respectively.^[^ ^109^ ^]^ In addition, Li et al. designed a Co_4_N‐Co_3_O_4_ interfacial structure by controlled nitridation and oxidation control and determined the effect of the interfacial active center consisting of Co_4_N and Co_3_O_4_ on AB hydrogen production. The Co_4_N‐Co_3_O_4_@C catalyst has high catalytic activity, and the TOF of hydrogen generation is up to 79 min^−1^. XPS and X‐ray absorption near‐edge analysis confirm the existence of Co—N and Co—O bonds (Figure 16b,c). The catalytic kinetics study confirmed that the hydrolysis of AB is a zero‐order reaction and is not limited by the diffusion of reactants. The reaction rate depends only on the structure of the catalyst. The stability of the Co_4_N–Co_3_O_4_ interface structure was verified by theoretical simulation, and the atomic interface‐exciting effect (AieE) was the main reason for its high catalytic activity.^[^ ^149^ ^]^ Li et al. prepared monodisperse Ni NPs with controllable sizes using a simple method and fixed them on graphitized carbon nitride (g‐C_3_N_4_) nanosheets by self‐assembly (Figure 16d,e). The Ni/g‐C_3_N_4_ composite catalyst showed good photocatalytic activity for hydrolytic dehydrogenation of AB under visible light. When the size of the NiNPs was 3.2 nm, the optimal AB hydrolysis rate was 18.7 mol_H2_ mol_cat_^−1^ min^−1^, and the apparent *E*_a_ is 36 kJ mol^−1^.^[^ ^96^ ^]^ When g‐C_3_N_4_ is irradiated with visible light, the electron–hole pair is photoexcited in both the conduction and valence bands. Electrons flowed through g‐C_3_N_4_ before recombination with holes. After the NiNPs are anchored on the surface of g‐C_3_N_4_, electrons can be rapidly transferred to NiNPs through the interface between g‐C_3_N_4_ and the NiNPs. Therefore, the surface‐active sites of the NiNPs are electron‐rich, facilitating the hydrolysis of AB. Wang et al. systematically studied the hydrolysis of AB on NiCu alloy supported carbon nitride nanosheets (Ni*_x_* Cu*_y_*/CNS). The TOF of AB hydrolyzed by the NiCu/CNS catalyst under visible light irradiation was nearly 3.5 times higher than that under dark irradiation. Fourier‐transform infrared spectroscopy (FT‐IR) was used to trace the hydrolysis process of the NiCu/CNS catalyst and the source of hydrogen. The peaks of the B—H bond at 1080, 1177, and 2347 cm^−1^ rapidly disappeared rapidly as the reaction time increased from 0 to 16 min. Meanwhile, the peaks of the B—OH and B—O bonds at 1270 and 1324 cm^−1^ can be observed as the reaction proceeds. The results of the infrared characterization proved that the B—H bond is broken and the B—O bond is formed during the reaction (Figure 16f,g).^[^ ^125^ ^]^ Su et al. prepared g‐C_3_N_4_ with different microstructures by thermal modification, and then Co and NiNPs were loaded on g‐C_3_N_4_ for the catalytic hydrogen precipitation of AB at room temperature. The modified porous g‐C_3_N_4_ has a different band structure, photoluminescence lifetime, and photocurrent density under visible‐light irradiation, leading to different separation efficiencies of the photogenerated supports. These characteristics help to regulate the electronic characteristics of Co and NiNPs in supported catalysts, resulting in significantly different and enhanced hydrogen evolution activity under visible light.^[^ ^80^ ^]^ Zhong et al. prepared a hybrid material of Ni_0.5_Co_0.5_O NPs on carbon nitride (NCN) treated with nitric acid for the AB hydrolysis. The carbon nitride substrate has two reaction centers, carbon as an electron acceptor and nitrogen as an electron donor, forming stable interfacial interactions with the NPs. Therefore, the NP‐NCN system has a hybrid electronic structure benefits to catalytic hydrogen production.^[^ ^102^ ^]^ Su et al. synthesized a series of non‐noble metal mono‐metal (Co, Fe, and Ni) and bimetallic (FeCo, CuNi, CuCo, FeNi, and NiCo) NPs supported by semiconductor graphitized carbon nitride (g‐C_3_N_4_) to catalyze the hydrogen evolution of AB under visible light irradiation at 298K. Compared to the activity of all the catalysts in a dark environment, the activity of the catalysts increased significantly under visible light irradiation. The TOF of the Co, CuCo, and FeCo catalysts synthesized in situ were 55.6, 75.1, and 68.2 min^−1^, respectively (Figure 16h,i).^[^ ^79^ ^]^ The enhanced activity can be attributed to the enrichment of the electron density of the active metal NPs under visible‐light irradiation caused by the Mott‐Schottky effect at the g‐C_3_N_4_–metal interface. Moreover, the catalytic activity of the catalyst strongly depends on the wavelength and intensity of the incident light, suggesting that visible‐light irradiation plays a key role in enhancing catalytic activity.

### 2.5 Synergistic Effect of MoC and Metal

MoC is often used as a support for catalysis and energy production because of its high melting point and hardness, good thermal and mechanical stability, and corrosion resistance.^[^ ^264^ ^]^ The synergistic effect of MoC and metals can lead to a unique H_2_O activation ability and high metal surface coverage.

**Figure 17:**
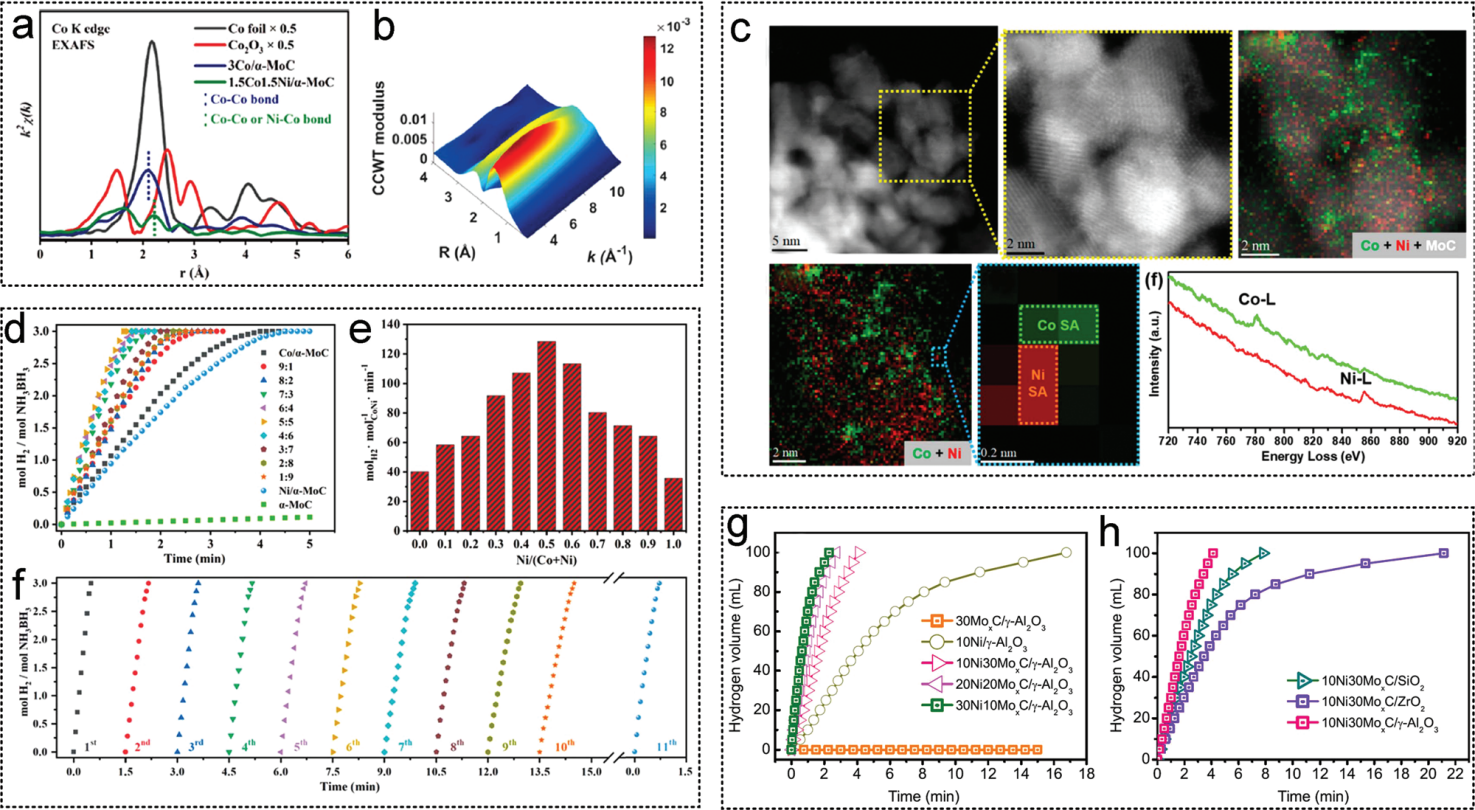
The MoC as metal catalyst substrates for AB hydrolysis. a,b) XANES profiles of Co K edge and WT analysis of CoNi/*α*‐MoC catalyst. c) HAADF‐STEM image and EELS spectra of 1.5Co1.5Ni/*α*‐MoC. d–f) Activity and stability of CoNi/*α*‐MoC catalysts. Reproduced with permission.^[^ ^33^ ^]^ Copyright 2021, American Chemical Society. g,h) Activity of 10Ni_3_0Mo*_x_* C with other different mixed supports. Reproduced with permission.^[^ ^137^ ^]^ Copyright 2021, American Chemical Society.

Ma et al. successfully synthesized highly dispersed CoNi on the surface of MoC using an impregnation method based on strong metal–support interaction.^[^ ^33^ ^]^ Extended X‐ray absorption fine structure profiles and wavelet transform show that Co and Ni mainly bind to C atoms, not Mo atoms, on the *α*‐MOC surface (**Figure** **17**a, b). At the same time, Co and Ni were highly dispersed on the *α*‐MoC surface, and no agglomeration of NPs. High‐angle annular dark‐field scanning transmission electron microscopy (HAADF‐STEM) and electron energy loss spectroscopy showed that most of the Co and Ni were highly dispersed on the *α*‐MoC surface, with only a few CoNi clusters. The short distance between the Co and Ni atoms is conducive to the synergistic interaction with the reactants during AB hydrolysis (Figure 17c). The results of hydrogen production showed that the Co Ni ratio affected the catalytic activity, and the best catalytic activity was achieved when the ratio of Co Ni was 1:1. The TOF value of 321.1min^−1^ of 1.5Co1.5Ni/*α*‐MoC was the highest reported for nonprecious metals (Figure 17d–f). Furthermore, Liu et al. studied the effect of additional supports, such as SiO_2_, ZrO_2_, and Al_2_O_3_ mixed with MoC, on the hydrolytic activity of AB (Figure 17g,h).^[^ ^137^ ^]^ These results confirm a strong synergistic effect between Al_2_O_3_ and MoC, which further improves the dispersion and stability of Ni. MoC provides new ideas for the designing and applying of catalysts with highly dispersed multiphase active centers and synergistic interactions for low‐temperature hydrolysis and hydrogen production reactions.

### 2.6 Promoting Effect of CNTs

CNTs are attractive catalyst supports because of their large specific surface area, high mechanical strength, hardness, thermal stability, excellent adsorption properties, and unique electrical properties.^[^ ^166^, ^265^ ^]^ A variety of transition metal–CNTs catalysts were prepared by impregnation reduction,^[^ ^165^ ^]^ replacement,^[^ ^43^ ^]^ high‐temperature carbonization^[^ ^266^ ^]^ or ALD deposition^[^ ^190^, ^242^, ^267^ ^]^ for hydrogen production from AB. The mechanism by which CNTs promote hydrogen production has been studied systematically using a variety of characterization techniques.^[^ ^266^ ^]^

**Figure 18:**
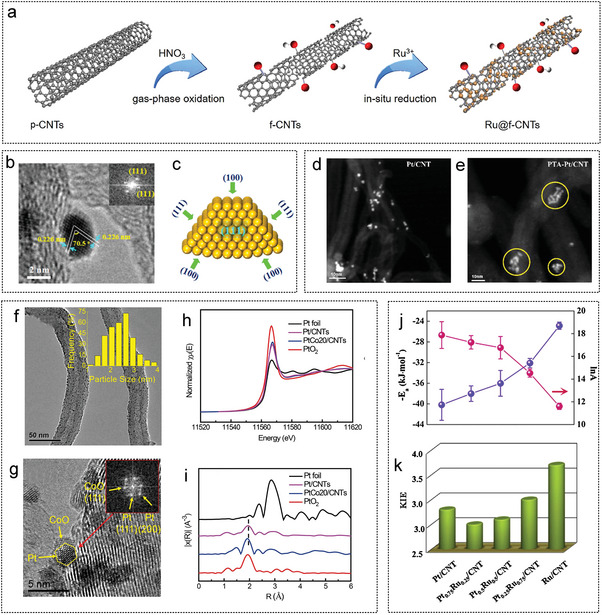
The CNTs as noble metal catalyst substrates for AB hydrolysis. a) Synthesis diagram and morphology characterization of Ru@f‐CNTs catalyst. Reproduced with permission.^[^ ^240^ ^]^ Copyright 2021, Elsevier. b,c) Morphology and crystal plane analysis of platinum supported on CNTs. Reproduced with permission.^[^ ^166^ ^]^ Copyright 2014, American Chemical Society. d,e) Morphology and XPS spectra analysis of Pt/CNT, STA‐Pt/CNT, PTA‐Pt/CNT and PMA‐Pt/CNT catalysts. Reproduced with permission.^[^ ^204^ ^]^ Copyright 2020, Elsevier. f,g) Morphology analysis of PtCo20/CNTs catalyst. h,i) XANES and EXAFS analysis of Pt foil, Pt/CNTs, PtCo20/CNTs and PtO_2_ catalysts. Reproduced with permission.^[190]^ Copyright 2018, Elsevier. j) *E*_a_ and k) kinetic isotope effect values of Pt/CNT, PT_0.75_Ru_0.25_/CNT, PT_0.5_Ru_0.5_/CNT, Pt_0.25_Ru_0.75_/CNT, and Ru/CNT catalysts. Reproduced with permission.^[^ ^178^ ^]^ Copyright 2017, Elsevier.

The introduction of N, O, or surface defects on the surfaces of CNTs promotes metal dispersion. Fan et al. constructed N and O defects on the surfaces of CNTs via nitric acid oxidative activation (**Figure** **18**a). The Ru‐based catalyst, Ru@f‐CNTs, had a uniform particle size of 1.71 nm. The catalytic activity and durability of the Ru@f‐CNTs catalyst were higher than those of the Ru@CNTs during AB hydrolysis.^[^ ^240^ ^]^ Chen et al. studied the relationship between crystal planes and catalytic activity using a Pt/CNTs catalyst. Kinetic tests and simulations revealed that the Pt crystal plane (111) has the highest catalytic activity of the two crystal planes (111) and (100) (Figure 18b,c). The catalytic activity of Pt has a volcanic relationship with the size of PtNPs, and the activity was highest when the particle size was 1.8 nm.^[^ ^166^ ^]^ Duan et al. studied three types of polyoxometalates: silicotungstic acid (STA), phosphotungstic acid and molybdophosphoric acid. STA had a positive effect on catalytic activity and durability. Compared with the other two types of polyformaldehyde, STA can improve the binding energy of Pt, thus promoting the reaction. The addition of STA inhibited the aggregation of Pt NPs and retained the active sites of Pt during the reaction process. At the same time, Duan et al. pointed out that defect‐rich CNTs formed by acid oxidation at high temperatures in an inert atmosphere are promising supports for inhibiting the agglomeration of Pt particles in durability tests (Figure 18d,e).^[^ ^204^ ^]^ Doping with non‐noble metals is also an effective strategy for improving activity. Highly dispersed Pt‐Co bimetallic NPs were prepared via ALD. The particle size of the 20%CoO doped PtCo NPs was 2.4 nm, and the TOF value reaches 675.1 min^−1^ (Figure 18f,g). In the PtCo20/CNTs samples, compared with Pt/CNTs, the radial distribution of the extended X‐ray Absorption Fine Structure spectra in the R space was slightly offset, indicating the interaction between Pt and Co. CoO deposited on or next to the Pt NPs changes the electronic properties of Pt accordingly, constructing a Pt–Co targeting interface (Figure 18h,i).^[^ ^190^ ^]^ In order to understand the role of H_2_O molecules in AB hydrogen production, Duan et al. conducted tests on different catalysts Pt/CNT, Pt_0.75_Ru_0.25_/CNT, Pt_0.5_Ru_0.5_/CNT, Pt_0.25_Ru_0.75_/CNT and Ru/CNT in H_2_O and D_2_O solutions (Figure 18j,k).^[^ ^178^ ^]^ PtRu/CNT bimetallic catalysts have lower kinetic isotope effect (KIE) values than single‐metal Pt/CNT and Ru/CNT catalysts, confirming the interaction between Pt and Ru.

**Figure 19:**
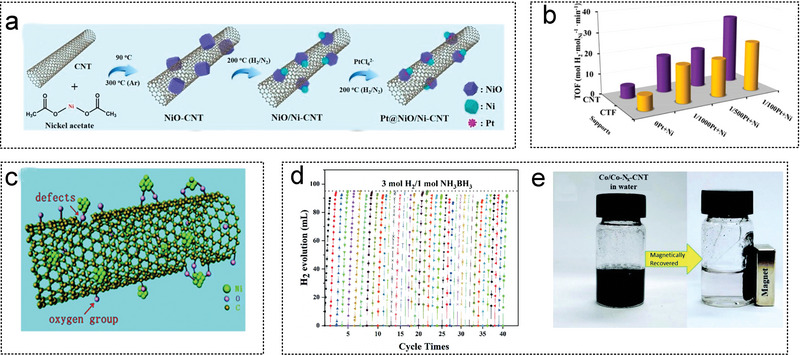
The CNTs as noble metal catalyst substrates for AB hydrolysis. a) Synthesis process of Pt@NiO/ Ni‐CNT. Reproduced with permission.^[^ ^268^ ^]^ Copyright 2019, American Chemical Society. b) Hydrogen production curves and corresponding TOF values at Pt–Ni dual sites. Reproduced with permission.^[^ ^43^ ^]^ Copyright 2017, American Chemical Society. c) Mechanism and hydrogen production activity of Ni NPs with CNTs. Reproduced with permission.^[^ ^61^ ^]^ Copyright 2015, Royal Society of Chemistry. d) Cyclic performance test of Co/ CoN*_x_*‐CNT‐33‐800T, e) and recovery diagram of catalyst adsorbed by magnet. Reproduced with permission.^[^ ^266^ ^]^ Copyright 2022, Royal Society of Chemistry.

The selective mixing of metal oxides and CNT is also a feasible method for improving activity. Liu et al. synthesized the NiO–CNT hybrid supports in a 300 °C Ar atmosphere, and then deposited Pt NPs on NiO/Ni by the H_2_ reduction method (**Figure** **19**a). The catalytic activity (2665 min^−1^) of Pt@NiO/Ni‐CNT is higher than those of Pt@Ni‐CNT and Pt@NiO‐CNT because the composition and structural characteristics of NiO/Ni can synergistically accelerate the oxidative clearance of H—OH bonds in H_2_O (rate‐determining step).^[^ ^268^ ^]^ Li et al. further deposited Pt atoms on the surface of Ni particles as a single‐atom alloy catalyst for hydrolytic dehydrogenation of AB. Minimal Pt supplementation increased activity by approximately three times. The Pt–Ni double‐site catalyst showed the highest activity for CNT and CTF supports (Figure 19b). The high activity is due to the synergistic effect of Pt and NiNPs, where the negatively charged Pt and positively charged Ni readily interact with H and OH in water, reducing the *E*_a_ of the reaction pathway.^[^ ^43^ ^]^ Sun et al. studied interfacial catalysis between CNTs and loaded Ni NPs. The C—O—Ni bond between NiNPs and thin CNTs is conducive to optimizing of the electronic structure of NiNPs and promoting the catalytic process. The hydrolysis of AB confirmed that the hybrid product with interfacial interaction had better catalytic performance (Figure 19c).^[^ ^61^ ^]^ The thickness of the CNTs was also critical for NiNPs loading. Thick CNTs had NiNPs only at the top, whereas thin CNTs had NiNPs loaded uniformly. Tsang et al. directly carbonized a mixture of a cobalt‐containing precursor, melamine, and in situ, synthesized a carbon nanotube composite composed of Co and CoN*_x_*. Co NPs were successfully embedded in the graphitic carbon layer by high‐temperature carbonization, preventing agglomeration of the NPs and the loss of catalytic activity. When the ratio of Co to melamine was 1:1.33, the reaction activity and stability of AB hydrolysis were optimal. The catalyst had excellent durability, and no significant decay was observed after 40 repeated cycles (Figure 19d).^[^ ^266^ ^]^ At the same time, the magnetism of Co itself is beneficial for the efficient recovery of the catalyst (Figure 19e).

### 2.7 Strong Coupling Effect of h‐BN

h‐BN is composed of a 2D grid of alternating sp^2^ bonds of nitrogen and boron atoms.^[^ ^269^ ^]^ The atomic surface of h‐BN is smooth, and h‐BN exhibits thermal and chemical stability, acting as an antioxidant layer even at 1100 °C. Monolayer h‐BN films remained stable in air at 800 °C. Most AB hydrolysis reactions use h‐BN‐supported non‐noble metal catalysts,^[^ ^270^ ^]^ and there are few studies on h‐BN loaded noble metals.^[^ ^227^ ^]^ Meanwhile, whether there is any interaction between B element in h‐BN and B in AB also needs to be elucidated.

**Figure 20:**
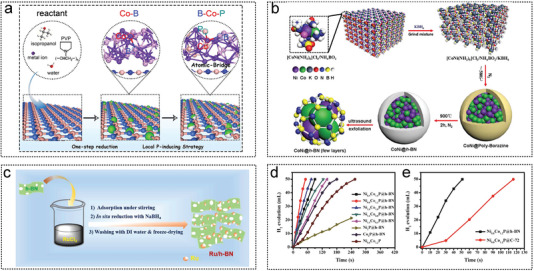
The h‐BN as metal catalyst substrates for AB hydrolysis. a) Illustration of the formation mechanism of B‐Co‐P double active site. Reproduced with permission.^[^ ^148^ ^]^ Copyright 2022, Elsevier. b) Illustration of the synthesis process at CoNi@h‐BN. Reproduced with permission.^[^ ^271^ ^]^ Copyright 2017, Elsevier. c) Illustration of Ru/h‐BN catalyst synthesis process. Reproduced with permission.^[^ ^227^ ^]^ Copyright 2021, Elsevier. d) Analysis of hydrogen generation and e) hydrogen production of different NiCoP catalysts. Reproduced with permission.^[^ ^272^ ^]^ Copyright 2019, Elsevier.

Liu et al. designed an atomic bridge structure of B‐Co‐P double active sites on h‐BN nanosheets using the sodium borohydride reduction method (**Figure** **20**a). The local phosphorus induction strategy (LPiS) is key to achieving high dynamic performance (the TOF of 37 min^−1^) and good stability. The formation of Co—B and Co—P bonds detected in XAS reveals the successful construction of B‐Co‐P atomic bridge structures under LPiS. The structure of the B‐Co‐P interface atom bridge plays a vital role in regulating the electron density of Co species and reducing the energy barrier for the reaction of AB with H_2_O molecules.^[^ ^148^ ^]^ The use of h‐BN to construct a core–shell catalyst is also an effective strategy to improve catalytic activity. Lu et al. obtained core–shell catalyst CoNi@h‐BN with CoNi alloy as the core and h‐BN as the shell by annealing bimetallic ammine boride complexes at 900 °C (Figure 20b).^[^ ^271^ ^]^ Changes in the chemical properties and electronic structure of the nucleated CoNiNPs led to improve in performance. The strong coupling between h‐BN and AB also accelerates the reaction. Porous h‐BN nanowires have been also used as ideal supports for metals. CoCu bimetallic NPs (particle size 7.2 nm) were successfully loaded onto multi‐space boron nitride nanowires (BNNFs). The high specific surface area of the BNNFs makes CuCoNPs evenly dispersed and unsuitable for agglomeration. The strong interaction between the CuCo binary metal and the h‐BN supports and CuCo metal results in enhanced catalytic activity.^[^ ^106^ ^]^ Zheng et al. successfully loaded Ru NPs onto h‐BN using an in situ adsorption reduction method. Ru NPs are the active species for the hydrolysis reaction (Figure 20c). The reaction rate increased with increasing of Ru loading, reaching a maximum at 2.0 wt%. After increasing the loading capacity, the activity did not increase because of the increase in the particle size and the decrease in the number of active sites. Therefore, 2.0 wt% Ru/h‐BN is the most suitable load.^[^ ^227^ ^]^ Support modification targeting h‐BN is also an effective means of improving the catalytic performance. Wang et al. successfully loaded NiCoP onto an h‐BN support using a hydrothermal phosphating strategy. The introduction of phosphorus further optimizes the interaction between NiCo and h‐BN, and the prepared Ni_0.8_Co_1.2_P@h‐BN showed excellent catalytic performance for the hydrogen production from boron ammonia (AB) with an initial turnover frequency of 86.5 mol^−1^ at 298 K (Figure 20d,e).^[^ ^272^ ^]^

### 2.8 Acid and Alkali Effect of Zeolites

Molecular sieves with regular nanopores are important multiphase catalytic materials, adsorption separation materials, and support materials in the industry.^[^ ^273^ ^]^ Heterogeneous catalytic reactions are carried out on solid catalysts, and their catalytic activity is related to the pore size of the catalyst. When a zeolite molecular sieve is used as a catalyst or catalyst support, the catalytic reaction is controlled by the pore size of the zeolite molecular sieve. In recent years, special molecular sieves have also been used as support metals for hydrogen production via AB hydrolysis.^[^ ^5^ ^a]^ The improvement in the catalytic performance of zeolite for AB hydrolysis is significant, but the durability of zeolite catalysts is the next factor that must be considered, as a major influence for future practical applications.

**Figure 21:**
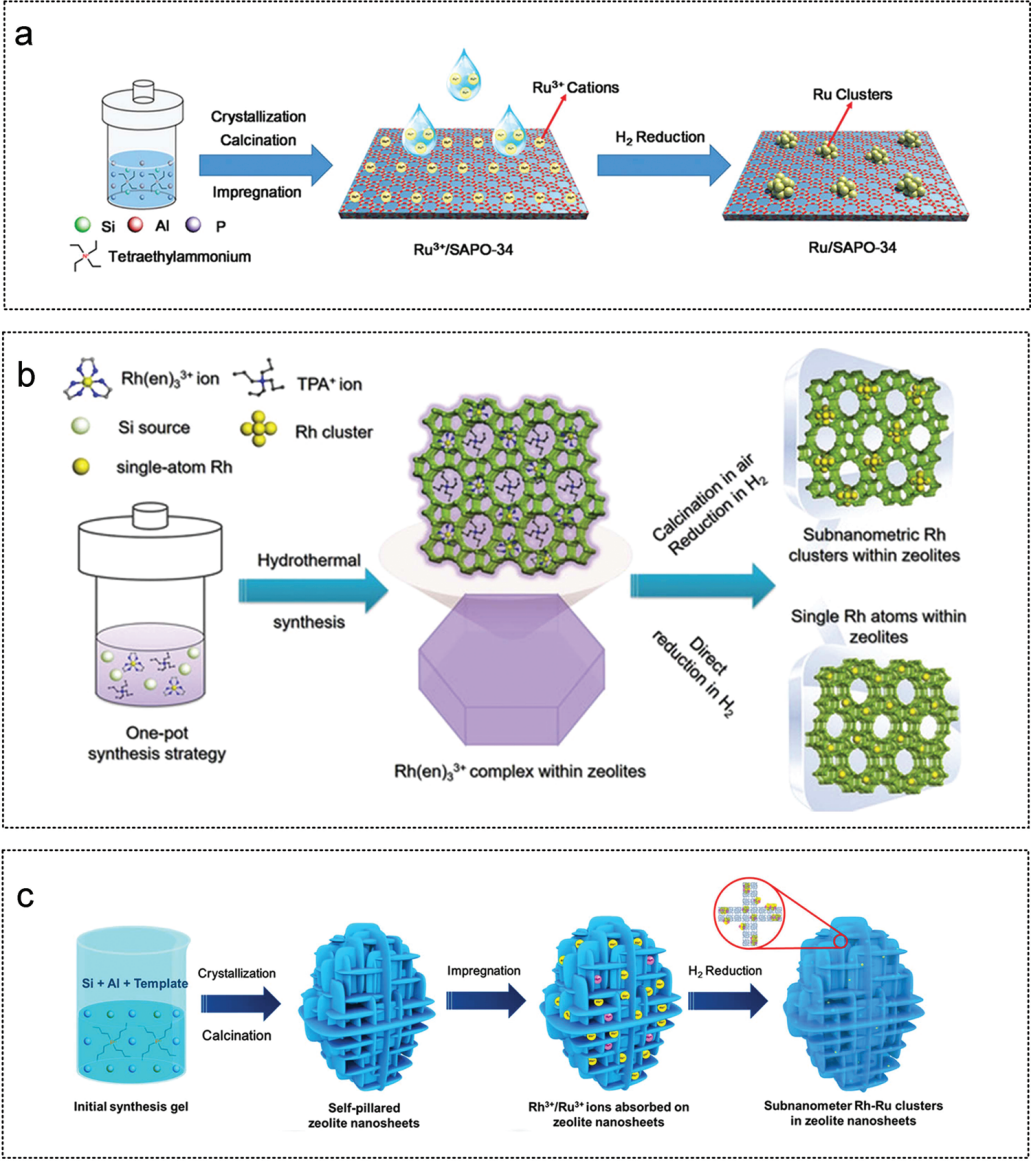
The zeolites as metal catalyst substrates for AB hydrolysis. a) Ru/SAPO‐34 catalyst synthesis process illustration. Reproduced with permission.^[^ ^195^ ^]^ Copyright 2019, Wiley‐VCH. b) Illustration and morphology characterization of single‐atom Rh catalyst synthesis anchored in MFI‐type zeolites. Reproduced with permission.^[^ ^196^ ^]^ Copyright 2019, Wiley‐VCH. c) Schematic diagram and corresponding morphology characterization of RhRu bimetal loaded on self‐pillared MFI nanosheet. Reproduced with permission.^[^ ^234^ ^]^ Copyright 2021, American Chemical Society.

Yu et al. loaded Ru nanoclusters onto silicoaluminophosphate SAPO‐34 and various aluminosilicate zeolites with tunable acidities, using a simple impregnation method. Ru clusters were uniformly dispersed in SAPO‐34 and the other zeolite supports (**Figure** **21**a). The results of the X‐ray absorption near‐side structure analysis showed that the adsorption characteristics at 22.16 KeV increased with an increase of acidity of Ru/SAPO‐34. These changes have been shown to be indicators of hydrogen adsorption on the Ru transition metal. The ultrasmall Ru clusters and adjacent Brønsted acid sites can act as bi‐functional active sites to activate NH_3_BH_3_ and H_2_O molecules and promote H_2_ formation on the surface of the zeolite. The acidity of zeolite is also an important factor affecting its activity. The TOF of Ru/SAPO‐34 reached 490 min^−1^ at 25 °C. Subsequently, Yu et al. synthesized monatomic Rh anchored in MFI‐type zeolites through hydrothermal and hydrogen reduction methods (Figure 21b).^[^ ^196^ ^]^ Compared with Rh@S1‐C synthesized by the impregnation method, Rh@S‐1‐H synthesized by the in situ reduction method did not destroy the topological structure of the zeolite molecular sieve and ensured the atomic dispersion of Rh. The transmission results clearly show the ten‐membered ring structure of the zeolite and the dispersion of single‐atom Rh. The highly dispersed monatomic Rh has the highest AB hydrolysis activity among all zeolites, with a TOF of up to 699 min^−1^. Yu et al. also synthesized Rh‐based bimetallic cluster catalysts on MIF nanosheet using the impregnation reduction method (Figure 21c). Owing to the highly dispersed effect caused by the large surface area of MIF zeolites, the particle size of the RhRu bimetallic cluster was only 0.78 nm. The formation of ultra‐small metal clusters and the interaction between metal clusters and oxygen atoms in the molecular sieve framework led to the existence of Rh in the form an oxidation state. The electron‐rich (Ru) and electron‐deficient (Rh) sites of the bimetallic Rh–Ru clusters promote the rapid activation of H_2_O and efficient H_2_ production.^[^ ^234^ ^]^

### 2.9 CDs

CDs^[^ ^226^ ^]^ have emerged as support materials in recent years, with advantages of such as diversified structure, low price, good hydrophilicity, easy doping ability (by N, B, S, and P), and nontoxicity. Their unique electronic structure and high specific surface area provide many advantages for the design and preparation of high‐performance catalysts.^[^ ^274^ ^]^ The carbon point also shows its outstanding effect in the AB hydrolysis reaction, and the next step is the appropriate development of CDs with a variety of structures and properties for hydrolysis.

**Figure 22:**
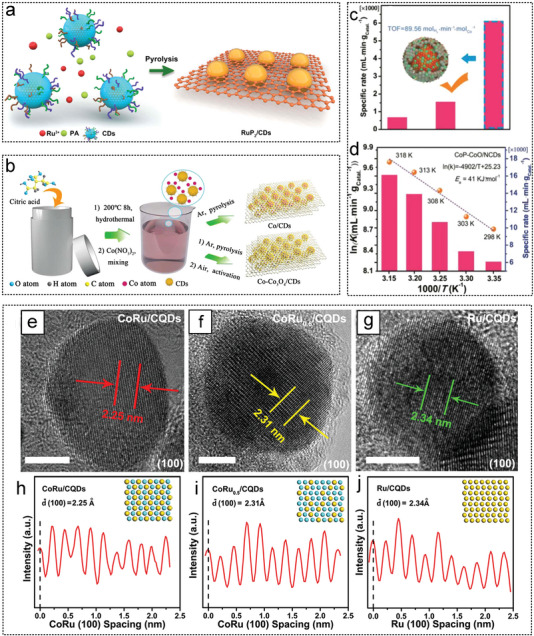
The CDs as metal catalyst substrates for AB hydrolysis. a) Illustration of RuP_2_/CDs catalyst synthesis process. Reproduced with permission.^[^ ^274^ ^]^ Copyright 2020, American Chemical Society. b) Preparation process of the Co/CDs and Co‐Co_3_O_4_/CDs catalysts. Reproduced with permission.^[^ ^122^ ^]^ Copyright 2020, Elsevier. c,d) Catalytic performance analysis of CoP‐CoO/NCDs and other comparative catalysts. Reproduced with permission.^[^ ^143^ ^]^ Copyright 2020, Elsevier. e–g) TEM characterization and h–j) corresponding lattice spacing measurements of CoRu/CQDs catalysts. Reproduced with permission.^[^ ^226^ ^]^ Copyright 2020, Wiley‐VCH.

**Figure 23:**
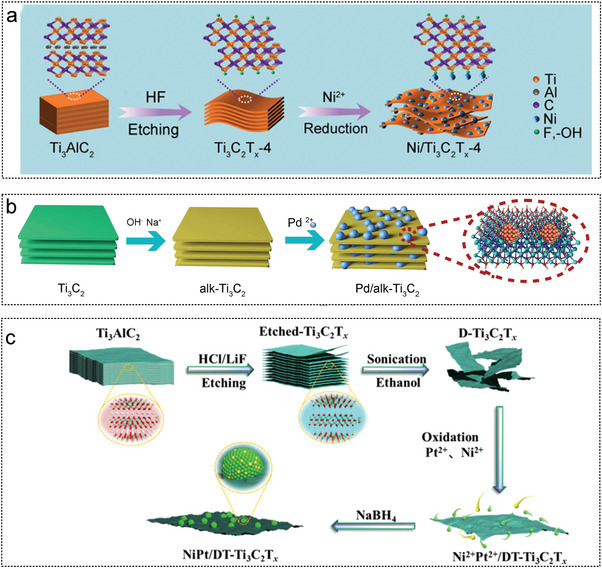
The MXene as metal catalyst substrates for AB hydrolysis. a) Illustration of Ni/Ti_3_C_2_T*_x_*‐4 catalyst synthesis process. Reproduced with permission.^[^ ^275^ ^a]^ Copyright 2022, American Chemical Society. b) Synthesis of Pd/Alk‐Ti_3_C_2_ catalyst. Reproduced with permission.^[^ ^40^ ^]^ Copyright 2021, American Chemical Society. c) Illustration of synthesis process of NiPt/DT‐Ti_3_C_2_T*_x_* catalyst. Reproduced with permission.^[277]^ Copyright 2020, Elsevier.

Liu et al. prepared RuP_2_/CDs catalysts by the physical mixing and subsequent pyrolysis of CDs, phytic acid, and ruthenium ions (**Figure** **22**a). The XRD results show RuP_2_ characteristic peaks corresponding to the PDF card JCPDS 34‐0333. The *I*_D_/*I*_G_ ratio of 1.01 confirms that the CDs are rich in defect sites. The interlayer spacing of the (110) plane of RuP_2_ was 0.39 nm. The synergistic effect between the self‐crosslinked CDs and RuP_2_ improves the catalytic performance for AB hydrolysis.^[^ ^274^ ^]^ Subsequently, Lu et al. studied the catalytic behavior of Co‐CDs catalysts for the hydrolysis of AB (Figure 22b). CDs and metal ions (Co^2+^) formed stable and uniform precursors using the ion exchange method. Then, high‐temperature annealing promotes the nucleation and CDs firing of metal particles into shells, as well as oxidative activation in air to obtain catalysts. The presence of Co, Co_3_O_4_, and self‐assembled CDs resulted in high catalyst activity. Interfacial interactions regulate the electronic structure of Co, Co_3_O_4_, and CDs to lower the activation energy barrier.^[^ ^122^ ^]^ The heterogeneous structures of Co, CoP, and nitrogen‐doped CDs exhibit strong interfacial synergistic catalytic effects on AB hydrolysis, effectively reducing the energy barrier for AB dissociation in H_2_O. Compared with Co/NCDs and Co‐Co_3_O_4_/NCDs, the CoP‐CoO/NCDs prepared by nitrogen and phosphorus co‐doping had shorter hydrogen production times (1.5 min) and higher TOF value (89.56 min^−1^). The CoP‐CoO/NCDs‐y (y = i‐v) series catalysts with similar two‐phase interface structures were obtained by controllable adjustment of phosphorus content, and all showed good catalytic activity, confirming the potential advantage of the CoP‐CoO nanoheterostructure in the hydrolysis of AB (Figure 22c,d).^[^ ^143^ ^]^ This heterogeneous interface design technique provides a new strategy for the development of efficient and inexpensive nonmetallic catalysts. Lu et al. prepared a CoRu/CQDs catalyst by combining the lattice characteristics of Ru and Co and studied the effect of lattice distortion on the catalytic activity. Lattice distortion in the CoRu alloy can be controlled by controlling the Ru content (1.0, 0.7, 0.5, 0.3, and 0.1 wt%). With the increase of Ru content, the diffraction peak of Ru changes gradually from hexagonal close‐packed Ru to hexagonal close‐packed CoRu, and the lattice spacing also changes from 2.34 nm to 2.25 nm (Figure 23e–g). The effective electron coupling of Co and Ru and the related strain effects led to faster interfacial electron transfer kinetics, and improved the catalytic performance (Figure 22h–j).^[^ ^226^ ^]^

### 2.10 Surface Functional Group Strategy of 2D Metal Carbide and Carbide (MXene)

MXene, a 2D transition metal carbide/nitride, has unique structural features, efficient charge transport properties, catalytically active substrates with exposed metal sites, and tunable surface terminations with hydrophilic properties.^[^ ^275^ ^]^ The uncertain adsorption surface functional groups of —O, —OH and —F introduced by fluorine‐containing reagents during etching can also further improve the surface characteristics of MXene. MXene has been widely studied and applied in the field of energy catalysis,^[^ ^276^ ^]^ but there have been few studies on catalytic AB hydrolysis.^[^ ^40^ ^]^ MXene has been shown to be effective in activating H_2_O. Moreover, MXene supports can effectively regulate the size, structure and electronic structure of transition metals and improve their ability to activate AB. In the future, MXene and metal atom double‐active centers can be constructed to improve the dissociation energies of NH_3_BH_3_ and H_2_O molecules.

Hou and Wu et al. improved the catalytic activity by regulating the functional groups of metal NPs catalysts (**Figure** **23**a). Ni NPs were reduced in situ on the surface of Ti_3_C_2_T*_x_* (T*_x_* = F, —OH), and the TOF of the optimized Ni/Ti_3_C_2_T_x_‐4 was 161.0 min^−1^. The NiNPs and Ti_3_C_2_T*_x_* formed a bimolecular activation channel, resulting good catalytic activity. TEM images showed that the Ni particles anchored on the surface of Ti_3_C_2_T*_x_*‐4 were uniformly dispersed without agglomeration, and the particle size was between 1.28‐5.98 nm. The lattice spacing of the (111) plane of Ni is 0.20 nm, and that of Ti_3_C_2_T*_x_*‐4 is 0.26 nm.^[^ ^275^ ^a]^ Liu and Bian et al. loaded Pd NPs with a particle size of 4.9 nm on hydroxylated Ti_3_C_2_ via methanol reduction (Figure 23b). The XRD results showed that the peak of hydroxylated Ti_3_C_2_ shifted 1.7° to a lower angle, confirming the increase in the OH^−^ promoting layer spacing. The TEM results showed that alkalized Ti_3_C_2_ exhibited stronger adsorption of Pd. The synergistic action of alkalized Ti_3_C_2_ and Pd NPs increased the catalytic activity for AB hydrolysis by 82 times, and the corresponding TOF value was 230.6 min^−1^.^[^ ^40^ ^]^ Lu et al. soaked Ni 2p and Pt 2p ions onto a DT‐Ti_3_C_2_T*_x_* surface via the coordination of hydroxyl and fluoro groups and then reduced them with NaBH_4_ to obtain a NiPt/DT‐Ti_3_C_2_T*_x_* nanocatalyst (Figure 23c). The XPS results show that the binding energy of Pt^0^ 4F in NiPt/DT‐Ti_3_C_2_T*_x_* was slightly higher than that of Pt/DT‐Ti_3_C_2_T*_x_*, confirming that Ni gains electrons from Pt. The change in the electron structure increases the electron transfer in dehydrogenation and further promotes N‐H cleavage. The best catalytic activity was obtained when Ni: Pt ratio was 4:1. Other bimetallic NPs supported by DT‐Ti_3_C_2_T*_x_* also exhibited good catalytic activity, highlighting the universality of DT‐Ti_3_C_2_T*_x_* as support. The development of heterogeneous catalysts with high selectivity and tolerance is conducive to the practical application of hydrazine borane hydrate as a promising hydrogen‐storage material.^[^ ^277^ ^]^ Fan et al. synthesized a RuCo/Ti_3_C_2_X_2_ composite by the co‐reduction of RuCl_3_ and CoCl_2_ using AB as the reducing agent and Ti_3_C_2_X_2_ thin slices as the support material. The TEM results showed that the average particle size of RuCo/ Ti_3_C_2_X_2_ was 2–3 nm. Simultaneously, using the magnetic properties of the catalyst itself can achieve efficient recovery and use of the catalyst.^[^ ^175^ ^]^ Then, Vanden et al. proposed a simple and effective method, using surface‐oxidized Ti_3_C_2_T*_x_* as NP supports, successfully loaded on O‐Ti_3_C_2_T*_x_* surface ultra‐small Rh NPs with a particle size of 2.60 nm and uniform distribution, as an excellent catalyst for the release hydrogen of AB hydrolysis. Compared with Ti_3_C_2_T*_x_* supports, the catalytic activity of O‐Ti_3_C_2_T*_x_* supports using surface oxidation is 8.21 times that of Ti_3_C_2_T*_x_* supports.^[^ ^200^ ^]^

### 2.11 Other Supports

Researchers have also systematically studied the role of other supports in AB hydrolysis. These supports are covalent organic frameworks (COF),^[^ ^278^ ^]^ Ni foams,^[^ ^147^ ^]^ cucurbit 5 (CB5),^[^ ^279^ ^]^ metal nanofilms,^[^ ^36^ ^]^ Ag@Pd nanocubes,^[^ ^212^ ^]^ TiN,^[^ ^280^ ^]^ supramolecular organic frameworks (BOFs),^[^ ^25^ ^b]^ dendrimers,^[^ ^186^ ^]^ imidazolium‐based organic polymers (IOP),^[^ ^28^ ^a]^ and polytriazolium poly(ionic liquid) (PIL).^[^ ^180^ ^]^ Finding new supports for loading transition metals is an effective way to improve activity, simultaneously, designing targeting vectors based on the factors affecting AB hydrolysis is even more important.

**Figure 24:**
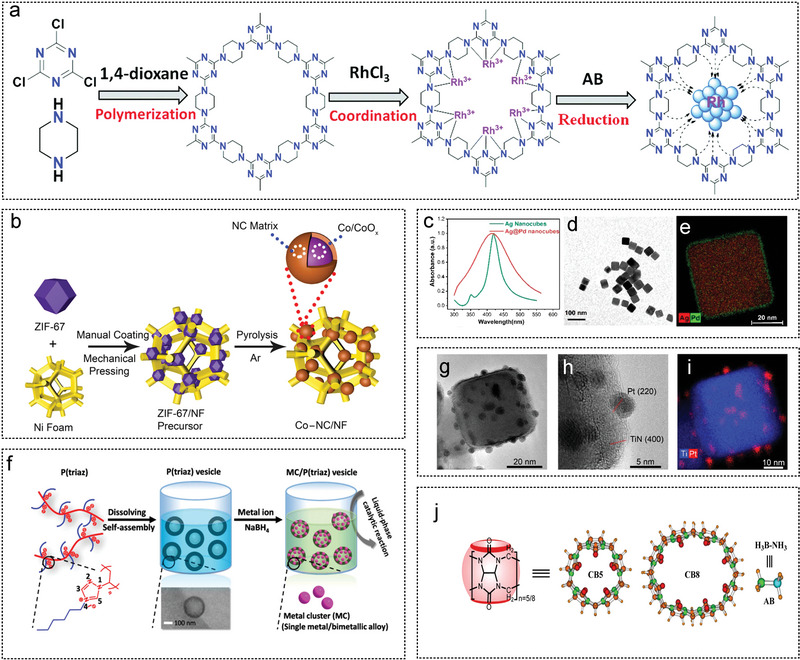
The other supports as metal catalyst substrates for AB hydrolysis. 01 a) Schematic illustration of the synthesis of the Rh/PC‐COF catalyst. Reproduced with permission.^[^ ^278^ ^]^ Copyright 2020, Royal Society of Chemistry. b) Illustration of synthesis process of Co‐NC/NF catalyst. Reproduced with permission.^[^ ^147^ ^]^ Copyright 2022, American Chemical Society. c–e) Synthesis process and corresponding morphology characterization of Ag@Pd nanocubes. Reproduced with permission.^[^ ^212^ ^]^ Copyright 2020, American Chemical Society. f) Schematic diagram of the synthesis of nanoclusters. Reproduced with permission.^[^ ^180^ ^]^ Copyright 2017, American Chemical Society. g–i) Morphology characterization of TiN‐Pt. Reproduced with permission.^[^ ^280^ ^]^ Copyright 2020, American Chemical Society. j) Structural sketches of CB5, CB8 and AB. Reproduced with permission.^[^ ^279^ ^]^ Copyright 2021, American Chemical Society.

Nitrogen‐rich covalent organic frameworks (COFs) with high nitrogen content and an inherently porous backbone support the encapsulation active metal NPs (MNPs). Lu et al. used a metal–nitrogen (M–N) coordination reduction strategy to synthesize a low‐cost nitrogen‐rich COF (PC‐COF) as a support for encapsulating Rh NPs, using piperazine and trichloroamine as raw materials. (**Figure** **24**a). The optimized Rh/PC‐COF catalyst was homogeneously dispersed with a narrow Rh nanoparticle distribution (1.4–2.6 nm) and exhibited an ultra‐high catalytic activity for the methanolysis of AB at 298 K with a TOF of 505 min^−1^.^[^ ^278^ ^]^ Li et al. prepared a Co‐NC/NF monolithic catalyst by the pyrolysis of ZIF‐67/NF precursor at a high temperature (Figure 24b).^[^ ^147^ ^]^ The highly ordered porous structure of ZIF‐67 on Ni foams inhibits the aggregation of CoNPs. The surface spacing of C (002) was 0.340 nm, confirming the presence of graphitic carbon. The integral design of catalysts based on nickel foam is necessary for practical applications. Lu et al. designed Ag@Pd core–shell nanocubes in an Ag nanomaterial core as an absorber of light and an ultrathin Pd shell as a catalytic site on the surface. TEM showed that the cubic morphology was well maintained after Pd coating on Ag the nanocubes (Figure 24c–e).^[^ ^212^ ^]^ To verify the effectiveness of the polymer, Pd was selected as an example to demonstrate the effectiveness of our polymer supports for the synthesis of high‐quality metal clusters. The particle size of the Pd nanoclusters is 1.0 nm. Using this method, Co, Ni, Cu, Ru, Rh, Ag, Pt, Au, and AuNi were synthesized in batches (Figure 24f). The catalysts exhibited high AB hydrolysis activity. This unexpectedly high catalytic activity and stability can be attributed to the capping agents.^[^ ^180^ ^]^ Naldoni et al. designed a Pd–TiN nanocrystalline hybrid, including a plasma TiN core and multiple platinum nanocrystalline catalytic centers. The apparent quantum yield of hydrogen production from ammonia boron driven by thermal electrons was 120%. TEM morphology analysis showed that Pd was evenly distributed on TiN, with little difference in particle size (Figure 24g–i).^[^ ^280^ ^]^ The catalytic activities of Ag@Pd and Pd nanocubes were very similar, and the H_2_ precipitation rate was 0.11 mmol min^‐1^, much higher than that of Ag nanocubes (0.07 mmol min^‐1^). CB‐AB complex systems, especially CB8‐AB and CB5‐AB, have more potential to be hydrogen‐producing materials at room temperature because they can improve the hydrogen release kinetics by lowering the activation barrier, significantly improve the relative H_2_ yield, and have good reusability (Figure 24j).^[^ ^279^ ^]^

**Figure 25:**
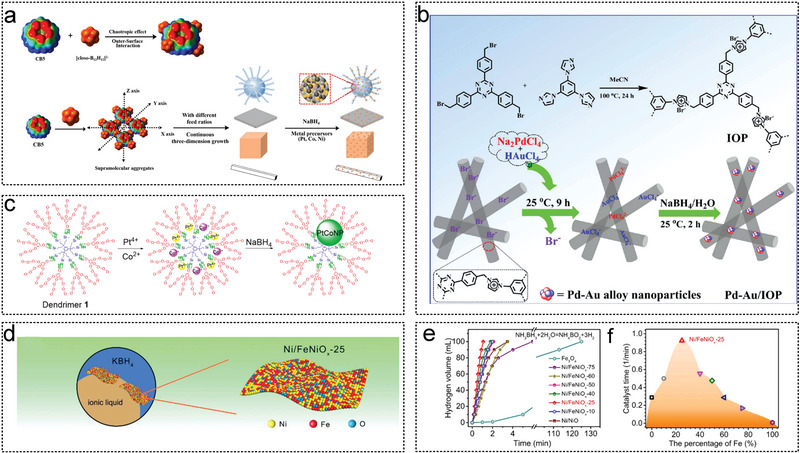
The other supports as metal catalyst substrates for AB hydrolysis. a) Schematic diagram of preparation of CB5 and in situ synthesis of trimetal PtCoNi nanoalloy. Reproduced with permission.^[^ ^25^ ^b]^ Copyright 2019, American Chemical Society. b) IOP and Pd–Au/IOP synthesis process illustration. Reproduced with permission.^[^ ^28^ ^a]^ Copyright 2018, American Chemical Society. c) Synthesis schematic of Pt‐Co/1 NPs loaded in dendrimers. Reproduced with permission.^[^ ^186^ ^]^ Copyright 2019, American Chemical Society. d) Synthesis mechanism e,f) and comprehensive analysis of hydrogen production performance for Ni/FeNiO*_x_*‐25. Reproduced with permission.^[^ ^36^ ^]^ Copyright 2021, American Chemical Society.

Through the tunable supramolecular assembly of CB5 and boron clusters, Zhou et al. successfully prepared a dodecaborate‐based supramolecular organic frameworks (BOF) based on CB5, and a trimetal PtCoNi alloy was grown in situ in this BOF (**Figure** **25**a).^[^ ^25^ ^b]^ When the molar ratio (CB5 to boron clusters) was 1, beautiful flower‐like self‐assembly structures with diameters of 400 nm were formed. Changes in the mole ratio lead to the formation of various supramolecular nanostructures. Different PtCoNi‐BOF catalysts were synthesized under the conditions of 3.5 wt% Pt loading, and the catalytic performance of AB for hydrogen release in H_2_O was investigated. The TOF value of the three‐metal PtCoNi‐BOF system was greatly improved compared to that of the single‐metal BOFs system. To verify the feasibility of imidazolium‐based organic polymers (IOP) as supports, Wang et al. synthesized Pd/IOP, Au/IOP, and PD–Au/IOP (Figure 25b).^[^ ^28^ ^a]^ Owing to partial pore filling and mass increase. This is due to partial pore filling and mass increase. The BET surface areas of Pd–Au/IOP, Pd/IOP, and Au/IOP loaded with metal NPs were 23, 20, and 21 m^2^ g^−1^, respectively. TEM results showed that the particle sizes of Pd, Au, and Pd–Au NPs in Pd/ IOP, Au/IOP, and Pd–Au/IOP were 1.55 ± 0.20,1.80 ± 0.20, and 1.50 ± 0.20 nm, respectively. The distribution and size of metal NPs are much more uniform and smaller than those of carbon, zeolites, and metal oxide supports. Dendritic macromolecules have been widely used as supports and exhibit high catalytic efficiency. Astruc et al. synthesized PtCo bimetallic catalyst by sodium borohydride reduction method combining the advantages of dendritic molecules (Figure 25c).^[^ ^186^ ^]^ The relationship between the Co content and TOF of the catalytic activity was discussed. The highest TOF value is 257.1 min^−1^ when the Co content was 50%. The synergistic effect of NaOH significantly improved the catalytic activity. Metal‐based nanocomposite films have also been used for catalytic reactions. Chen et al. synthesized a NiFe‐based nanocomposite film Ni/FeNiO*_x_*‐X by the ionic liquid/water interface method (Figure 25d).^[^ ^36^ ^]^ The Ni–Fe^2+^ dual active sites at the FeNiO*_x_* and Ni interfaces were involved in the targeted adsorption and effective activation of H_2_O and NH_3_BH_3_ molecules, respectively. The optimized Ni/FeNiO*_x_*‐25 catalyst exhibited has the best catalytic activity for AB hydrolysis, with a TOF of 72.3 min^−1^ (Figure 25e,f).

## 3 Investigation of the Catalytic Mechanism and Pathway of AB

### 3.1 Exploration of the Catalytic Mechanism

**Figure 26:**
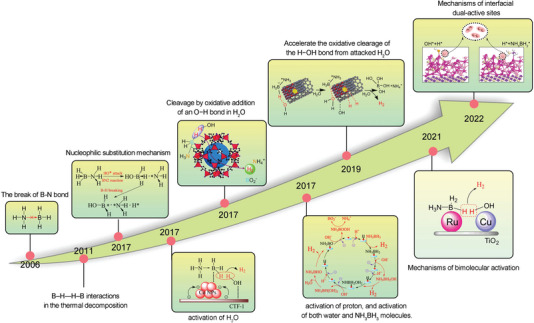
Recognition and development of catalytic mechanism.

Understanding the catalytic mechanism is crucial for catalyst design and for improving catalytic activity. **Figure** **26** depicts the progress in research on the catalytic mechanism of AB hydrolysis. The AB hydrolysis mechanism was first investigated in 2006 by Xu et al. They loaded Co‐based NPs on different supports (*γ*‐Al_2_O_3_, SiO_2,_ and C) and showed that the reduction of *E*_a_ corresponds to the breakage of the B—N bond.^[^ ^281^ ^]^ In 2014, Guan et al. confirmed that the B—H and the N—H bonds were simultaneously broken through KIE.^[^ ^282^ ^]^ In 2017, multiple catalytic mechanisms were proposed, including nucleophilic substitution,^[^ ^34^ ^]^ activated water,^[^ ^72^ ^]^ oxidation addition and reduction elimination,^[^ ^77^ ^]^ and proton activation.^[^ ^283^ ^]^ In 2019, Liu et al. suggested that oxidative clearance of the H—OH bond is the rate‐determining step.^[^ ^268^ ^]^ Li et al. proposed a bimolecular activation mechanism in 2021,^[^ ^215^ ^]^ and later suggested an interface two‐site activation mechanism in 2022.^[^ ^148^ ^]^ To date, research on the determination of the rate‐determining step (RDS) of AB remain elusive.

#### 3.1.1 Preliminary Judgment of the Activated Water Mechanism

**Figure 27:**
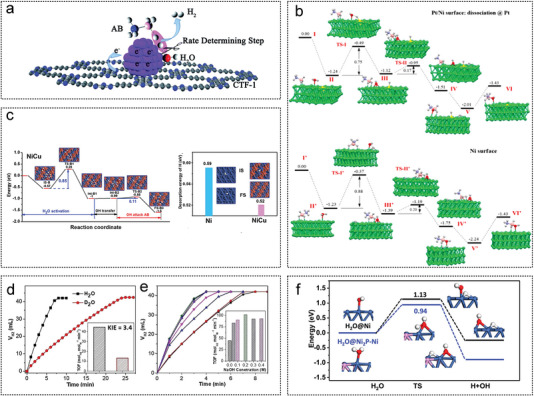
a) Illustration of water activation mechanism of Co/CTF‐1 catalyst. Reproduced with permission.^[^ ^72^ ^]^ Copyright 2017, Royal Society of Chemistry. b) Simulated hydrolytic dehydrogenation pathway at the Pt–Ni site. Reproduced with permission.^[^ ^43^ ^]^ Copyright 2017, American Chemical Society. c) Detailed process of activating water on NiCu catalyst. Reproduced with permission.^[^ ^125^ ^]^ Copyright 2020, American Chemical Society. d) Effect of H_2_O and D_2_O on hydrogen production time, e) and the influence of OH on TOF value by Cu_0.3_@Cu_0.7_CoO*_x_* @GO. Reproduced with permission.^[^ ^115^ ^]^ Copyright 2020, Elsevier. f) Dissociation of water molecules over Ni and Ni_2_P‐Ni catalysts. Reproduced with permission.^[^ ^284^ ^]^ Copyright 2019, American Chemical Society.

To determine the RDS of AB hydrolysis, Chen et al. measured the KIE with Co/CNT and Co/AC catalysts.^[^ ^72^ ^]^ The dehydrogenation behavior of NH_3_BD_3_ in H_2_O is similar to that of NH_3_BH_3_ in H_2_O. The dehydrogenation of NH_3_BH_3_ in D_2_O is slower than that in H_2_O (**Figure** **27**a). The O—H bond energy is 493 KJ mol^−1^, and the B—H bond energies are 430 KJ mol^−1^, and the B—N bond energy is 117 KJ mol^−1^. Depending on the bond energy, activated water is key, but the rupture of the B—H bond cannot be ignored. Subsequently, Chen et al. fully simulated the effect of Pt doping on the hydrolysis system (Figure 27b).^[^ ^43^ ^]^ The largest kinetic barrier in the calculation is the O—H bond breaking, requiring an energy of 0.88 eV in the existence of Ni. When Pt was added to form a Pt–Ni double site, the energy required to break the O—H bond was 0.75 eV. Thus, the active center was a single Pt atom surrounded by Ni atoms. The synergistic effect between Pt and Ni resulted in a decrease in the kinetic barrier for AB hydrolysis. Wang et al. systematically studied the reaction path of AB hydrolysis using NiCu/CNS catalysts (Figure 27c).^[^ ^125^ ^]^ Based on theoretical calculations, hydrolysis is divided into three processes: H_2_O activation, OH transfer, and OH attack AB. The electron‐rich Ni sites in NiCu can significantly promote the activation of H_2_O. Kinetic isotope effects with KIE values of 2.8 indicate bonding or breaking with isotopically labeled atoms in RDS. The KIE value calculated for the Cu_0.3_@Cu_0.7_CoO*_x_* @GO catalyst was 3.4 (Figure 27d). This evidence suggests that the cleavage of the O—H bond from H_2_O is the rate‐determining step for AB hydrolysis (Figure 27e).^[^ ^115^ ^]^ Song et al. simulated the reaction pathway for activating H_2_O molecules during AB hydrolysis (Figure 27f).^[^ ^284^ ^]^ The low energy barrier 0.94 eV of the Ni/Ni_2_P surface indicates that the heterostructures have suitable properties. The formation of the Ni—P bonds and the transfer of electrons from Ni to Ni_2_P effectively promoted the activation of H_2_O. The synergistic effects of C, Ni, and Ni/Ni_2_P in the heterostructure complex simultaneously promoted the adsorption activation of the reactants and the formation of the final products during AB hydrolysis.

#### 3.1.2 Oxidation‐Reduction Mechanisms (Oxidative Addition and Reductive Elimination)

**Figure 28:**
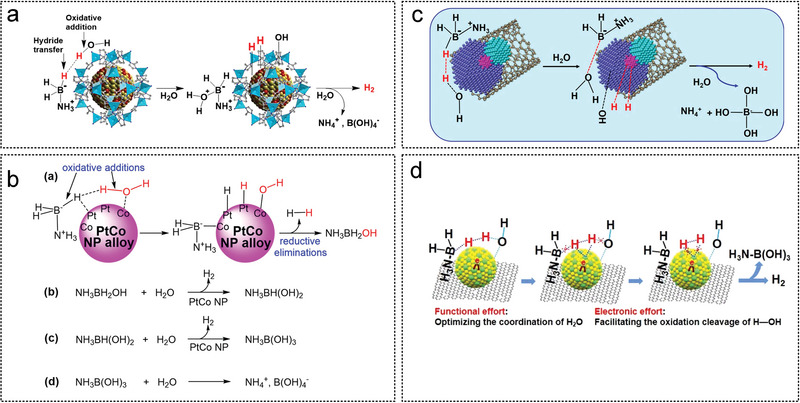
a) AB hydrolysis mechanism by NiPt@ZIF‐8 catalyst. Reproduced with permission.^[^ ^18^ ^]^ Copyright 2018, American Chemical Society. b) Simulated catalytic pathways by PtCo catalysts. Reproduced with permission.^[^ ^186^ ^]^ Copyright 2019, American Chemical Society. c) Oxidation cleavage of H—OH bond in H_2_O by Pt@NiO/Ni‐CNT catalyst. Reproduced with permission.^[^ ^268^ ^]^ Copyright 2019, American Chemical Society. d) Simulation of catalytic mechanisms by RhNi@NHMCs. Reproduced with permission.^[^ ^237^ ^]^ Copyright 2021, American Chemical Society.

Didier et al. proposed an oxidation addition mechanism for AB hydrolysis (**Figure** **28**a).^[^ ^18^ ^]^ A high KIE value (4.95) confirms the oxidative addition of the O—H bond during the rate‐determining step. B—H bond exhibits hydride characteristics and promotes hydrogen transfer. H_3_NBH_2_H and H_2_O formed hydrogen bonds [H_3_NBH_2_H] ···H—OH, eventually releasing a molecule of H_2_. Subsequently, Didier et al. further studied the reduction elimination generation process of H_2_ (Figure 28b).^[^ ^186^ ^]^ After oxidation addition, Co adsorbed NH_3_BH_2_ and O—H, and the two surrounding Pt atoms adsorbed H from the B—H bond fracture, and O—H bond fracture, respectively. The reduction elimination of H_2_ is formed from two H atoms on Pt, and the intermediate NH_3_BH_2_OH is formed from NH_3_BH_2_^−^ and OH^−^. Liu et al. further analyzed the oxidation cleavage of H—OH bond in H_2_O using a Pt@NiO/Ni‐CNT catalyst (Figure 28c).^[^ ^268^ ^]^ On the one hand, the presence of NiO is beneficial for the adsorption of H—OH and the dissociation of positive H. On the other hand, the electron‐rich surface state of Pt in the NiPt alloy facilitates the interaction with H. Both AB and H_2_O molecules can kinetically dissociate positively charged H on electron‐rich Pt surfaces. Two positively charged H ions then covalently bond to form H_2_ molecules, with the H_2_ molecules immediately released from the Pt surface. The mechanism analysis of the RhNi@NHMCs catalyst shows that adding more Rh on the Ni surface facilitates the coordination of H_2_O on Ni surface, thus promoting the oxidation cleavage of the O—H bond in H_2_O (Figure 28d).^[^ ^237^ ^]^ Ni alloying can change the electronic structure of the Rh surface and thermodynamically reduce its *E*_a_.

#### 3.1.3 Nucleophilic Substitution Mechanism

**Figure 29:**
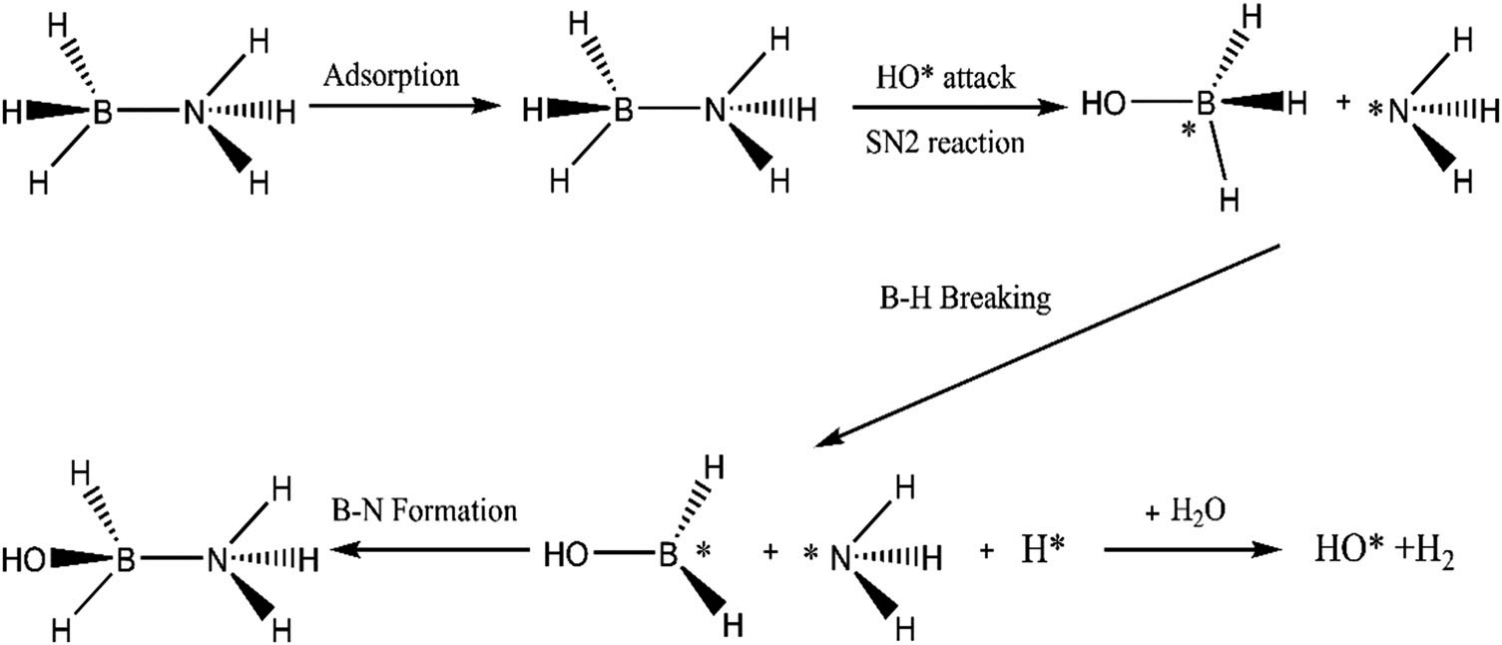
S_N_2 catalytic mechanism of AB hydrolysis. Reproduced with permission.^[^ ^34^ ^]^ Copyright 2017, Royal Society of Chemistry.

Nucleophilic substitution usually occurs on a positively or partially positively charged carbon, where the carbon atom reacts with a negatively or partially negatively charged nucleophilic reagent and is substituted. Nucleophilic substitution reaction (S_N_2) was used to explain the reaction mechanism of AB hydrolysis. The detailed process is shown in **Figure** **29**, where the reaction is initiated by AB and H_2_O adsorbed on the surface of the catalyst. According to the S_N_2 reaction mechanism, HO* attacks the B bond in BH_3_NH_3_, replaces the B—H bond, and releases H*(OH* + BH_3_NH_3_*→BH_3_OH* + NH_3_*).^[^ ^34^ ^]^ The S_N_2 step is the rate‐limiting step in the hydrolytic dehydrolysis of AB, which reasonably explains why the presence of OH can significantly improve the catalytic performance and shorten the induction period.

#### 3.1.4 Bimolecular Activation of AB and Water

**Figure 30:**
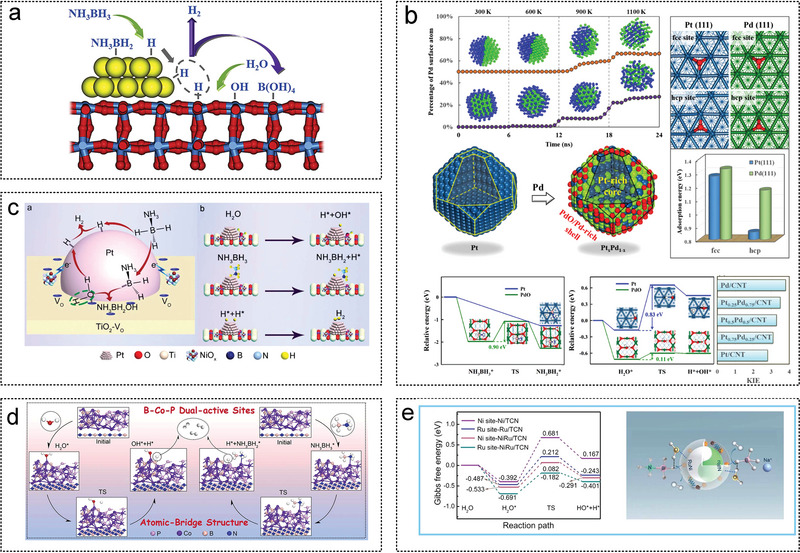
a) Schematic diagram of Pt‐WO_3_ dual site activation mechanism. Reproduced with permission.^[^ ^199^ ^]^ Copyright 2020, Elsevier. b) Diagram of PtPd bimetal structure, and potential energy diagram of activated AB and water molecules. Reproduced with permission.^[^ ^28^ ^b]^ Copyright 2020, American Chemical Society. c) Dual activation mode of Pt and oxygen vacancy. Reproduced with permission.^[^ ^231^ ^]^ Copyright 2021, Wiley‐VCH. d) B‐Co‐P double site activation mode by Co_3_B‐CoP/h‐BN catalyst. Reproduced with permission.^[^ ^148^ ^]^ Copyright 2022, Elsevier. e) The *E*_a_ of water molecules is at different sites of the Ni_1_Ru_1_/TCN catalyst. Reproduced with permission.^[^ ^216^ ^]^ Copyright 2021, Elsevier.

**Figure 31:**
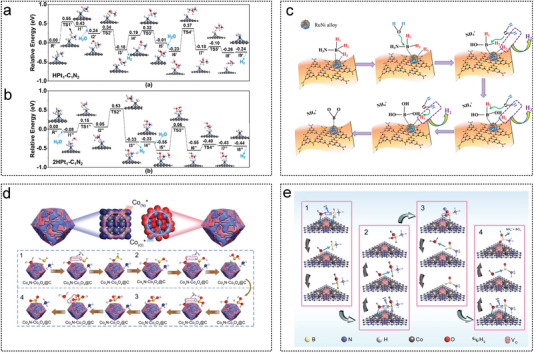
The reaction path of the hydrolysis of NH_3_BH_3_ on a) HPt_1_‐C_1_N_2_ and b)2HPt_1_‐C_1_N_2_. Reproduced with permission.^[^ ^285^ ^]^ Copyright 2022, American Chemical Society. c) The possible reaction path for AB hydrolysis by RuNi/p‐g‐C_3_N_4_ catalyst. Reproduced with permission.^[^ ^27^ ^a]^ Copyright 2020, American Chemical Society. d) Oxygen vacancy‐relating catalytic pathways by Co‐CN‐O‐100 catalyst. Reproduced with permission.^[^ ^109^ ^]^ Copyright 2022, Wiley‐VCH. e) Hydrolysis pathways at double active sites Co_(N)_* and Co_(O)_*. Reproduced with permission.^[^ ^149^ ^]^ Copyright 2020, Elsevier.

The mechanism of bimolecular activation of AB and water has been studied. Ammonia boron was hydrolyzed at the Pt‐WO_3_ double‐active site (**Figure** **30**a).^[^ ^199^ ^]^ Pt NPs promoted the decomposition of NH_3_BH_3_ into NH_3_BH_2_ and H, and WO_3_ promoted the decomposition of H_2_O into OH and H. The H atoms at the metal‐oxide interface combine to form H_2_. Subsequently, Duan et al. synthesized a Pt–PdO interface double‐site catalyst (Figure 30b).^[^ ^28^ ^b]^ Surface Pd atoms are more likely to adsorb and bind to oxygen during catalyst passivation. Compared with PdO, Pt has a stronger ability to activate AB, whereas H_2_O can achieve rapid dissociation on PdO. The Pt‐rich nucleus and PdO‐Pd shell can be used as double catalytic sites for ammonia to promote dehydrogenation of AB and H_2_O. In catalytic dehydrogenation, oxygen vacancies can act sites (Figure 31c).^[^ ^231^ ^]^ H_2_O was adsorbed by the O—H bond to V_O1_ near the Pt atom. The O—H bond then breaks, forming Pt‐H* and V_O1_‐OH*. NH_3_BH_3_ is adsorbed on Pt atoms through B and decomposed into Pt‐H* and Pt‐NH_3_BH_2_*. V_O1_‐OH* combined with Pt‐NH_3_BH_2_* to form NH_3_BH_2_OH. The two Pt‐H* atoms then combine on the Pt surface to form the first H_2_ molecule. The B‐Co‐P double site activation mode was also studied in depth by Li et al. (Figure 30d).^[^ ^148^ ^]^ NH_3_BH_3_ and H_2_O molecules were adsorbed on the surface of Co_3_B‐CoP/h‐BN by the atomic bridge structure of B‐Co‐P double active site, respectively. H_2_O‐[Co‐B]* dissociated more easy into OH‐[Co‐B]* and H[Co‐B]*. The B—H bond in NH_3_BH_3_[Co‐P]* breaks to form NH_3_BH_2_[Co‐P] * and H‐[Co‐P]*. Subsequently, H‐[Co‐P]* and H‐[Co‐B]* release H_2_ molecules from the surface of the active catalyst, and OH‐[Co‐B] * attacks NH_3_BH_2_[Co‐P] * to form NH_3_BH_2_OH*. Guo et al. believed that a hydrogen bond ([H_3_NBH_2_H]···H‐OH) between NH_3_BH_3_ and H_2_O was formed during the hydrolysis process because of the hydride nature of B—H bond (Figure 30e).^[^ ^216^ ^]^ When NH_3_BH_3_ and H_2_O molecules are near the NiRu/TCN surface, the electron‐rich Ru atom is most likely to activate the B—H bond in the molecule, whereas the electron‐less Ni atom is most likely to activate the O—H bond in the water molecule. The two activated H atoms then combined to form H_2_ molecules.

### 3.2 Exploration of Catalytic Pathways

The detailed study and prediction of the AB hydrolysis pathway have practical significance for understanding the catalytic mechanism and improving the catalytic activity. Wu et al. simultaneously reserve one hydrogen atom and two hydrogen atoms on Pt_1_‐C_1_N_2_ for studying the catalytic process of AB hydrolysis (**Figure** **31**a).^[^ ^285^ ^]^ The complete hydrolysis of AB on HPt_1_‐C_1_N_2_ began with the separation of hydrogen from the adsorbed AB (TS1′) at an energy barrier of 0.55 eV. The first H_2_O molecule (I2′) then attacks *BH_2_NH_3_ to form (I3′) with a lower energy barrier of 0.10 eV. The resulting molecules adsorbed H_2_ with a desorption energy of 0.37 eV. *BH_2_(OH)NH_3_ (I4′) releases a second hydrogen barrier of 0.13 eV, forming *BH(OH)NH_3_ (I5′). The second H_2_O molecule attacked *BH(OH)NH_3_ (I6′) to form *BH(OH)(H_2_O)NH_3_ (I7′), corresponding to an energy barrier of 0.60 eV. In the next step, *BH(OH)(H_2_O)NH_3_ (I7′) dissociates to form an adsorbed hydrogen molecule and *BH(OH)_2_NH_3_ (I8′). The H_2_ desorption energy generated in the second step is 0.68 eV, which is the RDS of the whole hydrolysis process. The RDS may shift to the formation of *BH(OH)(H_2_O)NH_3_ with an entire barrier of 0.60 eV if the entropy of gas‐phase hydrogen is considered. Separate H atom is difficult from BH_3_NH_3_ at the 2HPt_1_‐C_1_N_2_ site, and the reaction energy is 1.63 eV (Figure 31b). On the contrary, the direct introduction of the H_2_O molecule (I1) effectively lowers the reaction barrier at the initial step to 0.33 eV, forming I_2_." Zheng et al. simulated this catalytic pathway (Figure 31c).^[^ ^27^ ^a]^ First, the interaction between AB and the RuNi alloy NPs surface promotes the activation of the B—H bond in AB, forming active RuNi‐H species. The AB‐RuNi intermediate species were then attracted to the H_2_O molecules adsorbed on the surface of the RuNi NPs, breaking the B—N bond. Then, H in the BH_3_ intermediate and H in H_2_O form H_2_. The reaction pathways for bimolecular activation have also been studied thoroughly.^[^ ^147^, ^286^ ^]^ Li et al. simulated the specific reaction path on Co_3_O_4_ nanocrystals loaded with oxygen‐rich vacancies on C_3_N_4_ (Figure 31d),^[^ ^109^ ^]^ and this reaction can be divided into five steps as follows.

(9) $${\mathrm{NH}}_{3}{\mathrm{BH}}_{3}+H_{2}O\to {\mathrm{NH}}_{3}{\mathrm{BH}}_{2}\mathrm{OH}+H_{2}$$

(10) $${\mathrm{NH}}_{3}{\mathrm{BH}}_{2}\mathrm{OH}+H_{2}O\to {\mathrm{NH}}_{3}\mathrm{BH}{\left(\mathrm{OH}\right)}_{2}+H_{2}$$

(11) $${\mathrm{NH}}_{3}\mathrm{BH}{\left(\mathrm{OH}\right)}_{2}\to {\mathrm{NH}}_{3}\mathrm{BHO}+H_{2}O$$

(12) $${\mathrm{NH}}_{3}\mathrm{BHO}+H_{2}O\to {\mathrm{NH}}_{3}\mathrm{BOOH}+H_{2}$$

(13) $${\mathrm{NH}}_{3}\mathrm{BOOH}\to {\mathrm{NH}}_{4}^{-}+{\mathrm{BO}}_{2}^{-}$$

In Equation (11), the two hydroxyl groups in NH_3_BH(OH)_2_ condense to remove a molecule of H_2_O. Subsequently, Li designed Co_(N)_* and Co_(O)_* double active sites and combined the double active sites with AB and H_2_O molecules to analyze the reaction path (Figure 31e).^[^ ^149^ ^]^ The specific reaction path is as follows:

(14) $${\mathrm{NH}}_{3}{\mathrm{BH}}_{3}+{\mathrm{Co}}_{\left(N\right)}^{*}={\mathrm{NH}}_{3}{\mathrm{BH}}_{2}-{\mathrm{Co}}_{\left(N\right)}^{*}+H^{*}$$

(15) $$H_{2}O+{\mathrm{Co}}_{\left(O\right)}^{*}=\mathrm{HO}-{\mathrm{Co}}_{\left(O\right)}^{*}+H^{*}$$

(16) $$\begin{array}{ccc} & & {\mathrm{NH}}_{3}{\mathrm{BH}}_{2}-{\mathrm{Co}}_{\left(N\right)}^{*}+H^{*}+\mathrm{HO}-{\mathrm{Co}}_{\left(O\right)}^{*}+H^{*}\\ & & \;=\mathrm{HO}-{\mathrm{Co}}_{\left(O\right)}^{*}+{\mathrm{NH}}_{3}{\mathrm{BH}}_{2}\mathrm{OH}-{\mathrm{Co}}_{\left(N\right)}^{*}+H_{2}↑ \end{array}$$

(17) $$\begin{array}{ccc} & & {\mathrm{NH}}_{3}{\mathrm{BH}}_{2}\mathrm{OH}-{\mathrm{Co}}_{\left(N\right)}^{*}+\mathrm{HO}-{\mathrm{Co}}_{\left(O\right)}^{*}+H^{*}\\ & & \;={\mathrm{NH}}_{3}\mathrm{BH}{\left(\mathrm{OH}\right)}_{2}-{\mathrm{Co}}_{\left(N\right)}^{*}+H_{2}↑ \end{array}$$

(18) $$\begin{array}{ccc} & & {\mathrm{NH}}_{3}\mathrm{BH}{\left(\mathrm{OH}\right)}_{2}-{\mathrm{Co}}_{\left(N\right)}^{*}+\mathrm{HO}-{\mathrm{Co}}_{\left(O\right)}^{*}+H^{*}\\ & & \;={\mathrm{NH}}_{3}B{\left(\mathrm{OH}\right)}_{3}-{\mathrm{Co}}_{\left(N\right)}^{*}+H_{2}↑ \end{array}$$

(19) $${\mathrm{NH}}_{3}B{\left(\mathrm{OH}\right)}_{3}-{\mathrm{Co}}_{\left(N\right)}^{*}={\mathrm{NH}}_{3}\mathrm{BOOH}-{\mathrm{Co}}_{\left(N\right)}^{*}+H_{2}O$$

(20) $${\mathrm{NH}}_{3}\mathrm{BOOH}-{\mathrm{Co}}_{\left(N\right)}^{*}\to {\mathrm{NH}}_{4}^{+}+{\mathrm{BO}}_{2}^{-}$$

At the same time, there is also the possibility that OH polycondensation into H_2_O occurs after the complete release of three molecules of H_2_; that is, B(OH)_3_ is converted into BOOH and H_2_O at the Co_(N)_* site.

**Figure 32:**
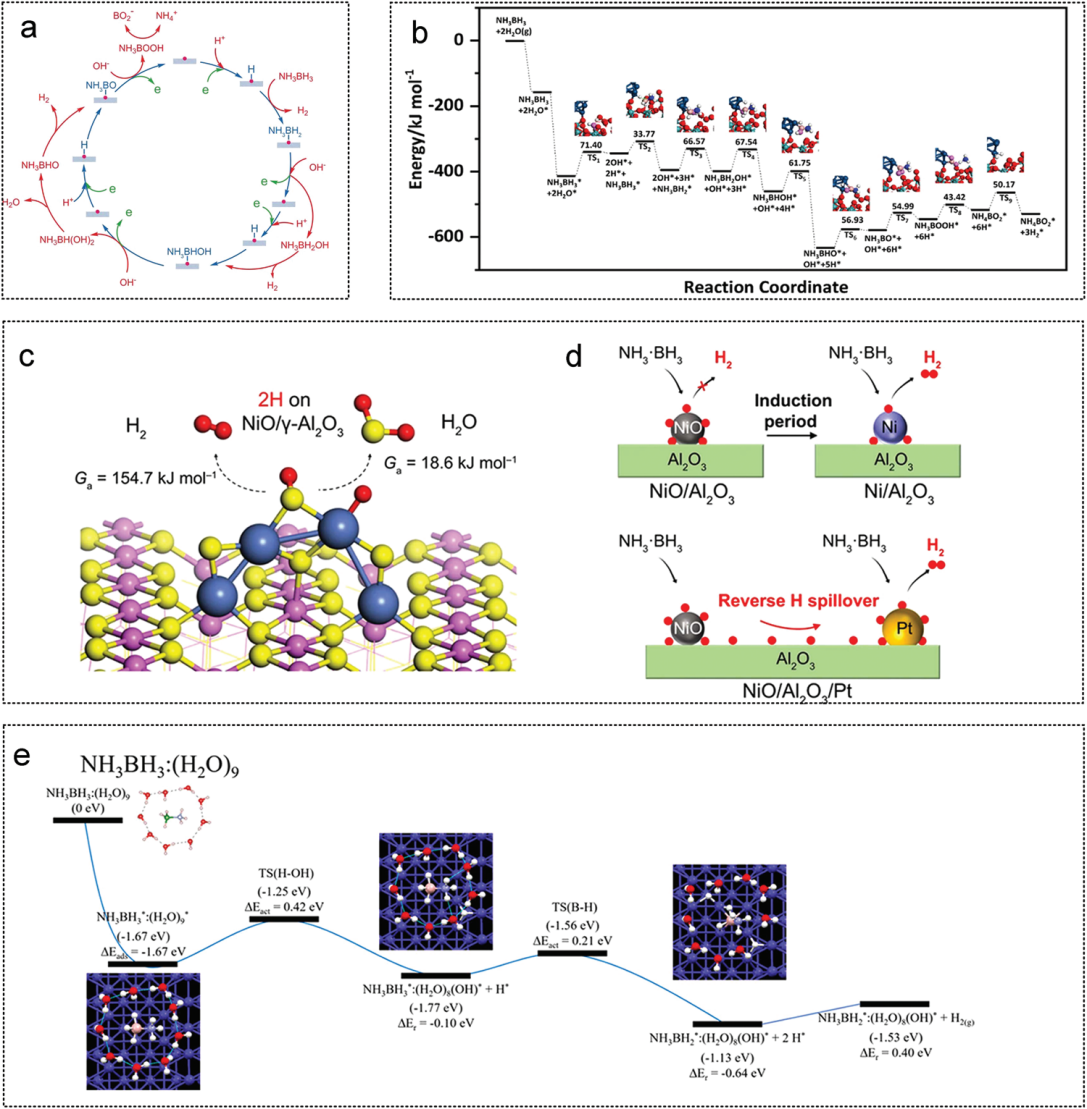
a) Illustration of proton activation pathways. Reproduced with permission.^[^ ^283^ ^]^ Copyright 2022, Wiley‐VCH. b) AB hydrolysis pathway based on metal−support synergistic. Reproduced with permission.^[^ ^257^ ^]^ Copyright 2022, American Chemical Society. c) The free energy barriers (*G*_a_) for the formation and d) desorption of H_2_ and H_2_O on NiO/Al_2_O_3_/Pt catalyst. Reproduced with permission.^[^ ^256^ ^]^ Copyright 2022, Springer Nature. e) Proposed mechanisms for formation of H_2_. Reproduced with permission.^[15]^ Copyright 2021, American Chemical Society.

Zeng et al. first proposed a proton activation pathway by examining the dependence of the reaction rate on the NH_3_BH_3_ concentration or proton concentration (pH). The detailed reaction path is as follows (**Figure** **32**a):

(21) H+e+∗→H∗

(22) $${\mathrm{NH}}_{3}{\mathrm{BH}}_{3}+H*\to H_{2}+{\mathrm{NH}}_{3}{\mathrm{BH}}_{2}*$$

(23) $${\mathrm{NH}}_{3}{\mathrm{BH}}_{2}*+{\mathrm{OH}}^{-}\to {\mathrm{NH}}_{3}{\mathrm{BH}}_{2}\mathrm{OH}+e+*$$

(24) $${\mathrm{NH}}_{3}{\mathrm{BH}}_{2}\mathrm{OH}+H*\to H_{2}+{\mathrm{NH}}_{3}\mathrm{BHOH}*$$

(25) $${\mathrm{NH}}_{3}\mathrm{BHOH}*+{\mathrm{OH}}^{-}\to {\mathrm{NH}}_{3}\mathrm{BH}{\left(\mathrm{OH}\right)}_{2}+e+*$$

(26) $${\mathrm{NH}}_{3}\mathrm{BH}{\left(\mathrm{OH}\right)}_{2}\to {\mathrm{NH}}_{3}\mathrm{BHO}+H_{2}O$$

(27) $${\mathrm{NH}}_{3}\mathrm{BHO}+H*\to H_{2}+{\mathrm{NH}}_{3}\mathrm{BO}*$$

(28) $${\mathrm{NH}}_{3}\mathrm{BO}*+{\mathrm{OH}}^{-}\to {\mathrm{NH}}_{3}\mathrm{BOOH}+e+*$$

(29) $${\mathrm{NH}}_{3}\mathrm{BOOH}\to {\mathrm{NH}}_{4}^{+}+{\mathrm{BO}}_{2}^{-}*$$

Wei et al. experimentally confirmed that the electron‐deficient Pt^2+^ site activates the B—H bond, while the O_v_‐Mo^5+^ site activates the O—H bond. The catalytic reaction pathway was simulated based on theoretical calculations (Figure 32b).^[^ ^257^ ^]^ The detailed reaction path is as follows.

(30) $${\mathrm{NH}}_{3}{\mathrm{BH}}_{3}+2H_{2}O\left(g\right)\to {\mathrm{NH}}_{3}{\mathrm{BH}}_{3}+2H_{2}O*$$

(31) $${\mathrm{NH}}_{3}{\mathrm{BH}}_{3}+2H_{2}O*\to {\mathrm{NH}}_{3}{\mathrm{BH}}_{3}*+2H_{2}O*$$

(32) $${\mathrm{NH}}_{3}{\mathrm{BH}}_{3}*+2H_{2}O*\to 2\mathrm{OH}*+2H*+{\mathrm{NH}}_{3}{\mathrm{BH}}_{3}*$$

(33) $$2\mathrm{OH}*+2H*+{\mathrm{NH}}_{3}{\mathrm{BH}}_{3}*\to 2\mathrm{OH}*+3H*+{\mathrm{NH}}_{3}{\mathrm{BH}}_{2}*$$

(34) $$2\mathrm{OH}*+3H*+{\mathrm{NH}}_{3}{\mathrm{BH}}_{2}*\to {\mathrm{NH}}_{3}{\mathrm{BH}}_{2}\mathrm{OH}*+\mathrm{OH}*+3H*$$

(35) $${\mathrm{NH}}_{3}{\mathrm{BH}}_{2}\mathrm{OH}*+\mathrm{OH}*+3H*\to {\mathrm{NH}}_{3}\mathrm{BHOH}*+\mathrm{OH}*+4H*$$

(36) $${\mathrm{NH}}_{3}\mathrm{BHOH}*+\mathrm{OH}*+4H*\to {\mathrm{NH}}_{3}\mathrm{BHO}*+\mathrm{OH}*+5H*$$

(37) $${\mathrm{NH}}_{3}\mathrm{BHO}*+\mathrm{OH}*+5H*\to {\mathrm{NH}}_{3}\mathrm{BO}*+\mathrm{OH}*+6H*$$

(38) $${\mathrm{NH}}_{3}\mathrm{BO}*+\mathrm{OH}*+6H*\to {\mathrm{NH}}_{3}{\mathrm{BO}}_{2}*+3H_{2}*$$

The Pt^2+^‐O_v_‐Mo^5+^ interface site, which is the intrinsic active center, plays a key role in promoting the hydrolytic dehydrogenation of AB. Qin et al. studied the reverse spillover mechanism of spatially separated NiO/Al_2_O_3_/Pt catalyst (Figure 32c,d).^[^ ^256^ ^]^ In the process of AB hydrolysis, there is no metal Ni^0^ in the NiO/Al_2_O_3_/Pt catalyst, but the reduced of NiO exists in the NiO/Al_2_O_3_ catalyst. The reduction of NiO is completely inhibited by the addition of Pt to the NiO/Al_2_O_3_/Pt catalyst, indicating that the H species produced at the NiO sites are not required for the reduction of NiO. Species H overflows from NiO to the Pt site, where it binds to H_2_ and is released. Lu et al. studied the influence of hydrogen‐bonding interactions in H_2_O clusters on the mechanism and path of AB hydrolysis (Figure 32e).^[^ ^15^ ^]^ The adsorption energies of the AB and (H_2_O)_9_ surface are Δ*E*_ads_ of −1.67 eV. The amino group of AB tends to interact with the hydrogen bond associated with H_2_O after the cleavage of H—OH. The synergistic action of AB and the hydrogen bonds in water further promotes the dissociation of H_2_O molecules. Rupture of the B—H bond in [Co‐(NH_3_BH_3_:(H_2_O)_8_(OH))*] leads to the formation of an intermediate in [Co‐(NH_3_BH_2_:(H_2_O)_8_(OH))*]. Subsequently, the combination of two adjacent H* atoms result in the formation of H_2_(g).

## 4 Green and Sustainable Recycling Technology of AB

AB contains hydrogen and protic hydrogen, facilitating the release of H_2_ under mild conditions. Highly efficient catalysts promote H_2_ production. However, the key to practical application is effective recyclability. Currently, little research and reporting have been done in this area, and further research is needed.^[^ ^287^ ^]^ At present, AB is mainly recycled and regenerated through the hydrazine and liquid ammonia, hydrodechlorination and lithium aluminum hydride methods.

### 4.1 Three Ways of Regenerating AB from Dehydrogenation Byproducts

#### 4.1.1 Regeneration of AB Using Hydrazine and Liquid Ammonia

**Figure 33:**
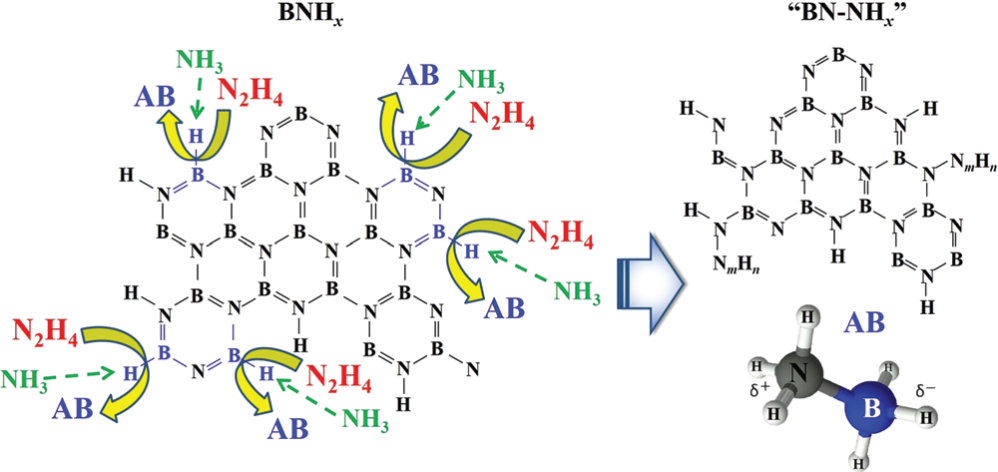
Schematic diagram of regenerating AB from hydrazine and liquid ammonia.Reproduced with permission.^[^ ^288^ ^]^ Copyright 2020, MDPI.

The regeneration of AB from hydrazine and liquid ammonia is currently the most studied method. The release of hydrogen from AB is accompanied by the formation of a heterogeneous solid boron borohydride material (BNH*_x_*). The highly polymerized material was mainly composed of (BH_2_NH_2_)*_x_* and (BHNH)*_x_*, and its structural characteristics were similar to those of polyborazene (PB).^[^ ^289^ ^]^ Hydrazine and liquid ammonia attack the B—H bond at the edge of BNH*_x_* to form an N_m_H_n_ bond, resulting in the release of AB and N_m_H_n_ from the BNH*_x_* surface (**Figure** **33**). However, the regeneration process still needs to overcome the following difficulties: first, the reaction must be carried out under a high pressure and sealed environment, and second, measures must to be taken to increase the amount of B—H in BNH*_x_* to increase the yield of AB.^[^ ^288^ ^]^

#### 4.1.2 Regeneration of AB by Hydrodechlorination

**Figure 34:**
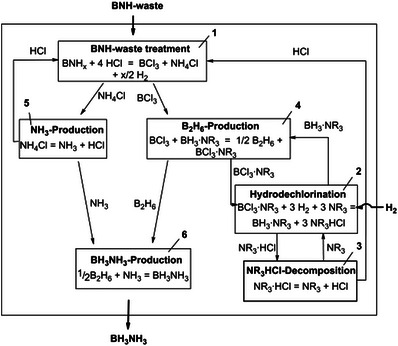
Schematic diagram of AB regeneration by hydrodechlorination. Reproduced with permission.^[^ ^290^ ^]^ Copyright 2008, Elsevier.

As shown in **Figure** **34**, hydrodesulfurization of BNH*_x_*, the pyrolysis product of AB, was performed in the following four steps (39–42).^[^ ^290^ ^]^

(39) $${\mathrm{BNH}}_{x}+4\mathrm{HCl}\to {\mathrm{BCl}}_{3}+{\mathrm{NH}}_{4}\mathrm{Cl}+x/2H_{2}$$

(40) $${\mathrm{NH}}_{4}\mathrm{Cl}\to {\mathrm{NH}}_{3}+\mathrm{HCl}$$

(41) $${\mathrm{BCl}}_{3}+3H_{2}\to 2B_{2}H_{6}+3\mathrm{HCl}$$

(42) $$4{\mathrm{NH}}_{3}+2B_{2}H_{6}\to {\mathrm{NH}}_{3}{\mathrm{BH}}_{3}$$

BNH*_x_* can be completely digested using hydrochloric acid to obtain BCl_3_ and NH_4_Cl (32), and NH_4_Cl can be further transformed into NH_3_+HCl (33). The rational design of the BCl_3_ hydrodechlorination process is key to efficient energy recovery (34). The reaction between BCl_3_ and H_2_ at low temperatures is thermodynamically impossible. Under 600–750 °C, the high‐energy consumption process on BCl_3_ can direct hydrodechlorination. In addition, substances containing a negative hydrogen charge, such as sodium hydride, tributyltin hydride, or organosilane, also prone to hydrodechlorination. The reaction of BCl_3_ hydrodechlorination is a problematic scientific task. Easier regeneration methods need to be developed to produce hydride containing borane species.

#### 4.1.3 Regeneration of AB with Lithium Aluminum Hydride

**Figure 35:**
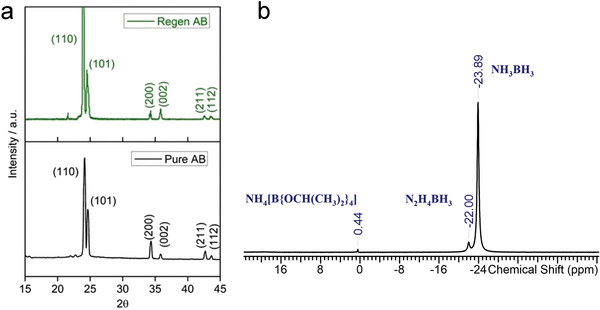
a) XRD comparison between regenerated AB and pure AB was conducted. Reproduced with permission.^[^ ^291^ ^]^ Copyright 2021, Wiley‐VCH. b) ^11^B Nuclear magnetic resonance (NMR) spectrum of regenerated AB. Reproduced with permission.^[^ ^292^ ^]^ Copyright 2021, Elsevier.

The alcoholysis byproduct, ammonium tetramethylborate (NH_4_B(OMe)_4_), can regenerate AB under the reduction of lithium aluminum hydride. Detailed reaction equations: NH_4_B(OMe)_4_ + LiAlH_4_ + NH_4_Cl → NH_3_BH_3_ + Al(OMe)_3_ + LiCl + MeOH +NH_3_ +H_2_. As shown in Figure 33a, the XRD pattern of regenerated AB matches with that of pure AB, with the charac(110), (101), (200), (002), (211), and (112).^[^ ^291^ ^]^ This support also promotes the dehydrogenation and regeneration of AB. After the modified bentonite loading, the AB dehydrogenation efficiency increased from 35 to 71%, and the regeneration efficiency increased from 32 to 67%. N_2_H_4_BH_3_ formation was also observed during the regeneration of NH_4_B(OMe)_4_ (**Figure** **35**a,b).

### 4.2 Hydrogen Production and Regeneration Systems Design

#### 4.2.1 Design of Hydrogen Production and Regeneration System in Aqueous Environment

**Figure 36:**
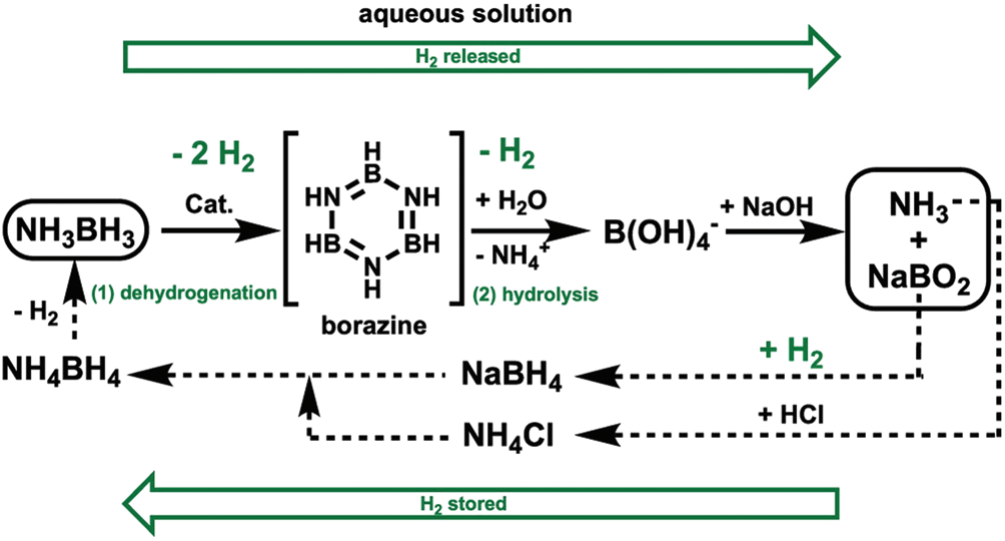
Illustration of dehydrogenation and regeneration system under hydrolytic conditions. Reproduced with permission.^[^ ^293^ ^]^ Copyright 2020, American Chemical Society.

Tuba et al. presented a detailed AB dehydrogenation and regeneration process in an aqueous environment (**Figure** **36**).^[^ ^293^ ^]^ The specific process is as follows: First, hydrogen is directly released through the borazine intermediate formed in the aqueous solution. Efficient catalysts accelerate hydrogen release. B(OH)_4_^−^ is then converted to NaBO_2_ in an alkaline NaOH environment. NaBO_2_ is converted into NaBH_4_ and NH_4_Cl in the presence of H_2_ and HCl. Subsequently, NaBH_4_ and NH_4_Cl are combined to regenerate NH_4_BH_4_. Finally, one molecule of H_2_ is removed using reasonable measures to obtain regenerated NH_3_BH_3_. The regeneration of borate‐based AB has the advantages of no harm and simple experimental operation, however, exploring an efficient catalyst based on theory is still an urgent need.

#### 4.2.2 Regeneration of AB through Hydrolysis under Alcohol‐Based Conditions

**Figure 37:**
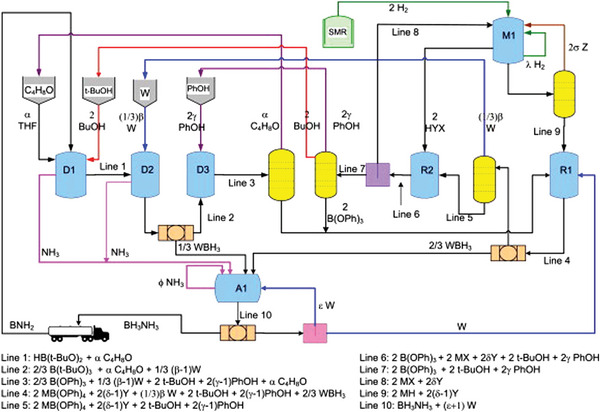
Flow chart of AB alcohol‐based regeneration scheme. Reproduced with permission.^[^ ^294^ ^]^ Copyright 2012, Elsevier.

**Figure 38:**
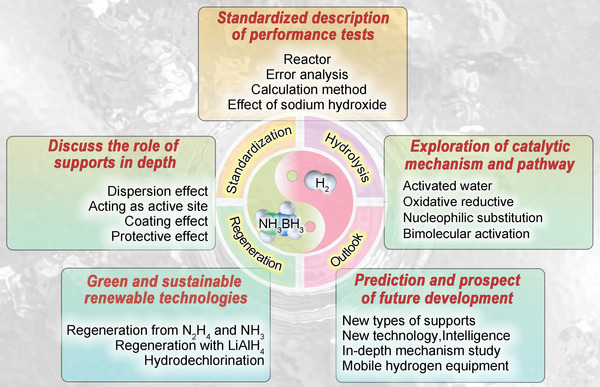
Standardization, hydrolysis, regeneration, and outlook of AB hydrolysis.

Hua et al. studied three promising AB regeneration schemes (**Figure** **37**). The first uses thiols to remove AB waste, and formic acid to end the fuel cycle. The second solution used alcohol to remove AB waste. The third option is a single‐reactor process that uses hydrazine to regenerate AB. **Figure** **38** shows the flow chart of the alcohol regeneration scheme. The four parts are (1) digestion, (2) metal hydride formation, (3) reduction with metal hydride, and (4) ammonification to form BH_3_NH_3_. The overall energy consumption is then calculated. The recovery efficiency of the well‐to‐tank (WTT) system under no heating conditions is 31%, and that under heating conditions was 37%. Hydrogen production accounts for 46% of the total primary energy, regeneration accounts for 54%, and fuel delivery consumes less than 1% of the total primary energy.

## 5 Prediction on the Future Progress for Hydrogen Generation from AB Hydrolysis

Supported metal‐based catalysts have gained significant attention owing to their unique properties and active centers and their potential applications in catalyzing hydrogen production from liquid hydrogen storage materials. In this review, we first introduced the physicochemical properties and standardized testing procedures for AB, followed by a summary of the catalytic performance of supported metal‐based catalysts over the last decade. We evaluated parameters such as the TOF, *E*_a_, nanoparticle size, and durability. Secondly, we used extensive examples to illustrate the advantages of various supports in catalyst design and their influence on the active center. We analyzed the synthesis strategies and morphologies of noble and non‐noble metal catalysts encapsulated in various materials such as graphite, MOF, metal oxides, carbon nitride, MoC, CNTs, h‐BN, zeolite, CDs, and MXenes. Supported catalysts have many advantages over metal NPs, including enhanced stability and recyclability, maximized electronic interaction between the metal NPs and active phase materials, and adjusted adsorption state of the reactants. Thirdly, we emphasize a comprehensive analysis of the development and hydrolysis pathway of the AB hydrolysis and the positive optimization of the hydrolysis mechanism by active supports. Finally, we discuss the feasibility of recycling and reusing AB using organic and inorganic methods. Figure 38 provides a comprehensive summary of hydrogen production from AB. Despite significant progress made over the past decade, there are still challenges to the widespread use of loaded AB hydrolytic material. The following is a brief list of these challenges and their potential solutions.

### 5.1 Development of Novel Supported AB Hydrolysis Catalyst

Rhodium, ruthenium, palladium, and platinum are commonly used as noble metal catalysts, but they are costly and unstable for long‐term AB hydrolysis. Because of their low activity, cobalt, nickel, copper, iron, and other non‐noble metal catalysts cannot take the lead in this regard. To develop new catalysts with better performance, it is essential to combine the strengths of both noble and non‐noble metals to create new active centers. One way to achieve this is to construct polyatomic groups that mimic the atomic composition and arrangement of precious metals and form outer electron orbitals similar to those of noble metals. To replace or partially replace precious metals with non‐precious metals, it is crucial to optimize experimental parameters such as the metal support ratio, temperature range, solvent ratio, metal precursors, and stabilizers.

### 5.2 Role of Support in AB Hydrolysis System

The active phase of a catalyst plays a critical role in promoting the optimal dispersion and stability of NPs, preventing deactivation by limiting unnecessary leaching or sintering, facilitating reactant mixing, and optimizing the adsorption state to avoid secondary reactions. Currently, mainstream academic consensus favors bimolecular activation, in which AB and water molecules are activated separately. Therefore, the design of double‐active sites to facilitate this process is crucial. To achieve this, a comprehensive design of metal‐active supports is necessary, where metal NPs activate AB and rupture B—H bonds, while active phase supports facilitate the rate‐breaking step of water molecule activation and O—H rupture. 1D supports such as CNTs and CDs are primarily used for loading precious metals such as Ru and Pd, as well as non‐precious metals, such as Co and Ni. 2D supports, like GO, g‐C_3_N_4_, h‐BN, MoC, and MXene, are suitable for loading a broad range of metals. Mechanisms between the metal and supports, such as strong metal‐support interactions and surface functionalization strategies, are also being investigated. 2D support materials, such as, MOF and zeolites, mainly encapsulate metals in their corresponding pore channels, providing long‐term activity. Novel active supports such as MXenes and CDs, can optimize charge transfer between active regions, offering new catalytic models. Modifying the surface terminal of MXenes allows for the adjustment of the electronic structure of the active metal site, affecting the atomic bond distance and bond energy of the adsorption intermediates. Using MXenes as a support and transition metal to construct a dual‐active center catalytic material is expected to result in high‐activity, high‐stability, and low‐cost catalytic material for hydrogen production from AB hydrolysis.

### 5.3 New Technology

Advanced in situ and time‐resolved characterization techniques have been developed, along with theoretical calculations, to extensively investigate the mechanism of action and catalysis of metals and supports in the AB hydrolysis system. Real‐time monitoring of the active sites was conducted using in situ measurements, such as HAADF‐STEM, XAS, and Raman spectroscopy. Additionally, instantaneous characterization methods, such as picosecond lasers, have been employed to observe the AB hydrolysis reaction step at the catalytic site with atomic spatial resolution and millisecond time resolution. An experimental parameter classification model has been established to optimize the catalyst preparation technology, which is challenging but potentially beneficial.

### 5.4 Controllable Cost and Large‐Scale Application

Hydrogen production using AB has numerous advantages, such as high efficiency, safety, and rapid dehydrogenation at room temperature. However, the current production cost of AB remains very high, and its reduction is crucial. There are three ways to reduce the cost of the catalyst design. First, the amount of precious metals used can be reduced, and these can be combined them with inexpensive non‐precious metals. The rational use of precious and non‐precious metals can improve the activity of catalysts and reduce their cost.^[^ ^257^, ^267^ ^]^ Second, inexpensive non‐precious metals are directly found on earth as catalysts, while developing rapid mass synthesis strategies to reduce costs. Third, metal‐free catalysts use cheaper materials such as Si, C, and N to reduce costs.^[^ ^32^, ^295^ ^]^

### 5.5 In‐Depth Mechanism Study

However, the mechanism of AB hydrolysis is currently a topic of controversy. Although several catalytic mechanisms have been proposed based on density through density functional theory calculations, there is a lack of corresponding experimental evidence. The correlation between the electronic structure and catalytic performance is not yet fully understood, and the influence of morphology on catalytic performance remains unclear. In addition, there is a lack of means to measure and confirm reaction intermediates and transition states. Distinguishing between multiple AB hydrolysis mechanisms remains challenging, particularly because there may be multiple reaction mechanisms may occur on the same catalyst. Therefore, designing test schemes in situ and under working conditions is crucial to comprehensively studying the in situ mechanism.

### 5.6 Circulation and Regeneration of AB

AB must be efficiently circulated and regenerated to facilitate the widespread use of AB as a liquid hydrogen storage material. Unfortunately, the primary obstacle to achieving this goal is the high cost of recycling AB. There is a critical need to develop of new cost‐effective recycling systems and catalysts to enable efficient and sustainable AB regeneration.

### 5.7 The Introduction of Artificial Intelligence Technology

Artificial intelligence can be utilized to study and optimize the relationship between the composition, structure, process, and properties of catalytic hydrolysis materials. To efficiently obtain a large amount of experimental data, a parameter hierarchical optimization model can be constructed. High‐throughput preparation and testing techniques were employed to analyze the experimental results. Finally, an explainable model theory must be established to derive reliable experimental conclusions for AB hydrolysis.

### 5.8 Exploration and Utilization of Portable Mobile Hydrogen Storage Energy

Implementation of a portable power supply system utilizing liquid hydrogen storage technology is of immense economic importance and holds great research significance. This technology is expected to find applications in various domains such as hydrogen‐powered drones, fuel cell mopeds, and sizeable hydrogen‐powered buses.

## Conflict of Interest

The authors declare no conflict of interest.
